# Processing Relative Clause Extractions in Swedish

**DOI:** 10.3389/fpsyg.2017.02118

**Published:** 2017-12-07

**Authors:** Damon Tutunjian, Fredrik Heinat, Eva Klingvall, Anna-Lena Wiklund

**Affiliations:** ^1^Centre for Languages and Literature, Lund University, Lund, Sweden; ^2^Department of Languages, Linnaeus University, Växjö, Sweden

**Keywords:** eyetracking, sentence processing, filler-gap, integration, island constraints, plausibility, relative clause, Swedish

## Abstract

Relative clauses are considered strong islands for extraction across languages. Swedish comprises a well-known exception, allegedly allowing extraction from relative clauses (RCE), raising the possibility that island constraints may be subject to “deep variation” between languages. One alternative is that such exceptions are only illusory and represent “surface variation” attributable to independently motivated syntactic properties. Yet, to date, no surface account has proven tenable for Swedish RCEs. The present study uses eyetracking while reading to test whether the apparent acceptability of Swedish RCEs has any processing correlates at the point of filler integration compared to uncontroversial strong island violations. Experiment 1 tests RCE against licit *that*-clause extraction (TCE), illicit extraction from a non-restrictive relative clause (NRCE), and an intransitive control. For this, RCE was found to pattern similarly to TCE at the point of integration in early measures, but between TCE and NRCE in total durations. Experiment 2 uses RCE and extraction from a subject NP island (SRCE) to test the hypothesis that only non-islands will show effects of implausible filler-verb dependencies. RCE showed sensitivity to the plausibility manipulation across measures at the first potential point of filler integration, whereas such effects were limited to late measures for SRCE. In addition, structural facilitation was seen across measures for RCE relative to SRCE. We propose that our results are compatible with RCEs being licit weak island extractions in Swedish, and that the overall picture speaks in favor of a surface rather than a deep variation approach to the lack of island effects in Swedish RCEs.

## Introduction

It has long been observed that a number of constraints apply to the formation of long-distance filler-gap dependencies (Ross, 1967; Chomsky, 1973, 1986; Rizzi, 1990; Boeckx, 2012). One such constraint is relevant to complex noun phrases. Consider the examples in (1). In sentences like (1a), the NP object, the so-called “filler” (*those kinds of flowers*), can be extracted from its thematic position, the “gap” (*[–]*), in the argument clause and “moved” long-distance to a left-peripheral position in the matrix clause. However, a similar extraction is prevented when the resulting gap would be in a relative clause (RC) that is part of a complex noun phrase (1b).

(1)  a.  *[Those kinds of flowers]_*i*_, *I* saw that a man sold [–]_*i*_ in the square*.
      b. ^*^[Those kinds of flowers]_*i*_, I saw a man that sold [–]_*i*_ in the square.

We will henceforth refer to the latter extraction as *RC extraction* (RCE). Complex noun phrases with RCs are standardly considered to be *strong islands* for extraction: syntactic domains from which extraction is not possible (Ross, 1967). The unacceptability that arises from an illicit extraction (e.g., 1b) is commonly referred to as an *island effect*.

Island effects have been observed in a great number of languages, spanning numerous language families. Consequently, island constraints have generally been considered to be universal. And yet, a number of recent studies have presented evidence that island effects may show some form of cross-linguistic variation (e.g., Phillips, 2013; Sprouse et al., 2016), raising the question as to whether the constraints themselves are variable. Phillips (2013) presents two general approaches to account for variation in island effects. The first, which we will call the *surface variation* approach, involves appealing to independently motivated syntactic properties to demonstrate that the violation is only apparent, thus maintaining the universality of island constraints. For example, apparent cases of RC extraction in the East Asian languages have been explained by positing control of a null subject inside the RC by a topic NP outside the RC (Han and Kim, 2004). The second approach entails allowing for *deep variation* in island constraints to exist between languages. That is, languages may show true underlying variance with regard to which structural configurations constitute syntactic islands (see e.g., Rizzi, 1982).

Certain languages have proved resistant to any surface approach for explaining the absence of expected island effects. Swedish provides just such a case. It has been widely attested that RCEs are acceptable in Swedish (2), as well as in the other Mainland Scandinavian languages (Erteschik-Shir, 1973; Allwood, 1976; Taraldsen, 1981; Engdahl, 1982, 1997; Christensen and Nyvad, 2014), with examples being found in both spoken and written language (Lindahl, 2017)^1^.

(2) [Såna blommor]_*i*_            såg jag en man som sålde [–]_*i*_ på
     [Those kinds of flowers]_*i*_  saw I     a   man  that  sold   [–]_*i*_  in *torget. (Swedish)*
     square-the.
     “Those kinds of flowers, I saw a man that sold in the square.”

To account for this apparent acceptability, a number of surface variation analyses have been proposed, including a discourse organization account (Erteschik-Shir, 1973), a covert resumption analysis (Cinque, 1990), and a small clause structure analysis (Kush et al., 2013). However, it has been demonstrated that none holds up under closer scrutiny (Engdahl, 1997; Boeckx, 2012; Christensen and Nyvad, 2014; Heinat and Wiklund, 2015; Lindahl, 2015; Müller, 2015). Thus, from a syntactic perspective, it remains unclear whether the apparent acceptability of Swedish RCEs represents a case of deep variation in island constraints or whether the issue continues to be a matter of finding a tenable surface structure account for the data.

As a means of guiding future syntactic research, the current study uses eyetracking while reading to investigate whether the apparent acceptability of Swedish RCEs has any processing correlate at the point of filler integration (the gap) compared to uncontroversial strong island violations. One possibility is that although RCEs are intuitively acceptable in Swedish, they may actually be processed more similarly to island-like structures. This would suggest that their apparent acceptability is attributable to some type of off-line repair mechanism, perhaps dependent on language specific discourse organization or contextual factors, and that the exceptionality of these structures therefore does not comprise a case of deep variation in island constraints. A second possibility is that the processing of Swedish RCEs may match their apparent acceptability, and that they will be processed more similarly to non-islands. Such an outcome would suggest that either a tenable surface account is still needed or that deep variation, on some level, does indeed exist.

No previous online processing studies of Swedish RC islands exist; however numerous studies have used online methods to investigate filler-gap dependencies that extend into island domains in other languages (primarily English). One point of general consensus from this body of research is that the parser appears to respect island constraints at the earliest stages of processing, effectively blocking the formation of filler-gap dependencies that extend to island domains while allowing them in licit structures (Stowe, 1986; Bourdages, 1992; Traxler and Pickering, 1996; McElree and Griffith, 1998; Omaki and Schulz, 2011; Omaki et al., 2015; cf. Freedman and Forster, 1985; Pickering et al., 1994; Phillips, 2006; Wagers and Phillips, 2009).

For example, Traxler and Pickering (1996) used eyetracking while reading to test whether the parser immediately employs structural island information to preclude filler-verb integration in sentences involving extraction from English RCs, which are known to be strong islands.

(3)  a.  *We like the* {*book/city*} *that the author who wrote [–] unceasingly and with great dedication saw [–] while waiting for a contract*.
      b.  *We like the* {*book/city*} *that the author wrote [–] unceasingly and with great dedication about [–] while* waiting for a contract.

In their design, the optional transitivity of the embedded verb (*wrote*) creates a temporary ambiguity as to the actual location of the extraction site (gap) of the filler, which is ultimately disambiguated at a second gap position. By manipulating the plausibility of the NP filler (*book* vs. *city*) and monitoring for plausibility mismatch effects at the embedded verb (first gap), they were able to test whether the parser immediately acts to inhibit dependency formation at the embedded verb in strong islands (3a), relative to a non-island control (3b). They found that plausibility effects, manifest as longer fixation durations for implausible filler-verb relations (*city-wrote*) than plausible ones (*book-wrote*), were present for the non-island condition in both first fixation durations and total fixation durations at the embedded RC verb, but not for the island condition. From this, Traxler and Pickering drew two primary conclusions. First, that the parser actively attempts to complete unbounded dependencies and fill gaps at the earliest possible moment, regardless of the presence of an ambiguity. Second, that dependency formation is blocked in cases where the parser is unable to construct a dependency that will result in a licit constituent, such as those involving RC island extractions. The differential nature of this general finding in regards to islands and non-islands thus provides a heuristic for determining the island status of a given construction from a processing perspective.

In the current study, we enlist use of a structural manipulation (Experiment 1), as well as a variant of the plausibility heuristic from Traxler and Pickering (1996) (Experiment 2) to address the island status of Swedish RCEs and thereby direct future work on variation in island effects.

## Experiment 1

Experiment 1 uses an eyetracking while reading paradigm to investigate whether Swedish RCEs (4b) elicit processing costs similar to licit extractions from *that*-clauses (TCE) (4a) or instead pattern closer to extractions from non-restrictive RCs (NRCE) (4c), which are reported to be strong islands in Swedish (Engdahl, 1997; Teleman et al., 1999; Platzack, 2000).^2^ For our comparison, we assume a link between differential results and successful or unsuccessful integration of an extracted filler (*Såna där gamla skottkärror/“*such old wheelbarrows”) at the first embedded verb in the dependent clause (*tvättade/“*washed”) and the following two-word PP region (*på bensinmacken*/“at the gas station”).

(4)   a. TCE
           Såna där gamla skottkärror     såg  jag  att  en  man
           such         old      wheelbarrows  saw I      that  a    man
           *alltid   tvättade [–]*
           always washed   [–]
           *på bensinmacken  när   han var  ledig*.
           at  gas-station-the  when  he   was free.
           “Such old wheelbarrows, I saw that a man always washed at the gas station in his spare time.”
       b. RCE
           Såna där gamla skottkärror    såg  jag en man som
           such         old      wheelbarrows saw  I    a   man  that
           *alltid   tvättade [–]*
           always washed   [–]
           *på bensinmacken  när    han var ledig*.
           at  gas-station-the  when  he   was free.
           “Such old wheelbarrows, I saw a man who always washed at the gas station in his spare time.”
       c. NRCE
           Såna där gamla skottkärror    såg  jag  en  man  som
           such         old      wheelbarrows saw I       a    man   that
           *förresten   tvättade [–]*
           by-the-way washed  [–]
           *på bensinmacken  när   han  var  ledig*.
           at gas-station-the   when  he   was  free.
           “Such old wheelbarrows, I saw a man who by the way washed at the gas station in his spare time.”
       d. PCRCE
           Såna där gamla skottkärror    såg  jag  en  man  som
           such         old      wheelbarrows saw I       a   man   that
           *alltid   stod    och*
           always stood and
           *tvättade [–] på  bensinmacken   när   han  var  ledig*.
           washed  [–]  at    gas-station-the   when he    was  free.
           “Such old wheelbarrows, I saw a man who always stood washing at the gas station in his spare time.”

We also include an intransitive control condition, PCRCE (4d) (also allowing extraction), which is identical to RCE, but for which the embedded transitive verb is pseudo-coordinated with a preceding intransitive light verb (*stod*/“stood”) (Teleman et al., 1999; Wiklund, 2007). Thus, for PCRCE the first embedded verb region differs from the verb in the other three conditions (*stod*/“stood” vs. *tvättade*/“washed”). Being intransitive, the first embedded verb in PCRCE is expected to provide a baseline for what processing would look like in cases where no integration is attempted (cf. Omaki et al., 2015). In this way, PCRCE is expected to help assure that any noted differences between RCE and NRCE are related to integration and islandhood and do not solely represent any independent cost arising from structural complexity.

Although the words in our analysis regions are held maximally constant across structures (excepting the first embedded verb in the PCRCE control), there are differences in earlier sentential regions, which have the potential to affect fixation durations at the embedded verb. For example a different adverb is used for NRCE than in the other conditions (to induce the island), and relativization is present in all conditions except TCE. To best circumvent any potential confounds related to these differences, our approach takes as a starting point the widely accepted generalization that different eyetracking measures reflect different costs that are likely to emerge in the course of processing (see discussion in Clifton et al., 2007). By enlisting different measures together with an appropriate control condition, we expect to be able to tease apart structural effects related solely to differences in structural complexity (e.g., additional syntactic heads or underlying structure) and effects related to complexity and integration (i.e., actual dependency formation over intervening material), the latter of which we will assume more accurately reflects islandhood costs. We detail our assumptions about these costs below, and then follow with description of the eyetracking measures used in our study and their relation to different costs, as well as our specific predictions for each condition and measure.

Our cost assumptions are largely cast within Dependency Locality Theory (DLT) (Gibson, 1998, 2000), which posits that sentence complexity related to dependency formation and integration is affected by increases in memory load and can be coarsely determined via the number of new discourse referents intervening between a head/antecedent and its integration site. To the extent that DLT specifies, the intervening material in our sentences is equivalent between conditions. However, it has been proposed that the DLT may not sufficiently predict processing costs on its own. For example, Demberg and Keller (2007) argue that an extra source of processing complexity arises when the verb-filler dependency extends over a clause boundary; and Alexopoulou and Keller (2007) provide evidence that other types of heads, such as auxiliary verbs, contribute to complexity. Our materials bear features that are similarly not accounted for in the DLT, but which could also have relevance to complexity and integration. First, RCE, PCRCE, and NRCE all contain relativization, whereas our TCE condition does not. Relativization has been demonstrated to increase memory load, which may become manifest as locality effects if working memory resources are sufficiently taxed (Levy and Keller, 2013). Second, our NRCE condition is likely to have additional underlying structure (Haegeman, 2002, 2010; de Vries, 2012), which may or may not contribute to its overall complexity (depending on what counts as “complex”), but which is arguably instrumental to its structural island status. Both of these differences could accrue additional cost. In addition, it has been argued that processing is best explained not only by locality principles but also by expectation (e.g., Demberg and Keller, 2007; Levy et al., 2012; Levy and Keller, 2013). Our PCRCE control utilizes an intransitive verb in the critical region, whereas RCE, TCE, and NRCE all use the same transitive verb, a difference which could conceivably be reflected in expectation-based costs. We return to these differences below, when stating our predictions, but before doing so, it is necessary to first present the eyetracking measures used in the study.

It is generally held that different eyetracking measures reflect different processing costs. We analyze four such measures in our experiment. First fixation duration (the duration of the first fixation on a region during first pass reading) has been shown to be sensitive to lower level processes such as word recognition and general increases in complexity (Rayner et al., 1989; Hyönä and Vainio, 2001; Vainio et al., 2003; Staub et al., 2006; see review in Clifton et al., 2007). Gaze duration (the sum of all fixations in a region during first pass reading) is also often treated as a measure of complexity, but it has additionally been shown to reflect costs associated with actual integration during dependency formation (Rayner et al., 2004; Staub et al., 2007; Warren and McConnell, 2007; Demberg and Keller, 2008). Likewise, measures targeting regressive eye movements such as regression path duration (the sum of all fixations on the target region plus regressive fixations to prior regions until the target region is exited to the right during first pass reading) are also thought to reflect integrative stages (Rayner et al., 2004; Traxler et al., 2005; Gordon et al., 2006; see discussion in Staub, 2010). Finally, total duration (the sum of all fixations on a region) is held to reflect textual integration processes, lexical and syntactic/semantic processing, and general comprehension difficulty, and thus the overall holistic, cohesive success of the sentence (Boston et al., 2008; Demberg and Keller, 2008). The first two measures are often referred to as “early” measures and the last two as “late” measures, as a generalization about the stages of processing they roughly represent.

We turn now to our predictions for Experiment 1, beginning with the clearest contrast, that between TCE and NRCE. Though not tested directly in their studies, both Omaki and Schulz (2011) and Traxler and Pickering (1996), using materials roughly similar to our TCE non-island and NRCE strong island conditions, showed a numerical advantage for their plausible non-islands relative to their plausible islands. This pattern was seen in first fixation durations and total durations at the verb region for Traxler and Pickering (1996), and in self-paced reading RTs (roughly analogous to eyetracking measures marking integration) in post-verb regions in Omaki and Schulz (2011). Given these numerical distinctions and the differences noted above for NRCE relative to TCE (relativization, additional structure/complexity), which acting together we assume contribute to its islandhood status, we expect that TCE will show greater facilitation than NRCE at the verb region (and a following PP region), across the four eyetracking measures^3^. However, we also expect that this difference will not always be for the same reason. In order to make direct comparisons between islands and potential non-islands, it is critical to first tease apart costs that are purely related to structural complexity and those that involve complexity insofar as it relates to integration and dependency formation, the latter of which we view as a construal of islandhood. For this reason, we include our PCRCE control.

The embedded intransitive verb in PCRCE is arguably less structurally complex than the corresponding transitive verb in NRCE, TCE, and RCE. PCRCE should thus show the shortest durations (after adjusting for word length via a residualization procedure-described in section Data Analysis) for any measure solely reflecting complexity (e.g., first fixation duration). In contrast, for measures involving actual integration and dependency formation, encountering and accessing the argument structure of the intransitive verb in PCRCE should comprise a costly breach of expectation, provided such integration is attempted, as it lacks the necessary transitivity to accommodate the open dependency. The presence of a cost for PCRCE relative to the transitive conditions would thus provide verification that processes of integration and dependency formation were at least considered by the parser. In turn, any cost then shown by NRCE relative to TCE for that measure could be attributed to integrative processes and issues with dependency formation related to the islandhood of NRCE. And most importantly, the patterning of RCE could be assessed relative to TCE and NRCE. Based on the attested acceptability of RCEs, our prima facie expectation is that RCE will pattern together with TCE as faster than NRCE in integrative measures involving dependency formation, as confirmed by the patterning of PCRCE, and that this will be interpretable as support that RCE involves a licit extraction.

### Method

#### Participants

Forty-eight native speakers of Swedish participated in the study in exchange for a cinema ticket. All participants were aged 18–40 and were screened to not have native-like levels in any other language. Three participants were excluded for scoring <80% correct on the comprehension questions, leaving 45 for analysis.

#### Materials

Eighty long-distance sentence items of the type in (4) were constructed, each appearing in four structural variants (Structure). A full list of the critical items can be found in the Supplementary Material for this paper. Items were rotated across four presentation lists using a Latin Square and then randomized. Each participant thus only saw one variant per item. All critical items consisted of a set of ordered regions for which there was an equivalent number of words within all but the final wrap-up regions. Regions were streamlined to closely match in constituent class and features across items and conditions.

To force a non-restrictive reading of the RC for condition (4c), the adverb *förresten/*“by the way” was inserted before the embedded verb. To maintain parallelism in constituency across the structural variants, sentential adverbs (from a class that does not induce non-restrictive RC readings) were inserted at the same position for the other structures (*alltid*/“always” in 4a, 4b, and 4d). The inclusion of an adverb also served the secondary purpose of buffering our regions of interest (the embedded verb and the following PP) from any potential residual effects that might arise from the difference between (4a), in which the complementizer *att*/“that” precedes the NP, and (4b–d), where the relativizer *som*/“that” follows the NP. This is especially important given that processing difficulty has been shown to appear at the noun phrase subject of an object RC (see discussion in Staub, 2010).

The embedded verb (e.g., *tvättade*/“washed”) always comprised a mono-transitive verb, excepting the control condition, PCRCE, (4d), for which the mono-transitive verb was pseudo-coordinated with a preceding intransitive light verb (*stod*/“stood”). All verbs (main and embedded) had a raw lemma frequency of at least 3,000 in 4.90 billion tokens from an aggregated subset of corpora extracted from Korp (Borin et al., 2012), the corpus infrastructure of Språkbanken, the Swedish Language Bank.

High transitional probabilities have been shown to have a facilitatory effect on a word's first fixation durations and gaze durations (Ehrlich and Rayner, 1981; Rayner and Well, 1996; McDonald and Shillcock, 2003a,b; Ashby et al., 2005; Frisson et al., 2005; review in Clifton et al., 2007). And yet, in the current design, maintaining streamlined probabilities was impractical, given our other design parameters. To address this, we again used Korp to determine the log transitional probability (Freq) of the filler noun following the embedded verb in each of our sentential items, such that the probability could later be modeled into our statistical analyses. Korp is not tagged sufficiently to determine such probabilities, thus for this measure we searched for the number of instances in which the embedded verb and noun co-occurred within a range of zero to three words following the verb, as an approximation of verb-object relationships. We then divided this number by the raw lemma frequency of the verb and took the log of the result. In order to reduce the chance that word-specific, selectional biases or transitional probabilities would differentially affect reactivation of a filler, all filler NPs used in the sentence materials were selected to be semantically possible objects of both the matrix and embedded verb with which they appeared in the critical items, excluding the most high frequent ones.

Next, to ensure that our fillers were all acceptable potential objects of our embedded verbs, we tested the consistency of the overall coherence of the semantic relationships present in our sentences, inclusive of the pragmatic goodness-of-fit of the filler to the embedded verb. For this measure, 24 participants (not taking part in the main experiment) completed acceptability/naturalness ratings for 80 non-extracted, embedded *that*-clause versions of each of our sentence items, as in (5), using a seven-point Likert scale ranging between one (*helt onaturlig*/“completely unnatural”) and seven (*helt naturlig*/“completely natural”). Forty-five distractors, designed to provide instances of pragmatically good, degraded, and bad sentences were randomly interspersed with the critical items.

(5) *  Jag såg att  en man tvättade såna skottkärror    på*
       I      saw that a   man washed   such  wheelbarrows at
       bensinmacken.
       *gas-station-the*
       “I saw that a man washed such wheelbarrows at the gas station.”

Mean responses to the critical items (*M* = 5.08, *SD* = 0.97) demonstrated that, overall, the un-extracted forms of our sentences were consistently judged to be on the pragmatically acceptable/natural end of the spectrum, which we take to indicate that filler-verb relationships and semantic cohesion were acceptable throughout our materials.

As a final step, we collected normative data for our critical sentences, allowing us to establish a set of ratings for the materials and providing a point of comparison that was not based solely in the intuitive acceptability of the structures in question. Thirty-eight participants (not taking part in the main experiment or pragmatic normative study, and prescreened to have only Swedish as a native language) completed the study via Google Forms using their own computers in exchange for a movie ticket. The four structural variants for our 80 critical items and 60 distractor items were rotated across four presentation lists. Each participant was assigned to one of the four lists and asked to rate each sentence on a scale of 1 (*helt oacceptabel/*“completely unacceptable”) to 7 (*helt acceptabel/*“completely acceptable”). Each list began with three practice items. The analysis was conducted using the lme4 package for linear mixed models (Bates et al., 2014), with random intercepts specified by Subject and Item, and random slopes for Structure specified by Subject in the final fitted model (slopes were excluded by Item, as their inclusion caused convergence errors).

Mean ratings and the output of a linear mixed model analysis of *z*-transformed ratings are presented respectively in Table 1 and Table 2. Both RCE and TCE received significantly higher ratings than the island condition, NRCE. In addition, PCRCE patterned alongside RCE, as would be expected, given that they are similar structures, apart from the pseudo-coordinated light verb. RCEs were also seen to be significantly less acceptable than TCEs, which is not surprising, given that relativization is a complex operation. Identical statistical patterns were observed in a cumulative link model analysis (Christensen, 2015) (not presented here). The finding that RCE acceptability fell below the mid point is somewhat in opposition to intuitive reports that such structures are acceptable (Erteschik-Shir, 1973; Allwood, 1976; Engdahl, 1982), but matches what has been observed in one other experimental context (Müller, 2015). In fact, even the TCE sentences failed to obtain ratings extending far beyond the midpoint. We suspect that overall low acceptability could be due to (1) the length and complexity of the sentences and (2) the difficulty of finding an appropriate context for all of our sentences Nevertheless, RCEs were significantly more acceptable than island conditions, and a tendency toward rated unacceptability only serves as a bias against any finding that they would pattern together with non-islands.

**Table 1:** Experiment 1 mean sentence ratings and standard deviations.

**Structure**	**Mean (SD)**
TCE	4.85 (1.72)
PCRCE	3.08 (1.66)
RCE	3.11 (1.65)
NRCE	2.50 (1.39)

*Scale: 1 (helt oacceptabel/“completely unacceptable”) to 7 (helt acceptabel/“completely acceptable”)*.

**Table 2:** Experiment 1 linear mixed models analysis of z-score transformed sentence ratings.

**Fit: lmer(RatingZ ~ Structure + (1| Subject) + (1 + Structure | Item))**			
**Linear Hypotheses**	**Estimate**	**Std. Error**	***z*****-value**
RCE-NRCE = 0	0.1500	0.0195	7.69^***^
TCE-NRCE = 0	0.5707	0.0245	23.34^***^
PCRCE-NRCE = 0	0.1428	0.0189	7.56^***^
RCE-TCE = 0	−0.4207	0.027	−15.57^***^
RCE-PCRCE = 0	0.0073	0.0191	0.38
TCE-PCRCE = 0	0.4279	0.0248	17.26^***^

p-values: “^***^” 0.001 “^**^” 0.01 “^*^” 0.05 “†” 0.1.

*p-values adjusted for multiple comparisons using the ”single-step“ method in Hothorn et al. (2016)*.

*Scale: 1 (helt oacceptabel/“completely unacceptable”) to 7 (helt acceptabel/ “completely acceptable”)*.

Sixty distractor items were constructed to mask the structural manipulation, of which 40 were fully grammatical and 20 were ungrammatical. Twenty of the grammatical distractors included topicalization of an object with a stranded RC (not involving extraction) and 20 included the adverb *förresten*.

#### Procedure

Eye-movements were recorded using a tower-mounted Eyelink 1000 system (SR Research) and a 24-inch, 1,920 × 1,080, 144 Hz, ASUS monitor. Participants completed three practice trials prior to beginning the experiment. Trial items were displayed 500 ms after fixation on a gaze-contingent cross that was located at the left edge of each sentence. One-third of all items were followed by a yes/no comprehension question.

### Data analysis

Prior to analysis, an automatic procedure incorporated fixations <80 ms into larger fixations within a one-character range and then deleted fixations of <40 ms within three characters of any other fixation (Rayner and Pollatsek, 1989). Single fixations exceeding 800 ms were also removed (Staub, 2007). The remaining values were log-transformed to correct for positive skewness (Levine and Dunlap, 1982).

To investigate the hypothesis that RCE will pattern together with the non-island condition (TCE) at the point of filler-gap integration, we first defined two regions of interest: Region 1 (R01): the embedded verb (*tvättade*/“washed” in 3a-c; *stod*/“stood” in 4d) and Region 2 (R02): the following prepositional phrase (*på bensinmacken*/“at the gas station” in 4a-c). The embedded verb is the primary region where we expect to see effects of filler-verb integration. Analysis at the PP also allows us to monitor any delayed or long-lasting effects from the first region that may be present.

To account for word length differences at the embedded verb region stemming from the contrast between the length of lexical verbs in (4a-c) and the length of the light verbs in (4d), we followed a standard procedure of using each region's character count to regress and residualize the log fixation durations for each separate measure against predicted per-character fixation durations (Ferreira and Clifton, 1986; Trueswell et al., 1994; Rayner, 1998). To present a unified measure for analysis, the same procedure was performed for fixations in the PP region. Constituent differences between PCRCE and the other levels of Structure (a conjunction and a second embedded verb in PCRCE vs. a PP in all other variants) precluded any logical inclusion of PCRCE for analysis at the PP region, and thus only the three remaining conditions were analyzed for that region.

We used linear mixed models to analyze fixations for four measures: first fixation duration, gaze duration, regression path duration, and total durations, at R01 and R02. To fit the fixed component, we began with a “beyond optimal model” (Zuur et al., 2009) which included one categorical predictor Structure, and two centered, continuous variables Trial and Freq as fixed factors, plus the Trial by Structure interaction terms. Trial comprised an index of the order of presentation of all critical trials in a run (ordinal data, treated here as continuous). Freq comprised the transitional probability of the filler noun following the embedded verb in each of our sentential items, as described above. Following Barr et al. (2013), we specified a random effect structure that included intercepts for subject and item, and by-item and by-subject slopes for Structure. The random component was reduced in a stepwise fashion until it reached convergence. From this, we then determined the fixed effects structure, again enlisting a top down strategy, comparing log likelihood between models until we identified the best fit model that minimally included Structure. Next, a set of contrasts was constructed using the glht function in the multcomp package (Hothorn et al., 2016). To reduce the likelihood of Type I error, a “single-step” adjustment for multiple comparisons based on the joint normal or *t* distribution of the linear function was applied to *p*-values. This approach accounts for the correlations between the parameter estimates, yielding smaller *p*-values than the Bonferroni test (Bretz et al., 2016).

### Results

Turning now to the results, we first present eyetracking measures for the embedded verb region (R01) and then for the following PP region (R02). Raw mean fixation durations for Regions 1 and 2 are presented in Table 3. Final model fits and linear mixed models analyses are presented in Tables 4, 5.

**Table 3:** Experiment 1 mean and mean residual fixation durations for Structure.

**Measure and structure**	**Region 01**				**Region 02**			
	**Mean**	**(SE)**	**Mean of residuals**	**(SE)**	**Mean**	**(SE)**	**Mean of residuals**	**(SE)**
**FIRST FIXATION DURATION**								
TCE	224.39	(5.6)	−4.51	(2.4)	231.25	(5.4)	2.1	(2.5)
RCE	230.29	(5.7)	1.33	(2.7)	231.94	(5.4)	2.64	(2.6)
NRCE	241.34	(5.7)	11.89	(2.6)	234.13	(5.6)	5.1	(2.6)
PCRCE	226.1	(6.5)	−6.69	(2.9)	NA	NA	NA	NA
**GAZE DURATION**								
TCE	270.77	(6.9)	−36.41	(5)	364.58	(7.1)	−39.01	(5.6)
RCE	268.2	(6.4)	−36.74	(4.2)	367.01	(7.3)	−33.53	(5.5)
NRCE	279.33	(6.3)	−24.19	(4)	389.55	(7.8)	−13.7	(6)
PCRCE	233.87	(6.6)	42.55	(3.4)	NA	NA	NA	NA
**REGRESSION PATH DURATION**								
TCE	322.56	(11)	−238.11	(14.4)	464.41	(17.3)	−476.44	(22.9)
RCE	348.22	(13.7)	−207.07	(17.5)	506.99	(17.3)	−416.08	(23.2)
NRCE	326.21	(10.2)	−212.57	(13.4)	556.05	(18.7)	−378.31	(24.9)
PCRCE	286.69	(13.5)	210.38	(14.9)	NA	NA	NA	NA
**TOTAL DURATION**								
TCE	427.47	(9.5)	−145.65	(9.4)	588.34	(11.8)	−137.23	(11.7)
RCE	499.65	(11.2)	−64.74	(10.7)	696.56	(16.2)	−25.48	(14.9)
NRCE	575.44	(13.2)	14.44	(12.2)	789.1	(16.3)	65.77	(14.8)
PCRCE	335.85	(7.5)	−51	(7.4)	NA	NA	NA	NA

**Table 4:** Experiment 1 R01 (First embedded verb): Simultaneous tests for general linear hypotheses for linear effects models fitted to log-transformed and residualized fixation durations.

**Linear hypotheses**	**Est**.	**SE**	***z***	**Linear hypotheses (Interactions)**	**Est**.	**SE**	***z***
**FIRST FIXATION DURATION**							
**Model: lmer(ResidualLogFixDuration ~ Structure + Trial + (1 + Structure | Subject) + (1 | Item))**							
PCRCE − NRCE = 0	−0.0777	0.0175	−4.436^***^	NA	NA	NA	NA
RCE − NRCE = 0	−0.0472	0.0139	−3.394^**^				
TCE − NRCE = 0	−0.0702	0.014	−5.002^***^				
RCE − TCE = 0	0.0230	0.014	1.638				
RCE − PCRCE = 0	0.0305	0.0179	1.700				
TCE − PCRCE = 0	0.0075	0.017	0.440				
Trial = 0	0.0006	0.0003	2.537^†^				
**GAZE DURATION**							
Model: lmer(ResidualLogFixDuration ~ Structure^*^Trial + (1 | Subject) + (1 | Item))							
PCRCE − NRCE = 0	0.0758	0.0174	4.351^***^	PCRCE:Trial − NRCE:Trial = 0	−0.0020	0.0007	−2.721^†^
RCE − NRCE = 0	−0.0486	0.0162	−3.001^*^	RCE:Trial − NRCE:Trial = 0	0.0001	0.0007	0.154
TCE − NRCE = 0	−0.0544	0.0161	−3.370 ^**^	TCE:Trial − NRCE:Trial = 0	−0.0004	0.0007	−0.613
RCE − TCE = 0	0.0058	0.0162	0.357	RCE:Trial − TCE:Trial = 0	0.0005	0.0007	0.759
RCE − PCRCE = 0	−0.1244	0.0175	−7.116^***^	RCE:Trial − PCRCE:Trial = 0	0.0021	0.0008	2.836^*^
TCE − PCRCE = 0	−0.1302	0.0174	−7.481^***^	TCE:Trial − PCRCE:Trial = 0	−0.0013	0.0011	−1.131
Trial = 0	0.0014	0.0005	2.631^†^				
**REGRESSION PATH DURATION**							
Model: lmer(ResidualLogFixDuration ~ Structure^*^Trial + (1 | Subject) + (1 | Item))							
PCRCE − NRCE = 0	0.2578	0.0248	10.404^***^	PCRCE:Trial − NRCE:Trial = 0	−0.0049	0.0011	−4.667^***^
RCE − NRCE = 0	−0.0048	0.023	−0.208	RCE:Trial − NRCE:Trial = 0	−0.0011	0.001	−1.097
TCE − NRCE = 0	−0.0431	0.023	−1.876	TCE:Trial − NRCE:Trial = 0	−0.0014	0.001	−1.440
RCE − TCE = 0	0.0383	0.0231	1.659	RCE:Trial − TCE:Trial = 0	0.0003	0.001	0.322
RCE − PCRCE = 0	−0.2626	0.0249	−10.558^***^	RCE:Trial − PCRCE:Trial = 0	0.0038	0.0011	3.575^**^
TCE − PCRCE = 0	−0.3009	0.0248	−12.152^***^	TCE:Trial − PCRCE:Trial = 0	−0.0035	0.0011	3.311^*^
Trial = 0	0.0023	0.0008	2.979^*^				
**TOTAL DURATION**							
Model: lmer(ResidualLogFixDuration ~ Structure^*^Trial + (1 | Subject) + (1 | Item))							
PCRCE − NRCE = 0	−0.2108	0.0251	−8.388^***^	PCRCE:Trial − NRCE:Trial = 0	−0.0015	0.0011	−1.351
RCE − NRCE = 0	−0.1287	0.0241	−5.336^***^	RCE:Trial − NRCE:Trial = 0	0.0020	0.0011	1.951
TCE − NRCE = 0	−0.2741	0.0242	−11.346^***^	TCE:Trial − NRCE:Trial = 0	0.0019	0.0011	1.761
RCE − TCE = 0	0.1454	0.0243	5.994^***^	RCE:Trial − TCE:Trial = 0	0.0002	0.0011	0.191
RCE − PCRCE = 0	0.0821	0.0252	3.254^*^	RCE:Trial − PCRCE:Trial = 0	0.0035	0.0011	3.243^*^
TCE − PCRCE = 0	−0.0633	0.0252	−2.509	TCE:Trial − PCRCE:Trial = 0	0.0028	0.0017	1.693
Trial = 0	−0.0008	0.0008	−1.002				

p-values: “^***^” 0.001 “^**^” 0.01 “^*^” 0.05 “†” 0.1.

*p-values adjusted for multiple comparisons using the “single-step” method in Hothorn et al. (2016)*.

*The intercept level for Structure is the rightmost Structure term in any linear hypothesis involving Structure, and is NRCE in all other cases. The intercept level for Trial is its centered mean. Trial, Frequency, and interactions involving these factors were not analyzed in cases where they did not improve model fit. These cases are indicated in the model specification and by the presence of NAs in the initial row of the interaction column*.

**Table 5:** Experiment 1 R02 (PP): Simultaneous tests for general linear hypotheses for linear effects models fitted to log-transformed and residualized fixation durations.

**Linear hypotheses**	**Est**.	**SE**	***z***	**Linear hypotheses (Interactions)**	**Est**.	**SE**	***z***
**FIRST FIXATION DURATION**							
**Model: lmer(ResidualLogFixDuration ~ Structure + Trial + (1 | Subject) + (1 | Item))**							
RCE − NRCE = 0	−0.0470	0.0137	−3.444^**^	NA	NA	NA	NA
TCE − NRCE = 0	−0.0709	0.0136	−5.206^***^				
RCE − TCE = 0	0.0238	0.0137	1.7420				
Trial = 0	0.0008	0.0003	2.855^*^				
**GAZE DURATION**							
Model: lmer(ResidualLogFixDuration ~ Structure ^*^Freq + Trial + (1 | Subject) + (1 | Item))							
RCE − NRCE = 0	−0.0467	0.0162	−2.890^*^	RCE: Freq − NRCE: Freq = 0	−0.0162	0.0072	−2.2330
TCE − NRCE = 0	−0.0541	0.0161	−3.355^**^	TCE: Freq − NRCE: Freq = 0	−0.0003	0.0071	−0.0440
RCE − TCE = 0	0.0073	0.0162	0.4530	RCE: Freq − TCE: Freq = 0	−0.0159	0.0072	−2.2060
Trial = 0	0.0012	0.0004	3.320^**^				
Freq = 0	0.0042	0.0069	0.6170				
**REGRESSION PATH DURATION**							
**Model: lmer(ResidualLogFixDuration ~ Structure + Trial + (1 | Subject) + (1 | Item))**							
RCE − NRCE = 0	−0.0040	0.0226	−0.175	NA	NA	NA	NA
TCE − NRCE = 0	−0.0413	0.0225	−1.837				
RCE − TCE = 0	0.0374	0.0226	1.653				
Trial = 0	0.0014	0.0014	2.623^*^				
**TOTAL DURATION**							
**Model: lmer(ResidualLogFixDuration ~ Structure + (1 | Subject) + (1 | Item))**							
RCE vs. NRCE = 0	−0.1287	0.0242	−5.312^***^	NA	NA	NA	NA
TCE vs. NRCE = 0	−0.2738	0.0243	−11.274^***^				
RCE vs. TCE = 0	0.1451	0.0244	5.952^***^				

p-values: “^***^” 0.001 “^**^” 0.01 “^*^” 0.05 “†” 0.1.

*p-values adjusted for multiple comparisons using the “single-step” method in Hothorn et al. (2016)*.

*The intercept level for Structure is the rightmost Structure term in any linear hypothesis involving Structure, and is NRCE in all other cases. The intercept level for Trial is its centered mean. Trial, Frequency, and interactions involving these factors were not analyzed in cases where they did not improve model fit. These cases are indicated in the model specification and by the presence of NAs in the initial row of the interaction column*.

At R01, first fixation durations displayed a significant effect of Structure for which RCE, TCE, and PCRCE all had shorter durations than the island condition, NRCE. There was also a marginal effect of Trial (*p* = 0.06) in which later trials showed longer fixation durations than early trials for all structures.

**Figure 1:**
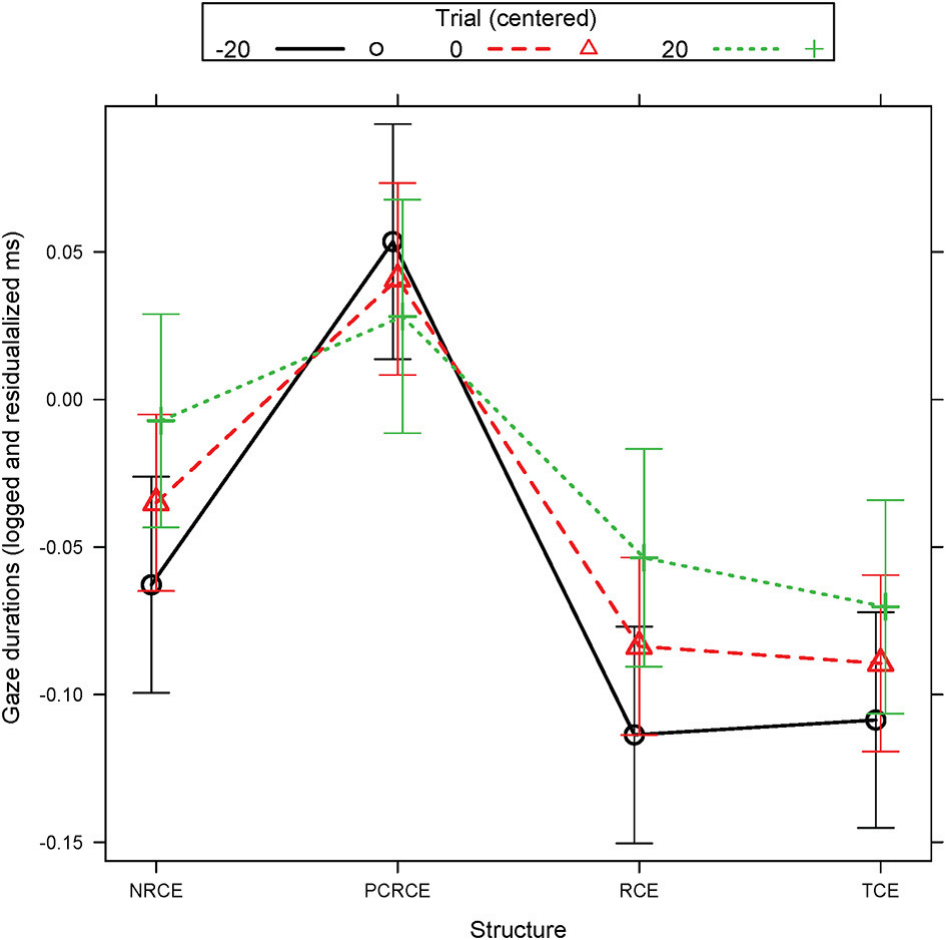
Experiment 1 R01 estimated gaze durations (logged and residualalized ms): Structure by Trial interaction. Error bars represent the 95% confidence interval (CI) of the mean.

Gaze durations showed a simple effect of Structure whereby RCE and TCE again both patterned faster than NRCE, and all three argument-bearing conditions (NRCE, TCE, and RCE) patterned faster than the intransitive PCRCE, which exhibited a significant slowdown in this measure. In addition, Structure and Trial interacted: All conditions except PCRCE showed increased cost as participants progressed through the experimental trials (Figure 1). This effect appears to be driven by RCE and (marginally) by NRCE, both of which show more cost over Trial relative to PCRCE, which remains relatively constant across trial iterations. TCE visually patterned in a similar way to RCE and NRCE, but the change over trial did not approach significance.

**Figure 2:**
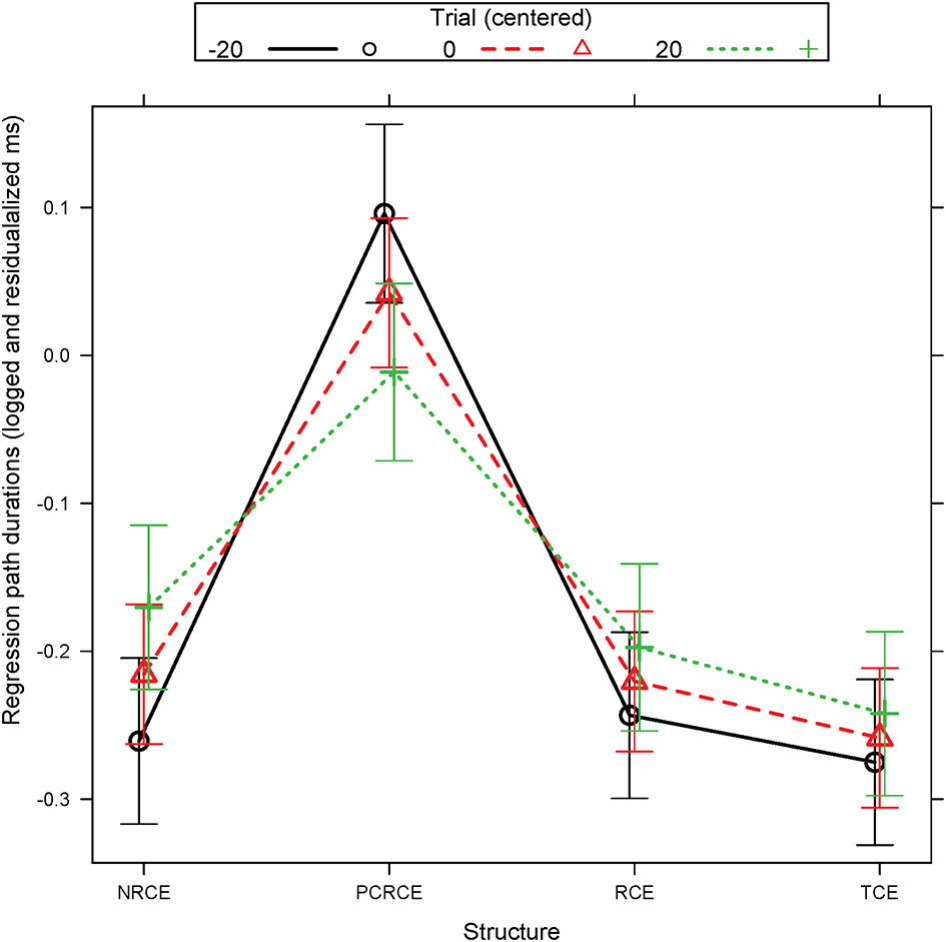
Experiment 1 R01 estimated regression path durations (logged and residualalized ms): Structure by Trial interaction. Error bars represent the 95% confidence interval (CI) of the mean.

In regression path durations, there was a simple effect of Structure for which NRCE, TCE, and RCE all showed shorter durations than the intransitive PCRCE. However, somewhat unexpectedly, NRCE did not pattern differently from either RCE or the non-island, TCE. An interaction between Structure and Trial was also present. For this, PCRCE showed increased facilitation as trial iteration increased relative to RCE, TCE, and NRCE (Figure 2). The facilitation for PCRCE however, was never such that its regression path durations approximated the other three conditions—across Trial, it was always slower, even despite the facilitation from higher trial indices. NRCE also displayed a simple effect of Trial, showing an increase in durations as Trial increased. In a follow up analysis, and from an examination of Figure 2, no such effect was Trial effect was seen for RCE (β = 0.0011, *SE* = 0.0008, *p* = 0.140) or TCE (β = 0.0008, *SE* = 0.0008, *p* = 0.278). However, this difference was not enough to produce a significant interaction between RCE, TCE, and NRCE, signaling that the differences between conditions should be interpreted with caution (Rosnow and Rosenthal, 1989; Tybout and Sternthal, 2001).

**Figure 3:**
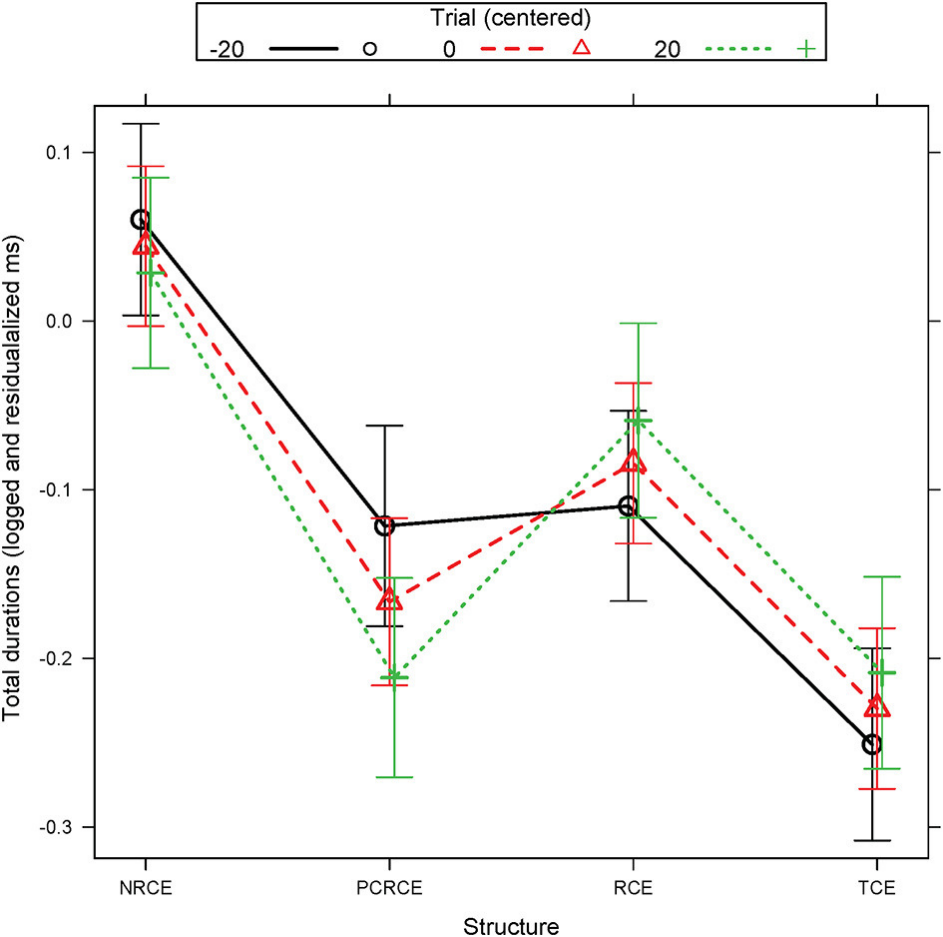
Experiment 1 R01 estimated total durations (logged and residualalized ms): Structure by Trial interaction. Error bars represent the 95% confidence interval (CI) of the mean.

In total durations, the island condition NRCE displayed longer fixation durations relative to the three other conditions. RCE showed significantly shorter durations than NRCE, but patterned longer than TCE, thus resulting in a three-way distinction between the key structures. In addition, the PCRCE control had shorter total durations than RCE and marginally longer total durations than TCE (*p* = 0.06). A Structure by Trial interaction was also present; however this effect appears to have been solely driven by the PCRCE condition, for which total fixation durations became shorter as trial iteration increased (Figure 3).

We also compared fixation times for the same four measures at the two-word PP region (R02). PCRCE was excluded from this analysis, since the two-word region that follows the first embedded verb in the PCRCE condition is not a PP but rather a conjunction plus a verb, precluding any meaningful comparisons.

At the PP, both first fixation duration and gaze duration, showed an effect of structure in which RCE and TCE had shorter fixation durations than NRCE, thus maintaining the pattern observed for both measures in R01. Trial also affected both measures: as trial index increased, fixation durations also increased. Some indications of a Structure by Freq interaction were also numerically present for R02 Gaze durations, but they were not statistically significant on account of our corrections for multiple comparisons. For this, as Freq increased, RCE's slope became more negative relative to the slopes for TCE and NRCE.

For the late measures, regression path durations failed to show any effect of Structure at R02, somewhat contrary to our expectations, but similar to what was seen at R01. There was however an effect of Trial for which increases in trial index yielded longer regression path durations. Also similar to what was seen in R01, total durations again displayed a three-way distinction between Structures, with TCE having significantly shorter total durations than RCE and NRCE and RCE having shorter durations than NRCE.

### Discussion

The presence of RCE and TCE facilitation relative to NRCE in first fixation durations, gaze duration, and total durations at the first embedded verb and the following PP region, provides initial support for the non-island status for RCE. An important question that arises is whether these differences bear any relation to actual integration and dependency formation, or if they merely represent differences in structural complexity. The first thing to note is that PCRCE patterns similarly to RCE in R01 first fixation durations. This is not surprising; regarding a locality-based metric of complexity, the two structures are highly similar. Moreover, both structures are intuitively acceptable. In addition, fixation durations were residualized and thus word length differences can be considered negligible. And yet, the two conditions contrast in that RCE has a transitive argument structure whereas the first verb encountered in the RC in PCRCE is intransitive. The similar patterning can thus be taken as an indication that integrative processes are not affecting first fixation durations for any of the structures, and that the slowdown noted for NRCE relative to the other conditions in this measure, at least at the embedded verb region, is best attributed to higher structural complexity for NRCE relative to the other conditions, and not to processes reflecting integration and dependency formation.

Facilitation for RCE (and TCE) relative to NRCE was also seen in Gaze durations at both R01 and R02. We first note that PCRCE patterned slower than all three of the other conditions. Our interpretation of this slowdown is that when the parser enters into the embedded verb region carrying the open dependency of the filler, the PCRCE intransitive light verb would be unexpected, resulting in a cost. In contrast, encountering an argument structure that provides a slot for an extracted filler, as would be present in RCE, NRCE, and TCE, would be expected (but see discussion regarding NRCE below) and would thus help to facilitate the parse relative to PCRCE. Such behavior is largely in line with findings from a similar study by Omaki et al. (2015) in which intransitive structures showed longer gaze durations than transitive structures for filler-verb integration in non-islands, a cost which they attribute to an expectation-based violation, and has further support in theories that posit a critical contribution of probabilistic information or a combination of locality and probabilistic information toward processing difficulty (Hale, 2003; Levy, 2008). Because argument structure should only affect dependency formation during integrative stages, we can infer that such processes are now contributing to the pattern of effects. Thus, the facilitation noted for RCE (and TCE) relative to NRCE can be provisionally interpreted as an indication that RCEs do not comprise a strong island violation.

To better understand the pattern of effects, we posit two costs that would arguably apply to integration and dependency formation. First, entering an island would presumably strengthen any expectation against filling the dependency inside that island, given that dependency formation is normally blocked in islands, at least when transitive/intransitive alternating verbs are used and selecting the intransitive reading is a viable parsing option (Traxler and Pickering, 1996; Omaki et al., 2015). Subsequently encountering a transitive verb (with no intransitive alternation) in that island, as in the Experiment 1 sentences, would force reanalysis or require a re-weighting of expectations at the transitive verb after an initial suspension of dependency formation upon entering the relative clause. Ultimately, there is no analysis that can save the island sentences. However, locally, within the island, we assume that some form of reanalysis or re-weighting takes place when the parser initially does not posit a gap (on account of detecting the island), but then changes course once the verb is determined to be transitive, positing that a gap must be present. This reanalysis is assumed to be associated with some cost. Second, we assume that the actual dependency formation and integration would involve an additional cost applicable to both islands and non-islands. In regard to islands, these two costs would amount to what we will refer to as a “forced integration” cost. With this in mind, now consider that RCE patterns with TCE, faster than NRCE during a measure verified to reflect integration. From this, we can surmise either that RCE's dependency formation cost is likely to be similar to TCE, even in spite of any additional complexity carried by relativization in RCE, and that RCE is not showing any obvious reanalysis cost; or that the difference is solely carried by structural complexity and there is no reanalysis cost. Given that the extra relativization in RCE is not incurring any difference from TCE, our own interpretation leans heavily toward complexity not being a significant component of the cost, which we instead attribute to reanalysis and forced integration. Thus, we posit that in this measure NRCE, but not RCE is exhibiting signs of forced integration, and that RCE is therefore not patterning similarly to a strong island violation. We acknowledge however that the other alternative remains a possibility.

First fixation durations and gaze durations in R02 also displayed Trial effects. These were manifest as increases in fixations over the course of the experiment, across all structures, including the PCRCE control. One possible explanation is that participants learn over time that the embedded verb region is critical to interpretation and thus slow down over the course of many trials when they encounter the verb. There was also an interaction between Structure and Trial that was present in gaze durations for R01. For this, all conditions except PCRCE showed facilitation for higher trial iteration at the first embedded verb. Similar to our earlier conclusions, we assume that this finding relates to argument structure. Over the course of the experiment, more evidence is accrued supporting the possible presence of an intransitive verb in the RC, and participants learn that the filler-verb relation matters more for argument bearing conditions (even when in an island–as with NRCE) than non-argument bearing conditions like PCRCE. The shift to shorter durations for PCRCE would then represent the parser re-weighting predictions such that the presence of an intransitive light verb no longer comprises a significant anomaly.

For regression path durations in both regions, no distinction between NRCE, TCE, and RCE was present. However, the Trial by Structure interaction at the embedded verb whereby PCRCE was facilitated as Trial increased relative to RCE, TCE, and NRCE, coupled with a cost over increase in Trial for NRCE, supports our earlier conclusion that some form of forced integration may be occurring in NRCEs, whereby the availability of only a single potential gap position for filler integration within the RC becomes increasingly salient to participants over the course of the experiment. As participants learn that no additional gap is forthcoming, they then increasingly attempt to fill the existing gap in the NRCE condition. Critically, this does not occur for the intransitive PCRCEs, which cannot project such a gap and thus instead become increasingly facilitated as participants' expectations change over trial, and they begin to shift attention away from the intransitive light verb. It also does not appear to occur for TCE or RCE conditions, again suggesting that RCE is not a strong island violation.

In total durations at both the first embedded verb and at the following PP, a three-way distinction emerged whereby TCE patterned faster than RCE and NRCE, and RCE patterned faster than NRCE. Total durations comprise more a cumulative, less-online, reflection of overall complexity and non-local, sentential cohesion. We thus take the longer durations for RCE relative to TCE to represent a late cost of the extra filler-gap dependency that relativization involves. Given that NRCE islands, are the most complex of our structures, it is not surprising that their durations were longer than in all other conditions for this measure.

Experiment 1 thus provides initial evidence that RCEs are not processed as a strong island violation. However, the possibility that integration was surprisingly widespread, even appearing on NRCE, the known island condition, stands in contrast to findings from related studies on English RCs in which the parser has been shown to suspend dependency formation inside syntactic islands (Traxler and Pickering, 1996; Phillips, 2006; Omaki and Schulz, 2011; Omaki et al., 2015). One possible explanation is that the single-gap design promotes some form of integration on account of the global ungrammaticality that arises from the absence of a licit gap position when an island is present. Such integration would not necessarily represent syntactic integration. For example, because all of the filler-gap relations in our stimuli are plausible, the parser may be able to establish a “superficial” filler-verb relation in spite of any difficulty in processing the actual syntactic structure. Boland's (1997) concurrent theory, though originally developed in reference to ambiguity resolution, provides one means by which this could occur. In this approach a semantically-based provisional interpretation may be developed rapidly during processing on the basis of verb argument structure and lexical-semantics even when structural analysis is incomplete, allowing a semantic route to be established before a syntactic route has been resolved. If this is correct, the parser should then still be able to accomplish some level of integration even when it enters an island. Additional support for partial processing in which both syntax and semantics are relevant, but the syntactic form can be imprecise to some degree is found in the “good-enough” approach to sentence processing (Ferreira, 2003; Ferreira and Patson, 2007; Lim and Christianson, 2013; Karimi and Ferreira, 2016).

Another explanation stems from the absence of a plausibility manipulation in Experiment 1. Previous studies rely on an interaction between Plausibility/semantic congruity and Structure to illustrate when integration is attempted. The presence of plausible and implausible fillers in these designs could require fundamentally different experimental task demands from what was used in our Experiment 1. For example, the plausibility manipulation could heighten awareness of the filler-verb dependency across trials, and as a consequence draw increased attention to the actual structure that the dependency extends into, thereby also drawing more attention to the presence of a syntactic island.

We have provided extensive argumentation that Experiment 1 provides evidence for the non-island status of RCE; and we continue to favor that conclusion. However, it is also the case that each of these two explanations could conceivably be extended to argue that the facilitation for RCE noted in Experiment 1 is not unequivocally due to non-islandhood. To address this, Experiment 2 adopts a semantic plausibility manipulation and a two-gap design to reduce the potential for forced integration and to allow a closer comparison to previous studies on English RCEs.

## Experiment 2

In Experiment 2 we again use an eyetracking while reading paradigm to investigate the processing of RCEs, but enact two key changes to our materials to address aspects of our experimental design that may have engendered forced integration in the NRCE condition and which prevented us from unequivocally drawing conclusions regarding RCE in Experiment 1. First, to reduce the risk of forced integration, we implement a dual-gap design with a filler-verb plausibility manipulation similar to that used in Traxler and Pickering (1996) and Omaki et al. (2015). This also allows for a greater degree of comparison to previous studies, in that we can now focus on comparisons within a particular structure and match those studies in terms of task demands. Second, we include a within-structure plausibility manipulation to remove the necessity of using maximally similar structures and to allow us to use another type of strong island, extractions from subjects (SRCE). SRCE has traditionally been considered to comprise a strong island violation, the Subject Condition (Huang, 1982; Chomsky, 1986), and unlike RCE, this violation has been observed to be respected in Swedish (Engdahl, 1982). In addition, SRCE provides an island structure that involves extraction from an RC, and is thus consistent in terms of relativization compared to RCEs, but is not dependent on the presence of a specific type of adverb to induce degradation, as was the case with NRCE.

Our design thus includes two Structure variants: RCE (6c, 6d) and SRCE (6a, 6b) and a plausibility manipulation (Plausibility). For this, each sentence began with a filler-NP headed by a concrete noun which functioned as either a semantically/pragmatically congruent filler (Plausible) to the embedded RC verb (möbler/“furniture”, as in 6a for SRCE; 6c for RCE) or as an incongruent filler (Implausible) (flyttlådor/“moving boxes”) to the verb (6b for SRCE; 6d for RCE).

(6)   a. SRCE Plausible
            Såna där möbler   bad    en kollega     som renoverade
            Such        furniture  asked a   colleague   who renovated
            [–] på landet            mig
            [–] at countryside-the me
*            att bära [–] efter matchen   i     söndags*.
            to   carry [–] after match-the   last Sunday.
            “Such furniture, a colleague who renovated at the countryside asked me to carry after the match last Sunday.”
        b. SRCE Implausible
           Såna där flyttlådor           bad   en kollega    som
           Such       cardboard-boxes asked a  colleague who
           *renoverade [–] på landet                mig*
           renovated   [–]   at  countryside-the   me
*           att bära   [–] efter matchen     i     söndags*.
           to carry    [–] after match-the     last  Sunday.
           “Such cardboard boxes, a colleague who renovated at the countryside asked me to carry after the match last Sunday.”
         c. RCE Plausible
           Såna där möbler    bad    jag en kollega     som
           Such       furniture   asked I      a    colleague that
           *renoverade [–] på landet*
           renovated    [–] at   countryside-the
*           att bära [–] efter matchen    i      söndags*.
           to  carry  [–] after  match-the   last  Sunday.
           “Such furniture, I asked a colleague who renovated at the countryside to carry after the match last Sunday.”
         d. RCE Implausible
           Såna där flyttlådor           bad    jag en kollega    som
           Such       cardboard-boxes asked I    a    colleague that
           *renoverade [–] på landet*
           renovated    [–]  at   countryside-the
*           att bära [–] efter matchen    i     söndags*.
           to   carry [–] after match-the    last Sunday.
           “Such cardboard boxes, I asked a colleague who renovated at the countryside to carry after the match last Sunday.”

One tradeoff to enlisting a dual-gap design is that the presence of a second gap potentially opens the door for parasitic gapping (PG), a phenomenon whereby an illicit gap is rescued in the presence of an additional licit gap position in the sentence, and both gaps are linked to the same filler phrase (Ross, 1967; Culicover, 2001; Phillips, 2006). Parasitic gapping inside complex subjects has been claimed to be possible in Swedish (Engdahl, 1983), as well as in English. The illicit gap in (7a) (the subject island violation) can be rescued by being parasitically linked to a licit gap, thus rendering the sentence acceptable, cf. (7b) (example sentences borrowed from Phillips, 2006 p. 802).

(7)  a. ^*^What did the attempt to repair [–] ultimately damage the car?
       b. *What did the attempt to repair [_*PG*_ –] ultimately damage [–]*?

Unlike English, Swedish is claimed to allow parasitic gapping in finite clauses to some degree (Engdahl, 1983). It is consequently more difficult to preclude the occurrence of parasitic gapping in our sentences than it would be, for example, in an experiment using English language materials that was also based on the general Traxler and Pickering (1996) design.

However, there are two reasons why we are optimistic that the threat of parasitic gapping will be minimal. First, relativization in combination with tense (as in our RCs) appears at the lowest end of the hierarchy of accessible parasitic gap domains, as proposed in Engdahl (1983). Second, our use of a PP (på landet/“at the countryside”) between the two gap positions could help to strengthen the parser's commitment to an intransitive analysis of the embedded RC verb and thus diminish the potential for parasitic gapping (see Chaves, 2013). Despite this, participants may become aware of the dual-gaps over the course of the experiment, thus potentially engendering parasitic gapping. To address this, we model the effect of trial in each of our analyses. This allows us to monitor parasitic gapping to the extent that it may arise from a learning effect over the course of the experiment.

A second tradeoff is that the number of dependents crossed is no longer equal; RCEs cross an extra dependency relative to SRCEs (*jag*/“I”) at the first gap. However, this imbalance is critically biased against RCEs integrating more successfully than SRCEs.

If RCEs are licit, non-island structures, as Experiment 1 suggests, and if the dual-gap design reduces the potential of forced integration at the embedded RC verb region (R01) and the following PP region (R02) without introducing parasitic gapping, then interactions should be present in early and late measures whereby RCEs, but not SRCEs (on account of their island status) show facilitation for Plausible relative to Implausible at R01/R02.

### Method

#### Participants

Sixty-six monolingual, native Swedish speakers participated in the study in exchange for a cinema ticket. All participants were aged 18–40 and were screened to not have native-like levels in any other language. Six participants were removed from the study for scoring below 75% on the comprehension questions, thus leaving 60 participants for analysis.

#### Materials

Twenty-eight critical items, each appearing in two Structure variants (RCE and SRCE) and two Plausibility variants (Plausible and Implausible), as in (4) were constructed for the experiment. A full list of the critical items can be found in the Supplementary Material for this paper. The critical items were rotated together with 32 distractor items over four experimental lists in a Latin Square design and then randomized. Each participant was assigned to one experimental list and thus only ever saw one version of each sentential item.

For sentence construction, all regions except the final wrap-up region had an equivalent number of words and were highly similar in constituent class and features, both within and between items. To structurally accommodate the dual-gap, all matrix verbs now comprise a ditransitive verb (*bad*/“asked”) that licenses a Subject NP, an NP object, and a clausal object, with the NP object slot providing the first potential extraction site. In addition to the matrix verb modification, the embedded RC verb is now always an alternating transitive/intransitive verb (*renoverade*/“renovated”) and serves to introduce the first potential gap for filler-gap integration. On account of the Structure manipulation, this gap appears in the RC of one of two structures: a topic-extracted object NP (as with RCEs) or a topic-extracted subject NP (as with SRCEs). The second gap position is provided by the clausal object of the ditransitive verb, which contains its own transitive verb (*att bära*/“to carry”). It is this gap that ultimately functions as the “true” gap for the extracted filler in each sentence.

For verb-filler selection, we collected ratings for 32 sets of sentences (7) containing semantically congruent (Plausible: 7a, 7c) and incongruent (Implausible: 7b, 7d) embedded RC verb (7a, 7b) and Clausal Object verb (7c, 7d) verb-filler relations. Items were rotated across two lists, with each participant assigned to one list such that they would only ever see one RC verb and one Object verb variant of each item, with congruency alternating between the two. Sixteen ungrammatical and 16 grammatical distractor items involving RCs were randomly interspersed among the test items, as were 21 yes/no comprehension questions. Ratings were collected in two stages, allowing us to modulate some items to better match our selection criteria. The first stage had 23 participants and the second had 25 participants.

(7)   a. RC-verb Plausible
           Hon tränade   såna komplicerade volter           nästan
           she   practiced such  complicated     somersaults   almost
*           dagligen*.
           daily.
       b. RC-verb Implausible
           Hon tränade   såna komplicerade pajer nästan dagligen.
           she   practiced such  complicated    pies    almost  daily.
           “She practiced such complicated {somersaults/pies} almost daily.”
       c. Clausal-Object verb Plausible
*           Hon gjorde  såna komplicerade volter          på  försök*.
           she   made   such  complicated     somersaults on  attempt.
       d. Clausal-Object verb Implausible
*           Hon gjorde såna komplicerade pajer på försök*.
           she   made   such complicated     pies   on attempt.
           “She attempted to do such complicated {somersaults/pies}.”

Verb-filler relations for verbs to be used as embedded RC verbs were coded as Implausible if their mean rating of ≤2.7, and were coded as Plausible if their mean rating was ≥5.5. Verbs were only considered for use if the filler-verb pairing for the clausal object verb had ratings ≥5.5.

#### Procedure

The experiment procedure was identical to that found in Experiments 1.

### Data analysis

Prior to analysis, an automatic procedure incorporated fixations <80 ms into larger fixations within a one-character range and then deleted fixations of <40 ms within three characters of any other fixation, and single fixations exceeding 800 ms were removed. The remaining values were log-transformed to correct for positive skewness.

We used linear mixed models to analyze fixations for four measures: first fixation duration, gaze duration, regression path duration, and total durations, at R01, the embedded verb (*renoverade*/“renovated”), and R02, the following prepositional phrase (*på landet*/“at the countryside”). To fit our models, we began with a “beyond optimal model” (Zuur et al., 2009) which included two categorical predictors, Structure and Plausibility (plus their interaction terms), and one centered, continuous variable, Trial, as fixed factors, as well as a random effect structure that included intercepts for subject and item, and by-item and by-subject slopes for Structure. The random component was reduced in a stepwise fashion until it reached convergence. We then fit the fixed component using the same procedure outlined in Experiment 1, with the exception that in the fixed effects structure, we compared log likelihood between models until we identified the best-fit model that now minimally included the Structure and Plausibility interaction terms, as these factors are critical to our hypotheses in Experiment 2. A set of contrasts was constructed using the glht function in the multcomp package (Hothorn et al., 2016).

### Results

We first present eyetracking measures for the embedded verb region (R01) and then for the following PP region (R02). Raw means are presented in Table 6. Statistical models and the results of the mixed models analyses can be found in Tables 7, 8.

**Table 6:** Experiment 2 mean and residualized fixation durations for Structure and Plausibility.

**Measure and structure**	**Plausibility**	**Region 01**		**Region 02**	
		**Mean**	**(SE)**	**Mean**	**(SE)**
**FIRST FIXATION DURATION**					
RCE	Implausible	232.35	−5.5	240.69	−5.2
	Plausible	223.92	−5.7	245.12	−5.7
SRCE	Implausible	233.41	−5.9	245.58	−5.6
	Plausible	230.55	−5.8	239.18	−5.2
**GAZE DURATION**					
RCE	Implausible	289.19	−9.3	409.83	−10.1
	Plausible	261.08	−8	400.93	−9.4
SRCE	Implausible	289.56	−8.8	416.26	−11.1
	Plausible	275.9	−8.3	400.67	−10
**REGRESSION PATH DURATION**					
RCE	Implausible	416.1	−20.6	561.33	−25.3
	Plausible	385.07	−20.9	528.45	−22.3
SRCE	Implausible	421.82	−20.8	607.07	−25.4
	Plausible	449.82	−25.2	551.21	−20.7
**TOTAL DURATION**					
RCE	Implausible	549.39	−19.2	761.95	−24.1
	Plausible	477.65	−15.6	719.01	−21.2
SRCE	Implausible	599.63	−20.6	872.95	−27
	Plausible	552.26	−18	810.19	−22.6

**Table 7:** Experiment 2 R01 (Embedded verb): Simultaneous tests for general linear hypotheses for linear effects models fitted to log-transformed and residualized fixation durations.

**Linear hypotheses**	**Est.**	**SE**	***z***	**Linear hypotheses (Interactions)**	**Est.**	**SE**	***z***
**FIRST FIXATION DURATION**							
Model: lmer(ResidualLogFixDuration ~ Structure ^*^ Plausibility + (1 | Subject) + (1 | Item))							
RCE − SRCE = 0 (Implaus intercept)	0.0015	0.0234	0.066	RCE:Plaus = 0	−0.0338	0.0332	−1.020
RCE − SRCE = 0 (Plaus intercept)	−0.0323	0.0235	−1.372				
Plaus − Implaus = 0 (SRCE intercept)	−0.0087	0.0233	−0.372				
Plaus − Implaus = 0 (RCE intercept)	−0.042503	0.0236	−1.802^†^				
**GAZE DURATION**							
Model: lmer(ResidualLogFixDuration ~ Structure ^*^ Plausibility^*^Trial + (1 | Subject) + (1 | Item))							
RCE − SRCE = 0 (Implaus intercept)	−0.0067	0.0303	−0.222	RCE:Plaus = 0	−0.0494	0.0429	−1.15
RCE − SRCE = 0 (Plaus intercept)	−0.0561	0.0305	−1.842^†^	RCE:Trial = 0	−0.0091	0.0039	−2.313^*^
Plaus − Implaus = 0 (SRCE intercept)	−0.0365	0.0302	−1.208	Plaus:Trial = 0	−0.0043	0.0039	−1.100
Plaus − Implaus = 0 (RCE intercept)	−0.085865	0.0305	−2.813^**^	RCE:Plaus:Trial = 0	0.0111	0.0054	2.051^*^
Trial = 0	0.0045	0.0028	1.612				
**REGRESSION PATH DURATION**							
Model: lmer(ResidualLogFixDuration ~ Structure ^*^ Plausibility + Trial + (1 | Subject) + (1 | Item))							
RCE − SRCE = 0 (Implaus intercept)	−0.0242	0.0427	−0.567	RCE:Plaus = 0	−0.0829	0.0605	−1.369
RCE − SRCE = 0 (Plaus intercept)	−0.1071	0.043	−2.493^*^	Plaus:Trial = 0	−0.0043	0.0039	−1.100
Plaus − Implaus = 0 (SRCE intercept)	0.0193	0.0426	0.453				
Plaus − Implaus = 0 (RCE intercept)	−0.063585	0.043	−1.477				
Trial = 0	−0.0040	0.0019	−2.127^*^				
**TOTAL DURATION**							
Model: lmer(ResidualLogFixDuration ~ Structure ^*^ Plausibility + Trial + (1 | Subject) + (1 | Item))							
RCE − SRCE = 0 (Implaus intercept)	−0.0842	0.0407	−2.07^*^	RCE:Plaus = 0	−0.0526	0.0575	−0.914
RCE − SRCE = 0 (Plaus intercept)	−0.1368	0.0407	−3.358^***^	Plaus:Trial = 0	−0.0043	0.0039	−1.100
Plaus − Implaus = 0 (SRCE intercept)	−0.0775	0.0407	−1.904^†^				
Plaus − Implaus = 0 (RCE intercept)	−0.130064	0.0407	−3.198^**^				
Trial = 0	−0.0044	0.0018	−2.428^*^				

p-values: “^***^” 0.001 “^**^” 0.01 “^*^” 0.05 “†” 0.1.

*Intercept levels for Structure and Plausibility are noted in the table. The intercept for Trial is its centered mean*.

*Simple effects of Trial were only analyzed in regard to intercept: Structure = SRCE and Plausibility = Implaus, as our primary interest was in regard to the interaction terms. Trial was not analyzed when it did not improve model fit*.

**Table 8:** Experiment 2 R02 (PP): Simultaneous tests for general linear hypotheses for linear effects models fitted to log-transformed and residualized fixation durations.

**Linear hypotheses**	**Est.**	**SE**	***z***	**Linear hypotheses (Interactions)**	**Est.**	**SE**	***z***
**FIRST FIXATION DURATION**							
Model: lmer(ResidualLogFixDuration ~ Structure ^*^ Plausibility + (1 | Subject) + (1 | Item))							
RCE − SRCE = 0 (Implaus intercept)	−0.01438	0.022	−0.655	RCE:Plaus = 0	0.04161	0.0311	1.338
RCE − SRCE = 0 (Plaus intercept)	0.02723	0.022	1.236				
Plaus − Implaus = 0 (SRCE intercept)	−0.02728	0.022	−1.243				
Plaus − Implaus = 0 (RCE intercept)	0.0143	0.022	0.651				
**GAZE DURATION**							
Model: lmer(ResidualLogFixDuration ~ Structure ^*^ Plausibility + (1 | Subject) + (1 | Item))							
RCE − SRCE = 0 (Implaus intercept)	−0.01076	0.0309	−0.348	RCE:Plaus = 0	0.02889	0.0438	0.66
RCE − SRCE = 0 (Plaus intercept)	0.01813	0.031	0.585				
Plaus − Implaus = 0 (SRCE intercept)	−0.03008	0.0309	−0.974				
Plaus − Implaus = 0 (RCE intercept)	−0.001191	0.031	−0.038				
**REGRESSION PATH DURATION**							
Model: lmer(ResidualLogFixDuration ~ Structure ^*^ Plausibility + Trial + (1 | Subject) + (1 | Item))							
RCE − SRCE = 0 (Implaus intercept)	−0.068685	0.0369	−1.862^†^	RCE:Plaus = 0	0.02242	0.0523	0.429
RCE − SRCE = 0 (Plaus intercept)	−0.0463	0.037	−1.250				
Plaus − Implaus = 0 (SRCE intercept)	−0.067391	0.0369	−1.827^†^				
Plaus − Implaus = 0 (RCE intercept)	−0.044971	0.037	−1.215				
Trial = 0	−0.00408	0.0016	−2.502^*^				
**TOTAL DURATION**							
Model: lmer(ResidualLogFixDuration ~ Structure ^*^ Plausibility + Trial + (1 | Subject) + (1 + Structure | Item))							
RCE − SRCE = 0 (Implaus intercept)	−0.108551	0.0376	−2.885^**^	RCE:Plaus = 0	−0.009475	0.0488	−0.194
RCE − SRCE = 0 (Plaus intercept)	−0.1180	0.0376	−3.135^**^				
Plaus − Implaus = 0 (SRCE intercept)	−0.049131	0.0345	−1.425				
Plaus − Implaus = 0 (RCE intercept)	−0.058606	0.0345	−1.697^†^				
Trial = 0	−0.004743	0.0015	−3.111^**^				

p-values: “^***^” 0.001 “^**^” 0.01 “^*^” 0.05 “†” 0.1.

*Intercept levels for Structure and Plausibility are noted in the table. The intercept for Trial is its centered mean*.

*Simple effects of Trial were only analyzed in regard to intercept: Structure = SRCE and Plausibility = Implaus, as our primary interest was in regard to the interaction terms. Trial was not analyzed when it did not improve model fit*.

First fixation durations in R01 showed a marginal simple effect of Plausibility for RCE in which the Plausible pairings produced shorter fixations than the Implausible pairings (*p* = 0.07). No such Plausibility effect was seen for SRCE. However, despite this difference in Plausibility effects, there was no significant interaction, signaling that the differences between conditions should be interpreted with caution (Rosnow and Rosenthal, 1989; Tybout and Sternthal, 2001).

**Figure 4:**
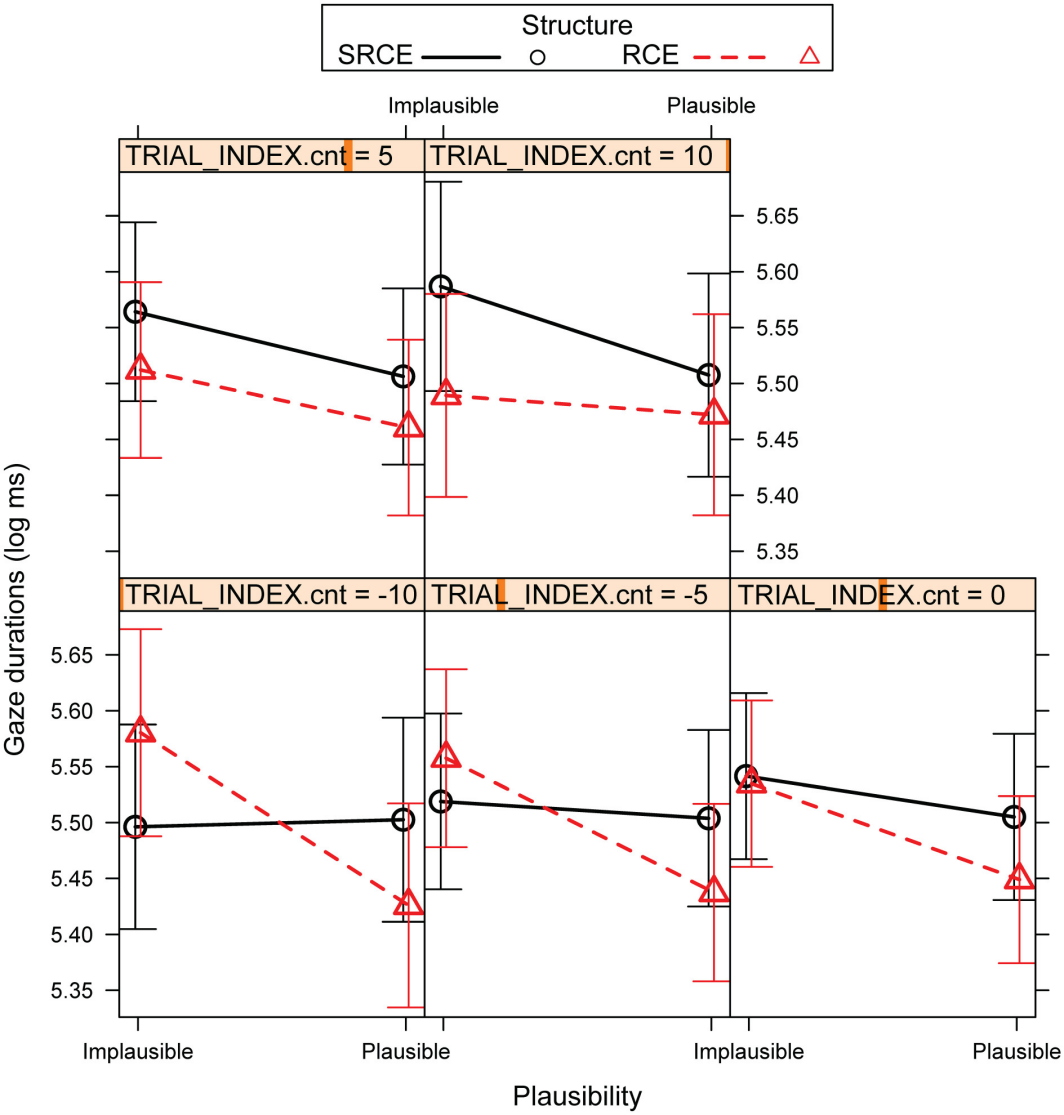
Experiment 2 R01 estimated gaze durations (log ms): Structure by Plausibility by Trial interaction. Error bars represent the 95% confidence interval (CI) of the mean.

Gaze durations for the same region showed a marginally significant simple effect of Structure for which RCE patterned faster than SRCE in the Plausible conditions (*p* = 0.07). There was also a simple effect of Plausibility for RCE in which the Plausible pairings had shorter durations than Implausible pairings. In addition, there was a significant three-way Structure by Plausibility by Trial interaction in which the slope for RCE showed a greater negativity in the change from Implausible to Plausible than the slope for SRCE, but only in early trials; such facilitation in was lost in late trials (Figure 4). That is, in early trials, RCEs showed signs of sensitivity to the Plausibility manipulation in the form of a cost for the Implausible pairings, whereas SRCEs, a clear case of islandhood, did not, as would be expected if RCEs are non-islands. However, this distinction for RCE was lost in later trials.

For the later measures in R01, regression path durations showed a Structure effect in which RCE was facilitated relative to SRCE for Plausible filler-verb pairings. Although this effect was not present in Implausible pairings, the Structure by Plausibility interaction was not significant, precluding a strong conclusion regarding how the two structures might show different sensitivity to the Plausibility manipulation, though the pattern is still suggestive of one. Total durations also showed an effect of Structure whereby RCEs had shorter durations than SRCEs for both Plausible and Implausible conditions. In addition, RCE showed an effect of Plausibility whereby Plausible conditions had shorter durations than Implausible conditions. SRCE, patterned similarly, but the effect was marginal (*p* = 0.06). Both regression path durations and total durations showed a simple effect of Trial for Implausible SRCEs. As trial index increased, fixation durations decreased. We did not analyze simple effects of Trial for other intercepts, as our primary interest lay in the interactions, which were not significant.

At R02, the following PP region, no effects were found for the two early measures. In the late measures, regression path durations showed a marginal effect of Structure, whereby RCE produced shorter durations than SRCEs in the Implausible condition (*p* = 0.06). However, the interaction between Structure and Plausibility did not reach significance. There was also a marginal effect of Plausibility for which Plausible pairings had shorter durations than Implausible pairings for the SRCE condition (*p* = 0.07). Again, the interaction was not significant. In total durations, RCE now showed a significant effect of Structure, displaying shorter durations than SRCEs, this time in both Plausible and Implausible conditions. There was also a marginal effect of Plausibility for which Plausible pairings had shorter durations than Implausible pairings for the RCE condition (*p* = 0.09), again with no significant interaction. Finally, both regression path duration and total duration showed significant simple effects of Trial for Implausible SRCEs in which later trials yielded shorter durations than earlier trials.

### Discussion

In Experiment 2 we tested the island status of Swedish RCEs by comparing their sensitivity to a semantic plausibility manipulation relative to SRCEs, which being strong-islands, should block filler-verb dependency formation and thus avoid any cost related to implausible filler-verb relations, as has been demonstrated by Traxler and Pickering (1996) and Omaki et al. (2015) for English relative clause extractions. Experiment 2 also tests Structural facilitation for RCEs relative to a strong island. However, this comparison requires extraction over not only potentially different underlying structure, but also over different overt discourse referents; RCEs cross an extra dependency relative to SRCEs (jag/“I”) at the first gap. Although this difference exists, the imbalance is critically biased against RCEs integrating more successfully than SRCEs.

Across most measures, the Plausibility manipulation affected primarily RCE, eliciting longer fixation durations in the Implausible conditions than in the Plausible conditions. This was true for first fixation durations, gaze durations, and total durations at the embedded verb and for total durations at the following PP. In general, SRCE did not display such differences, which is suggestive of an SRCE/RCE distinction, though the interactions themselves did not reach significance. SRCE did however display a marginally significant cost for Implausible pairings in the simple effects for two measures: total durations at the embedded verb and regression path durations at the PP. This suggests that the island condition is not effectively blocking integration and dependency formation in later stages of the parse. We take this to represent a possible consequence of parasitic gapping—the ditransitivity of the matrix verb may have acted as a cue supporting the possibility of an upcoming second gap, which in turn would support the parser's postulation of a parasitic gap in the RC, thus allowing semantic integration to occur even in SRCE. If parasitic gapping is indeed occurring, there are no indications that it is also affecting RCE, since RCE shows additional Plausibility effects in other measures. Thus, the potential presence of parasitic gapping on SRCE only further demonstrates that RCE is not patterning similarly to the strong island in terms of dependency formation and integration.

Another key finding was that in gaze durations at the embedded RC verb, RCEs with Plausible filler-verb pairings were facilitated relative to their Implausible counterparts, provided that trial indices were low. In comparison, the Plausibility manipulation had no significant effect on SRCEs. The processing pattern observed for the early trials is thus in-line with the predicted interaction whereby only non-islands should show sensitivity to the plausibility of the filler-verb relation, providing some initial support for the non-island status of RCEs. However, the slope of the RCE Plausibility effect became less negative over Trial increases, driven by increasing facilitation for the Implausible condition. One possibility is that participants learned over time that mismatched filler-verb pairings at the RC verb do not need to be immediately resolved, and that doing so may even be detrimental to the parse, especially if they come to anticipate the presence of a later potential gap site. Thus, participants may have learned to delay commitment to mismatched material in this region and consequently attributed less attention to immediately resolving semantically incongruent filler-verb pairings. There may still be some cost to the detected anomaly, but it would be masked in comparison to the Plausible conditions on account of the cost of integration that we expect affects the Plausible pairings across Structure and Trial. Thus, though the Implausible pairings appear to approximate the Plausible ones, the latter are already showing some baseline of cost on account of performing normal integration. That the gaze durations are relatively stable for the Plausible pairings for both Structures supports just such a conclusion. Alternatively, it may simply be that participants learn to ignore the potentially intransitive verb over time.

In addition to the Plausibility effects, Structural facilitation was visible across a number of measures for RCE relative to the SRCE strong island, including gaze duration, regression path duration, and total durations at the embedded verb, and in regression path durations and total durations at the following PP. Furthermore, this facilitation was seen in spite of the greater number of crossed discourse referents in RCE. Taken together, the Plausibility and Structure effects thus suggest that RCE does not involve a strong island violation.

## General discussion

The current study is the first of its kind to investigate the processing of relative clause extractions in Swedish. In Experiment 1, we directly compared the processing pattern for RCEs to that of a strong island violation (NRCE), as well to licit extractions from *that*-clauses (TCE) and an intransitive control (PCRCE) condition. RCEs were seen to pattern differently from NRCE strong islands and were often processed in a similar way to licit *that*-clause extractions (TCE), despite RCEs being more similar to the island conditions in terms of relativization. In Experiment 2, we manipulated the plausibility of filler-verb pairings for RCEs and another type of strong island violation (SRCE), similar to the approach taken by Traxler and Pickering (1996), allowing us to monitor for signs of semantic integration independently within each structure. Integration-based processing differences were observed between RCEs and SRCE strong islands, present both as simple effects of Plausibility and Structure across eyetracking measures, and as differences in potential learning effects over increases in trial as participants proceeded through the experiment. Structural facilitation was also observed for RCE relative to SRCE despite any cost for crossing an additional discourse referent in the RCE structures. Taken together, the results from Experiments 1 and 2 suggest that Swedish RCEs are processed differently than at least two types of strong island structures, and therefore are not likely to comprise a case of strong island violation. Our findings thus provide a processing correlate to the intuitive acceptability of RCEs, as reported in the literature.

However, it was not clear from our findings that Swedish RCEs should be treated as non-islands, on par with TCEs. In total durations (Experiment 1), a measure of the overall cohesive success of the sentence, RCEs were seen to pattern in-between non-islands and islands, which echoes their below-midpoint ratings presented herein and in Müller (2015). This difference may simply reflect a late cost of relativization, but it could also signal that RCEs are more island-like than TCEs in the sense that integration is associated with more cost in these structures. We first explore the latter possibility, and then discuss the implications of our findings in terms of deep vs. surface variation.

In a recent proposal, Lindahl (2015) treats RCEs as a type of licit *weak island extraction* (WIE). Descriptively speaking, *weak* islands differ from *strong* islands in allowing certain extractions to occur from the island, while banning others (Rizzi, 1990). A paradigmatic case is *wh*-islands, (8) (from den Dikken and Szabolcsi, 2002 p. 219), which marginally allow argument extraction but not adjunct extraction.

(8)  a.  ?*Which man are you wondering whether to invite [–]?*
       b.  ^*^How are you wondering whether to behave [–]?

A WIE approach has in fact also been made to explain the acceptability (for some speakers) of extractions out of indefinite NPs with a relative clause in English (Postal, 1998). A challenge to the application of a WIE analysis for Swedish RCEs has been that many of the suggested restrictions on RCEs (including those relating to the head noun, the matrix predicate, the matrix subject, and the position of the relative clause) either do not hold up to scrutiny or are derivable from independent factors (Heinat and Wiklund, 2015). Moreover, Swedish seems to differ from the English varieties analyzed in Postal (1998) in allowing extraction even from definite DPs and in allowing PP-/adjunct extraction from the RC (Heinat and Wiklund, 2015). Despite this exceptionally liberal behavior of Swedish RCEs, Lindahl (2017) identifies at least one restriction that applies to them. She shows that certain *wh*-adjuncts (*varför*/“why”) cannot be extracted from the relative clause (9), and in doing so provides an argument that Swedish RCEs, while not strong islands, are weak islands in the sense presented above.

(9) ^*^Varför_i_ känner du många som blev sena till festen_i_?
       Why      know   you many  who were late   to party-the
      “Why do you know many people who were late the the party?”

A WIE approach to RCEs is compatible with the results in the current study, in that we would expect licit WIEs to be processed either on a par with our non-island condition, or between the non-island and the islands condition if for example the cost for relativization is high enough to influence the pattern of results, or the absence of context affects weak islands more than non-islands. The weak island approach, but less clearly the non-island approach, also appears compatible with the somewhat low ratings that RCEs receive in formal experiment situations, as WIEs are expected to require contextual conditioning which is often absent (for practical reasons) from acceptability experiments. Successful WIEs have often been claimed to be conditioned on specific readings in relation to the filler, for example, an existential presupposition reading (den Dikken and Szabolcsi, 2002; Boeckx, 2012). This suggests that contextual conditioning is very important and is a factor which should receive more attention when studying the processing of RCEs.

Turning to variation in island effects and the concomitant question of whether true (deep) variation in island constraints exists to explain the former variation, our findings do not preclude any conclusion in support of deep variation. However, if Swedish RCEs involve licit weak island extractions, as we have suggested above, then these structures instead appear to conform to the proposed universality of island constraints, proven to be quite robust despite a few exceptions. On this account, Complex NPs with relative clauses (indefinite and definite) *are* islands in Swedish, like in English, only much weaker islands than their English counterparts, the latter either strong islands (standardly) or weak islands (in the varieties reported on in Postal 1998).

Insofar as we understand Phillips' (2013) characterization of variation in relation to island effects, any variation cast in terms of island strength must derive from *surface* variation (structure) rather than *deep* variation in the island constraints themselves. Quite standardly, a non-island domain (to the extent that this extreme exists) is a structure simple enough to allow all extraction. A strong island domain (to the extent that this extreme exists) is a structure complex enough to ban all extraction. Different types of weak island domains fall in between these two extremes but they share the property of allowing some and banning other extractions. Which extractions are allowed, depends on the (intervening) features/structures that create the island (Starke, 2001; den Dikken and Szabolcsi, 2002; Boeckx, 2012).

If relative clauses are weak islands, and the set of admissible extractions varies between languages as a function of the features/structures present in the relative clause, then we need to identify what features of the relative clause comprise the locus of variation and the different structures that these features project to create the variation in evidence. The relevant features need not necessarily relate solely to relativization. One possibility is that relativization in combination with finiteness is what induces island effects in relative clauses (generally across languages), but that features relating to finiteness comprise a locus for variation in regard to RCE. The latter receives support from a recent study on extraction from adjunct islands by Müller (2017), where it is shown that extraction from finite (contingent) adjuncts is possible in Swedish, despite being impossible in English (Truswell, 2011). Observations like these, taken in consideration with the results from the current study, suggest that surface variation rather than deep variation in island constraints may be responsible for any variation; and that further investigation at the micro-level (features) is the right way to move forward toward a full understanding of variation in island effects across languages.

## Ethics statement

This study was carried out in accordance with the recommendations of The Swedish Research Council (see Swedish Research Council, 2002: “Forskningsetiska principer inom humanistisk-samhällsvetenskaplig forskning”) with written informed consent from all subjects. The study protocol did not require approval under Swedish national ethics board (Etikprövningsnämnden) rules, and was thus not submitted for review.

## Author contributions

DT experimental design, experiment building, data collection, statistical analysis, interpretation, writing; FH experimental design, stimuli creation, data collection; EK stimuli creation, data collection; A-LW experimental design, interpretation, writing.

### Conflict of interest statement

The authors declare that the research was conducted in the absence of any commercial or financial relationships that could be construed as a potential conflict of interest.

## Footnotes

^1^Swedish is a verb second (V2) language, which means that the finite verb is preceded by at most one constituent in matrix clauses. In the case of extraction, the first constituent (the filler) functions as a sentence topic.

^2^Note that translations are not idiomatic in (4b-d), as the English counterparts to the Swedish sentences are not acceptable.

^3^We assume that there is basic cost for successful integration, and that this cost would be absent in the island conditions, but we expect that this cost is lower than other costs associated with complex structure and islandhood.
