# Supplementary material for: Processing Relative Clause Extractions in Swedish
Source: Front Psychol. 2017 Dec 7;8:2118. doi: 10.3389/fpsyg.2017.02118 (PMC5726081; doi:10.3389/fpsyg.2017.02118)
Supplement: Supplementary file 1 [file Table1.PDF]

Supplementary Material

Processing relative clause extractions in Swedish

Damon Tutunjian[1*], Fredrik Heinat[2], Eva Klingvall[1], Anna-Lena Wiklund[1]

[1]Centre for Languages and Literature, Lund University, Lund, Sweden
[2]Department of Languages, Linnaeus University, Växjö, Sweden

*** Correspondence:**
Damon Tutunjian
damon.tutunjian@englund.lu.se

Experiment 1: critical items

101	RCE	Såna där gamla skottkärror såg jag en man som alltid tvättade på bensinmacken när han var ledig.<br>Such there old whellbarrows saw I a man that always washed at gas-station-the when he was off-duty<br>(*Such old wheelbarrows, I saw a man that always washed at the gas station when he was off duty.)
101	TCE	Såna där gamla skottkärror såg jag att en man alltid tvättade på bensinmacken när han var ledig.<br>Such there old wheelbarrows saw I that a man always washed at gas-station-the when he was off-duty<br>(Such old wheelbarrows, I saw that a man always washed at the gas station when he was off duty.)
101	NRCE	Såna där gamla skottkärror såg jag en man som förresten tvättade på bensinmacken när han var ledig.<br>Such there old wheelbarrows saw I a man that by-the-way washed at gas-station-the when he was off-duty<br>(*Such old wheelbarrows, I saw a man that by the way washed at the gas station when he was off duty.)
101	PCRCE	Såna där gamla skottkärror såg jag en man som alltid stod och tvättade på bensinmacken när han var ledig.<br>Such there old wheelbarrows saw I a man that always stood and washed at gas-station-the when he was off-duty<br>(*Such old wheelbarrows, I saw a man that always stood and washed at the gas station when he was off duty.)
102	RCE	Såna där fina äppelträd såg han en trädgårdsmästare som alltid beskärde på hösten när det var plusgrader.<br>Such there fine apple-tree saw he a gardener that always pruned at autumn-the when it was above-freezing<br>(*Such fine apple trees, he was a gardener that always pruned in the

		autumn when it was above freezing.)
102	TCE	Såna där fina äppelträd såg han att en trädgårdsmästare alltid beskärde på hösten när det var plusgrader.<br>Such there fine apple-tree saw he that a gardener always pruned at autumn when it was above-freezing<br>(Such fine apple trees, he saw that a gardener always pruned in the autumn when it was above freezing.)
102	NRCE	Såna där fina äppelträd såg han en trädgårdsmästare som förresten beskärde på hösten när det var plusgrader.<br>Such there fine apple-tree saw he a gardener that by-the-why pruned at autumn when it was above-freezing<br>(*Such fine apple trees, he saw a gardener that by the way pruned in the autumn when it was above freezing.)
102	PCRCE	Såna där fina äppelträd såg han en trädgårdsmästare som alltid stod och beskärde på hösten när det var plusgrader.<br>Such there fine apple-tree saw he a gardener that always stood and pruned at autumn when it was above-freezing<br>(*Such fine apple trees, he saw a gardener that always stood and pruned in the autumn when it was above freezing.)
103	RCE	Såna där allvarliga konsekvenser antog hon en student som alltid diskuterade på seminarierna för att imponera.<br>Such there serious consequences assumed she a student that always discussed at seminars-the in-order-to to impress<br>(*Such serious consequences, She assumed a student that always discussed at the seminars to impress.)
103	TCE	Såna där allvarliga konsekvenser antog hon att en student alltid diskuterade på seminarierna för att imponera.<br>Such there serious consequences assumed she that a student always discussed at seminars-the in-order-to to impress<br>(Such serious consequences, she assumed that a student always discussed at the seminars to impress.)
103	NRCE	Såna där allvarliga konsekvenser antog hon en student som förresten diskuterade på seminarierna för att imponera.<br>Such there serious consequences assumed she a student that by-the-way discussed at seminars-the in-order-to to impress<br>(*Such serious consequences, she assumed a student that by the way discussed at the seminars to impress.)
103	PCRCE	Såna där allvarliga konsekvenser antog hon en student som alltid satt och diskuterade på seminarierna för att imponera.<br>Such serious consequences assumed she a student that always sat and discussed at seminars-the in-order-to to impress<br>(*Such serious consequences, she assumed a student that always sat and discussed at the seminars to impress.)
104	RCE	Såna där inspirerande föredrag antog vi en sökande som alltid presenterade på mässor för att visa sin kompetens.<br>Such there inspiring lecture assumed we an applicant that always presented at trade-shows in-order-to show his competence

		(*Such inspiring lectures, we assumed an applicant that always presented at trade-shows to show his competence.)
104	TCE	Såna där inspirerande föredrag antog vi att en sökande alltid presenterade på mässor för att visa sin kompetens.<br>Such inspiring lecture assumed we that an applicant always presented at trade-shows in-order-to show his competence<br>(Such inspiring lectures, we assumed that an applicant always presented at trade shows to show his completence.)
104	NRCE	Såna där inspirerande föredrag antog vi en sökande som förresten presenterade på mässor för att visa sin kompetens.<br>Such there inspiring lecture assumed we an applicant that by-the-way presented at trade-shows in-order-to to show his completence<br>(*Such inspiring lectures, we assumed an applicant that by the way presented at trade shows to show his completence.)
104	PCRCE	Såna där inspirerande föredrag antog vi en sökande som alltid kom och presenterade på mässor för att visa sin kompetens.<br>Such there inspiring lecture assumed we an applicant that always came and presented at trade-shows in-order-to show his completence<br>(*Such inspiring lectures, we assumed an applicant that always came and presented at trade shows to show his competence.)
105	RCE	Såna där långa ramsor avskydde jag en lärare som alltid rabblade på lektionerna för att imponera.<br>Such there long chants hated I a teacher that always recited at lessons-the in-order-to to impress<br>(*Such long chants, I hated a teacher that always recited in lessons to impress.)
105	TCE	Såna där långa ramsor avskydde jag att en lärare alltid rabblade på lektionerna för att imponera.<br>Such there long chants hated I that a teacher always recited at lessons-the in-order-to to impress<br>(Such long chants, I hated that a teacher always recited in lessons to impress.)
105	NRCE	Såna där långa ramsor avskydde jag en lärare som förresten rabblade på lektionerna för att imponera.<br>Such there long chants hated I a teacher that by-the-way recited at lessons-the in-order-to to impress<br>(Such long chants, I hated a teacher that by the way recited in lessons to impress.)
105	PCRCE	Såna där långa ramsor avskydde jag en lärare som alltid stod och rabblade på lektionerna för att imponera.<br>Such there long chants hated I a teacher that always stood and recited at lessons-the in-order-to to impress<br>(Such long chants hated I a teacher that always stood and recited at the lessons to impress.)
106	RCE	Såna där kryddiga soppor avskydde hon en kille som alltid lagade efter träningen i korridorköket.<br>Such there spicy soups hated she a boy that always cooked after training-the in corridor-kitchen-the

		(*Such spicy soups, she hated a boy that always cooked after the training in the corridor kitchen.)
106	TCE	Såna där kryddiga soppor avskydde hon att en kille alltid lagade efter träningen i korridorköket.<br>Such there spicy soups hated she that a boy always cooked after training-the in corridor-kitchen-the<br>(Such spicy soups, she hated that a boy always cooked after the training in the corridor kitchen.)
106	NRCE	Såna där kryddiga soppor avskydde hon en kille som förresten lagade efter träningen i korridorköket.<br>Such there spicy soups hated she a boy that by-the-way cooked after training-the in corridor-kitchen-the<br>(*Such spicy soups, she hated a boy that by the way cooked after the training in the corridor kitchen.)
106	PCRCE	Såna där kryddiga soppor avskydde hon en kille som alltid stod och lagade efter träningen i korridorköket.<br>Such there spicy soups hated she a boy that always stood and cooked after training-the in corridor-kitchen-the<br>(Such spicy soups, she hated a boy that always stood and cooked after the training in the corridor kitchen.)
107	RCE	Såna där intressanta vitsord avundades han en kollega som alltid fick under mötena på jobbet.<br>Such there interesting credential envied he a colleague that always got during meetings-the at work-the<br>(*Such interesting credentials, he envied a colleague that always got during meetings at work.)
107	TCE	Såna där intressanta vitsord avundades han att en kollega alltid fick under mötena på jobbet.<br>Such there interesting credential envied he that a colleague always got during meetings-the at work-the<br>(Such interesting credentials, he envied that a colleague always got during meetings at work.)
107	NRCE	Såna där intressanta vitsord avundades han en kollega som förresten fick under mötena på jobbet.<br>Such there interesting credentials envied he a colleague that by-the-way got during meetings-the at work-the<br>(*Such interesting credentials, he envied a colleague that by the way got during meetings at work.)
107	PCRCE	Såna där intressanta vitsord avundades han en kollega som alltid satt och fick under mötena på jobbet.<br>Such interesting credentials envied he a colleague that always sat and got during meetings-the at work-the<br>(*Such interesting credentials, he envied a colleague that always sat and got during meetings at work.)
108	RCE	Såna där prestigefyllda ljusstakar avundades hon en senior som alltid vann under säsongsstarten varje år.<br>such there prestigious candlesticks envied she a senior that always won

during season-start-the every year
(*Such prestigious candlesticks, she envied a senior that always won during the season start every year.)

108 TCE Såna där prestigefyllda ljusstakar avundades hon att en senior alltid vann under säsongsstarten varje år.
Such there prestigious candlesticks envied she that a senior always won during season-start-the every year
(Such prestigious candlesticks, she envied that a senior always won during the season start every year.)

108 NRCE Såna där prestigefyllda ljusstakar avundades hon en senior som förresten vann under säsongsstarten varje år.
Such there prestigious candlesticks envied she a senior that by-the-way won during season-start-the every year
(*Such prestigious candlesticks, she envied a senior that by the way won during the season start every year.)

108 PCRCE Såna där prestigefyllda ljusstakar avundades hon en senior som alltid gick och vann under säsongsstarten varje år.
Such there prestigious candlesticks envied she a senior that always went and won during season-start-the every year
(*Such prestigious candlesticks, she envied a senior that always went and won during the start of the season every year.)

109 RCE Såna där innovativa koncept visste han en ingenjör som framgångsrikt sålde på mässor varje år.
Such there innovative concept knew he an engineer that successfully sold at trade-shows every year
(*Such innovative concepts, he knew an engineer that successfully sold at trade shows every year.)

109 TCE Såna där innovativa koncept visste han att en ingenjör framgångsrikt sålde på mässor varje år.
Such there innovative concept knew he that an engineer successfully sold at trade-shows every year
(Such innovative concepts, he knew that an engineer successfully sold at trade shows every year.)

109 NRCE Såna där innovativa koncept visste han en ingenjör som förresten sålde på mässor varje år.
Such innovative concepts knew he an engineer that by-the-way sold at trade-shows every year
(*Such innovative concepts, he knew an engineer that by the way sold at trade shows every year.)

109 PCRCE Såna där innovativa koncept visste han en ingenjör som framgångsrikt stod och sålde på mässor varje år.
Such there innovative concepts knew he an engineer that successfully stood and sold at trade-shows every year
(*Such innovative concepts, he knew an engineer that successfully stood and sold at trade shows every year.)

110 RCE Såna där smarta utvägar visste hon en direktör som alltid köpte på bolagsstämman för att undvika problem.
Such there smart out-ways knew she a director that always bought at

		agm-the in-order-to to avoid problem<br>(*Such smart ways out, she knew a director that always bought at the agm to avoid problems.)
110	TCE	Såna där smarta utvägar visste hon att en direktör alltid köpte på bolagsstämman för att undvika problem.<br>Such there smart out-ways knew she that a director always bought at agm-the in-order-to avoid problem<br>(Such smart ways out, she knew that a director always bought at the agm to avoid problems.)
110	NRCE	Såna där smarta utvägar visste hon en direktör som förresten köpte på bolagsstämman för att undvika problem.<br>Such there smart out-ways knew she a director that by-the-way bought at agm-the in-order-to avoid problem<br>(*Such smart ways out, she knew a director that by the way bought at the agm to avoid problems.)
110	PCRCE	Såna där smarta utvägar visste hon en direktör som alltid kom och köpte på bolagsstämman för att undvika problem.<br>Such there smart out-ways knew she a director that always came and bought at agm-the in-order-to avoid problem<br>(*Such smart ways out, she knew a director that always came and bought at the agm to avoid problems.)
111	RCE	Såna där vackra arior hörde jag en operasångerska som alltid sjöng om våren när solen sken.<br>Such there beautiful arias hear I an opera-singer that always sang about spring-the when sun-the shined<br>(*Such beautiful arias, I heard an opera singer that always sang in the spring when the sun was shining.)
111	TCE	Såna där vackra arior hörde jag att en operasångerska alltid sjöng om våren när solen sken.<br>Such there beautiful arias head I that an opera-singer always sang about spring-the when sun-the shined<br>(Such beautiful arias, I heard that an opera singer always sang in the spring when the sun was shining.)
111	NRCE	Såna där vackra arior hörde jag en operasångerska som förresten sjöng om våren när solen sken.<br>Such there beautiful arias heard I an opera-singer that by-the-way sang about spring-the when sun-the shined<br>(*Such beautiful arias, I heard an opera singer that by the way sang in the spring when the sun was shining.)
111	PCRCE	Såna där vackra arior hörde jag en operasångerska som alltid stod och sjöng om våren när solen sken.<br>Such tere beautiful arias heard I an opera-singer that always stood and sang about spring-the when sun-the shined<br>(*Such beautiful arias, I heard an opera singer that always stood and sang in the spring when the sun was shining.)
112	RCE	Såna där höga grymtningar hörde jag en gubbe som alltid utstötte på gymmet när han lyfte skrot.

Such there loud grunts heard I an old man that always outbursted at gym-the when he lifted scrap
(*Such loud grunts, I heard an old man that always outbursted in the gym when he lifted scrap.)

112	TCE	Såna där höga grymtningar hörde jag att en gubbe alltid utstötte på gymmet när han lyfte skrot.<br>Such there loud grunts heard I that an old man always outbursted at gym-the when he lifted scrap<br>(Such loud grunts, I heard that an old man always outbursted in the gym when he lifted scrap.)
112	NRCE	Såna där höga grymtningar hörde jag en gubbe som förresten utstötte på gymmet när han lyfte skrot.<br>Such there loud grunts heard I an old man that by-the-way outbursted at gym-the when he lifted scrap<br>(*Such loud grunts, I heard an old man that by the way outbursted in the when gym he lifted scrap.)
112	PCRCE	Såna där höga grymtningar hörde jag en gubbe som alltid låg och utstötte på gymmet när han lyfte skrot.<br>Such there loud grunts heard I an old man that always lied and outbursted at gym-the when he lifted scrap<br>(*Such loud grunts, I heard an old man that always lied and outbursted at the gym when he lifted scrap.)
113	RCE	Såna där avancerade tricks upptäckte vi en forskare som redan använde i experiment i labbet.<br>Such there advanced trcks discovered we a researcher that already used in experiment in lab-the<br>(*Such advanced tricks, we discovered a researcher that already used in experiments in the lab.)
113	TCE	Såna där avancerade tricks upptäckte vi att en forskare redan använde i experiment i labbet.<br>Such there advanced tricks discovered we that a researcher already used in experiment in lab-the<br>(Such advanced tricks, we discovered that a researcher already used in experiments in the lab.)
113	NRCE	Såna där avancerade tricks upptäckte vi en forskare som förresten använde i experiment i labbet.<br>Such there advanced tricks discovered we a researcher that by-the-way already used in experiment in lab-the<br>(*Such advanced tricks, we discovered a researcher that by the way used in experiments in the lab.)
113	PCRCE	Såna där avancerade tricks upptäckte vi en forskare som redan stod och använde i experiment i labbet.<br>Such there advanced tricks discovered we a researcher that already stood and used in experiment in lab-the<br>(*Such advanced tricks, we discovered a researcher that already stood and used in experiments in the lab.)
114	RCE	Såna där krångliga mönster upptäckte hon en tant som alltid broderade på dukarna under symötena.

Such there complex patterns discovered she an aunt that always embroidered on canvases during sewing-meetings-the
(*Such complex patterns, she discovered an aunt that always embroidered on the canvases during the sewing meetings.)

114 TCE Såna där krångliga mönster upptäckte hon att en tant alltid broderade på dukarna under symötena.
Such there complex patterns discovered she that an aunt always embroidered on canvases-the during sewnig-meetings-the
(Such complex patterns, she discovered that an aunt always embroidered on the canvases during the sewing meetings.)

114 NRCE Såna där krångliga mönster upptäckte hon en tant som förresten broderade på dukarna under symötena.
Such there complex patterns discovered she an aunt that by-the-way embroidered on canvases-the during sewing-meetings-the
(*Such complex patterns, she discovered an aunt that by the way embroidered on the canvases during the sewing meetings.)

114 PCRCE Såna där krångliga mönster upptäckte hon en tant som alltid satt och broderade på dukarna under symötena.
Such there complex patterns discovered she an aunt that always sat and embroidered on canvases-the during sewing-meetings-the
(*Such complex patterns discovered she an aunt that always sat and embroidered on the canvases during sewing meetings.)

115 RCE Såna där skrovliga fotsulor kände han en pedikyrist som aldrig accepterade på kunderna efter en behandling.
Such there rough soles felt he a pedicurist that never accepted on customers-the after a treatment
(*Such rough soles, he felt a pedicurist that never accepted on the customers after a treatment.)

115 TCE Såna där skrovliga fotsulor kände han att en pedikyrist aldrig accepterade på kunderna efter en behandling.
Such there rough soles felt he that a pedicurist never accepted on customers-the after a treatment
(Such rough soles, he felt that a pedicurist never accepted on the customers after a treatment.)

115 NRCE Såna där skrovliga fotsulor kände han en pedikyrist som förresten accepterade på kunderna efter en behandling.
Such there rough soles felt he a pedicurist that by-the-way accepted on customers-the after a treatment
(*Such rough soles, he felt a pedicurist that by the way accepted on the customers after a treatment.)

115 PCRCE Såna där skrovliga fotsulor kände han en pedikyrist som aldrig kom och accepterade på kunderna efter en behandling.
Such there rough soles felt he a pedicurist that never came and accepted on customers-the after a treatment
(*Such rough soles, he felt a pedicurist that never came and accepted on the customers after a treatment.)

116 RCE Såna där stickiga arbetskläder kände hon en anställd som gärna bar i

verkstaden trots att de kliade.
Such there scratchy overalls felt she an employee that gladly wore in workshop-the although that they scratched
(Such scratchy overalls, she felt an employee that gladly wore in the workshop although they scratched.)

116 TCE Såna där stickiga arbetskläder kände hon att en anställd gärna bar i verkstaden trots att de kliade.
Such there scratchy overalls felt she that an employee gladly wore in workshop-the although that they scratched
(Such scratchy overalls, she felt that an employee gladly wore in the workshop although they scratched.)

116 NRCE Såna där stickiga arbetskläder kände hon en anställd som förresten bar i verkstaden trots att de kliade.
Such there scratchy overalls felt she an employee that by-the-way wore in workshop-the although that they scratched
(*Such scratchy overalls, she felt an employee that by the way wore in the workshop although they scratched.)

116 PCRCE Såna där stickiga arbetskläder kände hon en anställd som gärna gick och bar i verkstaden trots att de kliade.
Such there scratchy overalls felt she an employee that gladly went and wore in workshop-the although that they scratched
(Such scratchy overalls felt she an employee that gladly went and wore in the workshop although they scratched.)

117 RCE Såna där goda skorpor älskade han en kusin som alltid bakade till jul varje år.
Such there good biscuits loved he a cousin that always baked for christmas every year
(*Such good biscuits, he loved a cousin that always baked for christmas every year.)

117 TCE Såna där goda skorpor älskade han att en kusin alltid bakade till jul varje år.
Such there good biscuits loved he that a cousin always baked for christmas every year
(Such good biscuits, he loved that a cousin always baked for christmas every year.)

117 NRCE Såna där goda skorpor älskade han en kusin som förresten bakade till jul varje år.
Such there good biscuits loved he a cousin that by-the-way baked for christmas every year
(*Such good biscuits, he loved a cousin that by the way baked for christmas every year.)

117 PCRCE Såna där goda skorpor älskade han en kusin som alltid kom och bakade till jul varje år.
Such there good biscuits loved he a cousin that always came and baked for christmas every year
(*Such good biscuits, he loved a cousin that always came and baked for christmas every year.)

118 RCE Såna där söta violer älskade jag ett grannbarn som alltid plockade till

		midsommar för att klä stången.<br>Such there cute violets loved I a neighbor-child that always picked for midsummer in-order-to to dress bar-the<br>(*Such cute violets, I loved a neighbor-child that always picked for midsummer to dress the bar.)
118	TCE	Såna där söta violer älskade jag att ett grannbarn alltid plockade till midsommar för att klä stången.<br>Such there cute violets loved that a neighbor-child always picked for midsummer in-order-to to dress bar-the<br>(Such cute violets, I loved that a neighbor-child always picked for midsummer to dress the bar.)
118	NRCE	Såna där söta violer älskade jag ett grannbarn som förresten plockade till midsommar för att klä stången.<br>Such there cute violets loved I a neighbor-child that by-the-way picked for midsummer in-order-to to dress bar-the<br>(*Such cute violets, I loved a neighbor-child that by the way picked for midsummer to dress the bar.)
118	PCRCE	Såna där söta violer älskade jag ett grannbarn som alltid kom och plockade till midsommar för att klä stången.<br>Such there cute violets loved I a neighbor-child that always came and picked for midsummer in-order-to to dress bar-the<br>(*Such cute violets, I loved a neighbor-child that always came and picked for midsummer to dress the bar.)
119	RCE	Såna där delikata tårtor uppskattade vi en sekreterare som alltid serverade till kaffet för att muntra upp oss.<br>Such there delicious cakes estimated we a secretary that always served with coffee-the in-order-to to cheer up us<br>(*Such delicious cakes, we estimated a secretary that always served with the coffee to cheer us up.)
119	TCE	Såna där delikata tårtor uppskattade vi att en sekreterare alltid serverade till kaffet för att muntra upp oss.<br>Such there delicious cakes estimated we that a secretary always served with coffee-the in-order-to to cheer up us<br>(Such delicious cakes, we estimated that a secretary always served with the coffee to cheer us up.)
119	NRCE	Såna där delikata tårtor uppskattade vi en sekreterare som förresten serverade till kaffet för att muntra upp oss.<br>Such there delicious cakes estimated we a secretary that by-the-way served with coffee-the in-order-to to cheer up us<br>(*Such delicious cakes, we estimated a secretary that by the way served with the coffee to cheer us up.)
119	PCRCE	Såna där delikata tårtor uppskattade vi en sekreterare som alltid kom och serverade till kaffet för att muntra upp oss.<br>Such there delicious cakes estimated we a secretary that always came and served with coffee-the in-order-to to cheer up us<br>(Such delicious cakes, we estimated a secretary that always came and served with the coffee to cheer us up.)

120	RCE	Såna där fantastiska pajer uppskattade han en deltagare som ofta inhandlade till träffen för att imponera.<br>Such there amazing pies estimated he a participant that often purchased for meeting-the in-order-to to impress<br>(*Such amazing pies, he estimated a participant that often purchased for the meeting to impress.)
120	TCE	Såna där fantastiska pajer uppskattade han att en deltagare ofta inhandlade till träffen för att imponera.<br>Such there amazing pies estimated he that a participant often purchased for meeting-the in-order-to to impress<br>(Such amazing pies, he estimated that a participant often purchased for the meeting to impress.)
120	NRCE	Såna där fantastiska pajer uppskattade han en deltagare som förresten inhandlade till träffen för att imponera.<br>Such there amazing pies estimated he a participant that by-the-way purchased for meeting-the in-order-to to impress<br>(*Such amazing pies, he estimated a participant that by the way purchased for the meeting to impress.)
120	PCRCE	Såna där fantastiska pajer uppskattade han en deltagare som ofta gick och inhandlade till träffen för att imponera.<br>Such there amazing pies estimated he a participant that often went and purchased for meeting-the in-order-to impress<br>(*Such amazing pies, he estimated a participant that often went and purchased for the meeting to impress.)
121	RCE	Såna där coola mixar gillade hon en dj som alltid spelade på klubben när hon var där.<br>Such there cool mixes liked she a dj that always played at club-the when she was there<br>(*Such cool mixes, she liked a dj that always played at the club when she was there.)
121	TCE	Såna där coola mixar gillade hon att en dj alltid spelade på klubben när hon var där.<br>Such there cool mixes like shed that a dj always played at klub-the when she was there<br>(Such cool mixes, she liked that a dj always played at the club when she was there.)
121	NRCE	Såna där coola mixar gillade hon en dj som förresten spelade på klubben när hon var där.<br>Such there cool mixes liked she a dj that by-the-way played at club-the when she was there<br>(*Such cool mixes, she liked a dj that by the way played at the club when she was there.)
121	PCRCE	Såna där coola mixar gillade hon en dj som alltid stod och spelade på klubben när hon var där.<br>Such there cool mixes liked she a dj that always stood and played at club-the when she was there<br>(*Such cool mixes, she liked a dj that always stood and played at the club when she was there.)

122	RCE	Såna där korta frisyrer gillade jag en frisör som alltid klippte till sommaren på alla sina kunder.<br>Such there short hairstyles liked I a hairdresser that always cut for summer-the on all her customers<br>(*Such short hairstyles, I liked a hairdresser that always cut for summer to all her customers.)
122	TCE	Såna där korta frisyrer gillade jag att en frisör alltid klippte till sommaren på alla sina kunder.<br>Such there short hairstyles liked I that a hairdresser always cut for summer-the on all her clients<br>(Such short hairstyles, I liked that a hairdresser always cut for summer to all her customers.)
122	NRCE	Såna där korta frisyrer gillade jag en frisör som förresten klippte till sommaren på alla sina kunder.<br>Such there short hairstyles liked I a hairdresser that by-the-way cut for summer-the on all her customers<br>(*Such short hairstyles, I liked a hairdresser that by the way cut for summer to all its customers.)
122	PCRCE	Såna där korta frisyrer gillade jag en frisör som alltid kom och klippte till sommaren på alla sina kunder.<br>Such there short hairstyles liked I a hairdresser that always came and cut for summer-the on all her clients<br>(*Such short hairstyles, I liked a hairdresser that always came and cut for summer to all its customers.)
123	RCE	Såna där jobbiga upphopp ogillade jag en hockeytränare som alltid gjorde på träningen när vi var som tröttast.<br>Such there annoying take-offs disliked I a hockey.coach that always did at training-the when we were as tired-most<br>(*Such annoying take-offs, I disliked a hockey coach that always did at the training when we were most tired.)
123	TCE	Såna där jobbiga upphopp ogillade jag att en hockeytränare alltid gjorde på träningen när vi var som tröttast.<br>Such there annoying take-offs disliked I that a hockey-coach always did at training-the when we were as tired-most<br>(Such annoying take-offs, I disliked that a hockey coach always did at the training when we were most tired.)
123	NRCE	Såna där jobbiga upphopp ogillade jag en hockeytränare som förresten gjorde på träningen när vi var som tröttast.<br>Such there annoying take-offs disliked I a hockey-coach that by-the-way did at training-the when we were as tired-most<br>(*Such annoying take-offs, I disliked a hockey coach that by the way did at the training when we were most tired.)
123	PCRCE	Såna där jobbiga upphopp ogillade jag en hockeytränare som alltid stod och gjorde på träningen när vi var som tröttast.<br>Such there annoying take-offs disliked I a hockey-coach that always stood and did at training-the when we were as tired-most<br>(*Such annoying take-offs, I disliked a hockey coach that always stood

and did at the training when we were most tired.)

124 RCE Såna där hemska hurrarop ogillade han en vaktmästare som ofta skrek på skolgården för att skrämma barnen.
Such there horrible hooray disliked he a janitor that often screamed at schoolyard-the in-order-to to scare children-the
(*Such horrible hoorays, he disliked a janitor that often screamed in the schoolyard to scare the children.)

124 TCE Såna där hemska hurrarop ogillade han att en vaktmästare ofta skrek på skolgården för att skrämma barnen.
Such there horrible hooray disliked he that a janitor often screamed at schoolyard-the in-order-to to scare children-the
(Such horrible hoorays, he disliked that a janitor often screamed in the schoolyard to scare the children.)

124 NRCE Såna där hemska hurrarop ogillade han en vaktmästare som förresten skrek på skolgården för att skrämma barnen.
Such there horrible hooray disliked he a janitor that by-the-way screamed at schoolyard-the in-order-to to scare children-the
(*Such horrible hoorays, he disliked a janitor that by the way screamed in the schoolyard to scare the children.)

124 PCRCE Såna där hemska hurrarop ogillade han en vaktmästare som ofta gick och skrek på skolgården för att skrämma barnen.
Such there horrible hooray disliked he a janitor that often went and screamed at schoolyard-the in-order-to to scare children-the
(*Such horrible hoorays, he disliked a janitor that often went and screamed in the schoolyard to scare the children.)

125 RCE Såna där sedelärande fabler saknade hon en barnflicka som alltid läste på kvällen innan hon skulle sova.
Such there moral fables missed she a nanny that always read at night-the before she would sleep
(*Such moral fables, she missed a nanny that always read at night before she would sleep.)

125 TCE Såna där sedelärande fabler saknade hon att en barnflicka alltid läste på kvällen innan hon skulle sova.
Such there moral fables missed she that a nanny always read at night-the before she would sleep
(Such moral fables, she missed that a nanny always read at night before she would sleep.)

125 NRCE Såna där sedelärande fabler saknade hon en barnflicka som förresten läste på kvällen innan hon skulle sova.
Such there moral fables missed she a nanny that by-the-way read at night-the before she would sleep
(*Such moral fables, she missed a nanny that by the way read at night before she would sleep.)

125 PCRCE Såna där sedelärande fabler saknade hon en barnflicka som alltid satt och läste på kvällen innan hon skulle sova.
Such there moral fables missed she a nanny that always sat and read at night-the before she would sleep
(*Such moral fables, she missed a nanny that always sat and read at night

before she would sleep.)

126 RCE Såna där mysiga kojor saknade han en kompis som gladeligen snickrade på tomten i äppelträden.
Such there cozy huts missed he a buddy that gladly crafted on plot-the in apple-trees-the
(*Such cozy huts, he missed a buddy that gladly crafted on the plot in the apple trees.)

126 TCE Såna där mysiga kojor saknade han att en kompis gladeligen snickrade på tomten i äppelträden.
Such there cozy missed he that a buddy gladly crafted on plot-the in apple-tree-the
(Such cozy huts, he missed that a buddy gladly crafted on the plot in the apple trees.)

126 NRCE Såna där mysiga kojor saknade han en kompis som förresten snickrade på tomten i äppelträden.
Such cozy huts missed he a buddy that by-the-way crafted on plot-the in apple-tree-the
(*Such cozy huts, he missed a buddy that by the way crafted on the plot in the apple trees.)

126 PCRCE Såna där mysiga kojor saknade han en kompis som gladeligen kom och snickrade på tomten i äppelträden.
Such there cozy huts missed he a buddy that gladly came and crafted on plot-the in apple-tree-the
(*Such cozy huts, he missed a buddy that gladly came and crafted on the plot in the apple trees.)

127 RCE Såna där invecklade ärenden rekommenderade vi en advokat som alltid ombesörjde per mejl för att påskynda allt.
Such there complex errands recommended we a lawyer that always arranged by mail in-order-to to up-speed everything
(*Such complex errands, we recommended a lawyer that always arranged by mail to speed up everything.)

127 TCE Såna där invecklade ärenden rekommenderade vi att en advokat alltid ombesörjde per mejl för att påskynda allt.
Such there complex errands recommended we that a lawyer always arranged by mail in-order-to to up-speed everything
(Such complex errands, we recommended that a lawyer always arranged by mail to speed up everything.)

127 NRCE Såna där invecklade ärenden rekommenderade vi en advokat som förresten ombesörjde per mejl för att påskynda allt.
Such there complex errands recommended we a lawyer that by-the-wa arranged by mail in-order-to to up-speed everything
(*Such complex errands, we recommended a lawyer that by the way arranged by mail to speed up everything.)

127 PCRCE Såna där invecklade ärenden rekommenderade vi en advokat som alltid satt och ombesörjde per mejl för att påskynda allt.
Such there complex errands recommended we a lawyer that always sat and arranged by mail in-order-to up-speed everything

(Such complex errands, we recommended a lawyer that always sat and arranged by mail to speed up everything.)

128 RCE Såna där miljövänliga påsar rekommenderade jag ett företag som billigt tillverkade inom EU till en låg kostnad.
Such there environmentally-friendly bags recommended I a business that cheap manufactured within EU at a low cost
(*Such environmentally friendly bags, I recommended a business that cheaply manufactured within the eu at a low cost.)

128 TCE Såna där miljövänliga påsar rekommenderade jag att ett företag billigt tillverkade inom EU till en låg kostnad.
Such there environmentally-friendly bags recommended I that a business cheap manufactured within EU at a low cost
(Such environmentally friendly bags, I recommended that a business cheap manufactured within the eu at a low cost.)

128 NRCE Såna där miljövänliga påsar rekommenderade jag ett företag som förresten tillverkade inom EU till en låg kostnad.
Such there environmentally-friendly bags recommended I a business that by-the-way manufactured within EU at a low cost
(*Such environmentally friendly bags, I recommended a business that by the way manufactured within the eu at a low cost.)

128 PCRCE Såna där miljövänliga påsar rekommenderade jag ett företag som billigt kom och tillverkade inom EU till en låg kostnad.
Such there environmentally-friendly bags recommended I a business that cheap came and manufactured within EU at a low cost
(*Such environmentally friendly bags, I recommended a business that cheaply came and manufactured within the eu at a low cost.)

129 RCE Såna där suggestiva bilder beundrade hon en fotograf som skickligt komponerade i Malmö till en utställning.
Such there suggestive pictures admired she a photographer that ably composed in Malmö for an exhibition
(*Such suggestive pictures, she admired a photographer that ably composed in Malmö for an exhibition.)

129 TCE Såna där suggestiva bilder beundrade hon att en fotograf skickligt komponerade i Malmö till en utställning.
Such there suggestive pictures admired she that a photographer ably composed in Malmö for an exhibition
(Such suggestive pictures, she admired that a photographer ably composed in Malmö for an exhibition.)

129 NRCE Såna där suggestiva bilder beundrade hon en fotograf som förresten komponerade i Malmö till en utställning.
Such there suggestive pictures admired she a photographer that by-the-way composed in Malmö for an exhibition
(*Such suggestive pictures, she admired a photographer that by the way composed in Malmö for an exhibition.)

129 PCRCE Såna där suggestiva bilder beundrade hon en fotograf som skickligt stod och komponerade i Malmö till en utställning.
Such there suggestive pictures admired she a photographer that ably stood and composed in Malmö for an exhibtion

		(*Such suggestive pictures, she admired a photographer that ably stood and composed in Malmö for an exhibition.)
130	RCE	Såna där ovanliga material beundrade han en konstnär som alltid utnyttjade till statyer fast det var ömtåligt.<br>Such there rare material admired he an artist that always utilized for statues though it was fragile<br>(*Such rare material, he admired an artist that always utilized for statues though it was fragile.)
130	TCE	Såna där ovanliga material beundrade han att en konstnär alltid utnyttjade till statyer fast det var ömtåligt.<br>Such there rare material admired he that an artist always utilized for statues though it was fragile<br>(Such rare material, he admired that an artist always utilized for statues though it was fragile.)
130	NRCE	Såna där ovanliga material beundrade han en konstnär som förresten utnyttjade till statyer fast det var ömtåligt.<br>Such there rare material admired he an artist that by-the-way utilized for statues though it was fragile<br>(*Such rare material, he admired an artist that by the way utilized for statues though it was fragile.)
130	PCRCE	Såna där ovanliga material beundrade han en konstnär som alltid kom och utnyttjade till statyer fast det var ömtåligt.<br>Such there rare material admired he an artist that always came and utilized for statues though it was fragile<br>(*Such rare material, he admired an artist that always came and utilized for statues though it was fragile.)
131	RCE	Såna här rangliga cykelställ bevittnade vi en yngling som högljutt vandaliserade under ilska förra helgen.<br>Such here rickety bike-rack witnessed we a youth that loudly vandalized in anger last weekend-the<br>(*Such rickety bike racks, we witnessed a youth that loudly vandalized in anger last weekend.)
131	TCE	Såna här rangliga cykelställ bevittnade vi att en yngling högljutt vandaliserade under ilska förra helgen.<br>Such here rickety bike-rack witnessed we that a youth loudly vandalized in anger last weekend-the<br>(Such rickety bike racks, we witnessed that a youth loudly vandalized in anger last weekend.)
131	NRCE	Såna här rangliga cykelställ bevittnade vi en yngling som förresten vandaliserade under ilska förra helgen.<br>Such here rickety bike-rack witnessed we a youth that by-the-way vandalized in anger last weekend-the<br>(*Such rickety bike racks, we witnessed a youth that by the way vandalized in anger last weekend.)
131	PCRCE	Såna här rangliga cykelställ bevittnade vi en yngling som högljutt stod och vandaliserade under ilska förra helgen.<br>Such here rickety bike-rack witnessed we a youth that loudly stood and

vandalized in anger last weekend-the
(Such rickety bike racks witnessed we a youth that loudly stood and vandalized in anger last weekend.)

132 RCE Såna här exklusiva motorcyklar bevittnade jag en polis som lagenligt konfiskerade i fredags på morgonen.
Such here exclusive motorcycles witnessed I a police.officer that lawfully confiscated on friday in morning-the
(*Such exclusive motorcycles, I witnessed a police officer that lawfully confiscated last friday in the morning.)

132 TCE Såna här exklusiva motorcyklar bevittnade jag att en polis lagenligt konfiskerade i fredags på morgonen.
Such here exclusive motorcycles witnessed I that a police-officer lawfully confiscated on friday in morning-the
(Such exclusive motorcycles, I witnessed that a police officer lawfully confiscated last friday in the morning.)

132 NRCE Såna här exklusiva motorcyklar bevittnade jag en polis som förresten konfiskerade i fredags på morgonen.
Such here exclusive motorcycles witnessed I a police-officer that by-the-way confiscated on friday in morning-the
(*Such exclusive motorcycles, I witnessed a police officer that by the way confiscated last friday in the morning.)

132 PCRCE Såna här exklusiva motorcyklar bevittnade jag en polis som lagenligt kom och konfiskerade i fredags på morgonen.
Such here exclusive motorcycles witnessed I a police-officer that lawfully came and confiscated on friday in morning-the
(*Such exclusive motorcycles, I witnessed a police officer that lawfully came and confiscated last friday in the morning.)

133 RCE Såna där skyhöga avier diskuterade vi en hyresgäst som aldrig betalade i tid trots flera påminnelser.
Such there sky-high slips discussed we a tenant that never payed in time despite several reminders
(*Such sky-high slips, we discussed a tenant that never paid in time despite several reminders.)

133 TCE Såna där skyhöga avier diskuterade vi att en hyresgäst aldrig betalade i tid trots flera påminnelser.
Such there sky-high slips discussed we that a tenant never payed in time despite several reminders
(Such sky-high slips, we discussed that a tenant never paid in time despite several reminders.)

133 NRCE Såna där skyhöga avier diskuterade vi en hyresgäst som förresten betalade i tid tack vare flera påminnelser.
Such there sky-high slips discussed we a tenant that by-the-way payed in time thanks to several reminders
(*Such sky-high slips, we discussed a tenant that by the way paid in time thanks to several reminders.)

133 PCRCE Såna där skyhöga avier diskuterade vi en hyresgäst som aldrig kom och betalade i tid trots flera påminnelser.
Such there sky-high slips discussed we a tenant that never came and

		payed in time despite several reminders<br>(Such sky-high slips, we discussed a tenant that never came and paid in time despite several reminders.)
134	RCE	Såna där okonventionella strategier diskuterade vi en vikarie som alltid tillämpade i klassrummet inför provet.<br>Such there unconventional strategies discussed we a substitute that always applied in class-room-the before test-the<br>(*Such unconventional strategies, we discussed a substitute that always applied in the classroom in preparation for the test.)
134	TCE	Såna där okonventionella strategier diskuterade vi att en vikarie alltid tillämpade i klassrummet inför provet.<br>Such there unconventional strategies discussed we that a substitute always applied in class-room-the before test-the<br>(Such unconventional strategies, we discussed that a substitute always applied in the classroom in preparation for the test.)
134	NRCE	Såna där okonventionella strategier diskuterade vi en vikarie som förresten tillämpade i klassrummet inför provet.<br>Such there unconventional strategies discussed we a substitute that by-the-way applied in class-room-the before test-the<br>(*Such unconventional strategies, we discussed a substitute that by the way applied in the classroom in preparation for the test.)
134	PCRCE	Såna där okonventionella strategier diskuterade vi en vikarie som alltid kom och tillämpade i klassrummet inför provet.<br>Such there unconventional strategies discussed we a substitute that always came and applied in class-room-the before test-the<br>(Such unconventional strategies, we discussed a substitute that always came and applied in the classroom in preparation for the test.)
135	RCE	Såna där magnifika tavlor dolde hon en man som skickligt förfalskade i källaren utan att någon märkte det.<br>Such there magnificent paintings concealed she a man that ably forged in basement-the without that anyone noticed it<br>(*Such magnificent paintings, she concealed a man that ably forged in the basement without anyone noticing.)
135	TCE	Såna där magnifika tavlor dolde hon att en man skickligt förfalskade i källaren utan att någon märkte det.<br>Such there magnificent paintings concealed she that a man ably forged in basement-the without that anyone noticed it<br>(Such magnificent paintings, she concealed that a man ably forged in the basement without anyone noticing.)
135	NRCE	Såna där magnifika tavlor dolde hon en man som förresten förfalskade i källaren utan att någon märkte det.<br>Such there magnificent paintings concealed she a man that by-the-way forged in basement-the without that anyone noticed it<br>(*Such magnificent paintings, she concealed a man that by the way forged in the basement without anyone noticing.)
135	PCRCE	Såna där magnifika tavlor dolde hon en man som skickligt satt och förfalskade i källaren utan att någon märkte det.

Such there magnificent paintings concealed she a man that ably sat forged in basement-the without that anyone noticed it
(*Such magnificent paintings, she concealed a man that ably sat and forged in the basement without anyone noticing.)

136 RCE Såna där olagliga flygblad dolde han en motståndsman som ibland kopierade på vinden under kriget.
Such there unlawful flyer concealed he a resistant that sometimes copied in attic-the during war-the
(*Such unlawful flyers, he concealed a resistant that sometimes copied in the attic during the war.)

136 TCE Såna där olagliga flygblad dolde han att en motståndsman ibland kopierade på vinden under kriget.
Such there unlawful flyer concealed he that a resistant sometimes copied in attic-the during war-the
(Such unlawful flyers, he concealed that a resistant sometimes copied in the attic during the war.)

136 NRCE Såna där olagliga flygblad dolde han en motståndsman som förresten kopierade på vinden under kriget.
Such there unlawful flyer concealed he a resistant that by-the-way copied in attic-the during war-the
(*Such unlawful flyers, he concealed a resistant that by the way copied in the attic during the war.)

136 PCRCE Såna där olagliga flygblad dolde han en motståndsman som ibland stod och kopierade på vinden under kriget.
Such there unlawful flyer concealed he a resistant that sometimes stood and copied in attic-the during war-the
(*Such unlawful flyers, he concealed a resistant that sometimes stood and copied in the attic during the war.)

137 RCE Såna där mustiga ölsorter fann hon en irländare som alltid bryggde på landet i sin sommarstuga.
Such there gusty beers found she an irishman that always brewed on countryside-the in his summer-cottage
(*Such gutsy beers, she found an irishman that always brewed on the countryside in his summer cottage.)

137 TCE Såna där mustiga ölsorter fann hon att en irländare alltid bryggde på landet i sin sommarstuga.
Such there gusty beers found she that an irishman always brewed on countryside-the in his summer-cottage
(Such gutsy beers, she found that an irishman always brewed on the countryside in his summer cottage.)

137 NRCE Såna där mustiga ölsorter fann hon en irländare som förresten bryggde på landet i sin sommarstuga.
Such there gusty beers found she an irishman that by-the-way brewed on countryside-the in his summer-cottage
(Such gutsy beers, she found an irishman that by the way brewed on the countryside in his summer cottage.)

137 PCRCE Såna där mustiga ölsorter fann hon en irländare som alltid stod och bryggde på landet i sin sommarstuga.

Such there gusty beers found she an irishman that always stood and brewed on countryside-the in his summer-cottage
(*Such gutsy beers, she found an irishman that always stood and brewed on the countryside in his summer cottage.)

138 RCE Såna där trista göromål fann han en arbetslös som villigt utförde på nolltid utan invändning.
Such there boring duties found he an unemployed that willingly performed in no-time without objection
(*Such boring duties, he found an unemployed that willingly performed in no time without objection.)

138 TCE Såna där trista göromål fann han att en arbetslös villigt utförde på nolltid utan invändning.
Such there boring duties found he that an unemployed willingly performed in no-time without objection
(Such boring duties, he found that an unemployed willingly performed in no time without objection.)

138 NRCE Såna där trista göromål fann han en arbetslös som förresten utförde på nolltid utan invändning.
Such there boring duties found he an unemployed that by-the-way performed in no-time without objection
(*Such boring duties, he found an unemployed that by the way performed in no time without objection.)

138 PCRCE Såna där trista göromål fann han en arbetslös som villigt kom och utförde på nolltid utan invändning.
Such there boring duties found he an unemployed that willingly came and performed in no-time without objection
(*Such boring duties, he found an unemployed that willingly came and performed in no time without objection.)

139 RCE Såna där illaluktande sopor fixade vi en firma som regelbundet hämtade mot betalning under sommaren.
Such there malodorous garbage fixed we a company that regularly fetched for payment during summer-the
(*Such malodorous garbage, we fixed a company that regularly fetched for payment during the summer.)

139 TCE Såna där illaluktande sopor fixade vi att en firma regelbundet hämtade mot betalning under sommaren.
Such there malodorous garbage fixed we that a company regularly fetched for payment during summer-the
(Such malodorous garbage, we fixed that a company regularly fetched for payment during the summer.)

139 NRCE Såna där illaluktande sopor fixade vi en firma som förresten hämtade mot betalning under sommaren.
Such there malodorous garbage fixed we a company that by-the-way fetched for payment during summer-the
(*Such malodorous garbage, we fixed a company that by the way fetched for payment during the summer.)

139 PCRCE Såna där illaluktande sopor fixade vi en firma som regelbundet kom och

hämtade mot betalning under sommaren.
Such there malodorous garbage fixed we a company that regularly came and fetched for payment during summer-the
(*Such malodorous garbage, we fixed a company that regularly came and fetched for payment during the summer.)

140 RCE Såna här antika skåp fixade hon en snickare som proffsigt restaurerade på plats på ett par timmar.
Such there antique cabinet fixed she a carpenter that professionally restored in place in a few hours
(*Such antique cabinets, she fixed a carpenter that professionally restored in place in a few hours.)

140 TCE Såna här antika skåp fixade hon att en snickare proffsigt restaurerade på plats på ett par timmar.
Such there antique cabinet fixed that she a carpenter professionally restored in place in a few hours
(Such antique cabinets, she fixed that a carpenter professionally restored in place in a few hours.)

140 NRCE Såna här antika skåp fixade hon en snickare som förresten restaurerade på plats på ett par timmar.
Such there antique cabinet fixed she a carpenter that by-the-way restored in place in a few hours
(*Such antique cabinets, she fixed a carpenter that by the way restored in place in a few hours.)

140 PCRCE Såna här antika skåp fixade hon en snickare som proffsigt kom och restaurerade på plats på ett par timmar.
Such there antique cabinet fixed she a carpenter that professionally came and restored in place in a few hours
(*Such antique cabinets, she fixed a carpenter that professionally came and restored in place in a few hours.)

141 RCE Såna här specifika förutsättningar framhävde jag en person som definitivt uppfyllde under intervjun i veckan.
Such here specific conditions highlighted I a person that definitely met during interview-the in week-the
(*Such specific conditions, I highlighted a person that definitely met during the interview this week.)

141 TCE Såna här specifika förutsättningar framhävde jag att en person definitivt uppfyllde under intervjun i veckan.
Such here specific conditions highlighted I that a person definitely met during interview-the in week-the
(Such specific conditions, I highlighted that a person definitely met during the interview this week.)

141 NRCE Såna här specifika förutsättningar framhävde jag en person som förresten uppfyllde under intervjun i veckan.
Such here specific conditions highlighted I a person that by-the-way met during interview-the in week-the
(*Such specific conditions, I highlighted a person that by the way met during the interview this week.)

141 PCRCE Såna här specifika förutsättningar framhävde jag en person som definitivt

kom och uppfyllde under intervjun i veckan.
Such here specific conditions highlighted I a person that definitely came and met during interview-the in week-the
(*Such specific conditions, I highlighted a person that definitely came and met during the interview of the week.)

142 RCE
Såna här fina vitsord framhävde vi en student som klart förtjänade för uppsatsen trots sin sjukfrånvaro.
Such fine credentials highlighted we a student that clearly deserved for essay-the despite his sick-leave
(*Such fine credentials, we highlighted a student that clearly deserved the essay despite his sick leave.)

142 TCE
Såna här fina vitsord framhävde vi att en student klart förtjänade för uppsatsen trots sin sjukfrånvaro.
Such fine credentials highlighted we that a student clearly deserved for essay-the despite his sick-leave
(Such fine credentials, we highlighted that a student clearly deserved the essay despite his sick leave.)

142 NRCE
Såna här fina vitsord framhävde vi en student som förresten förtjänade för uppsatsen trots sin sjukfrånvaro.
Such fine credentials highlighted we a student that by-the-way deserved for essay-the despite his sick-leave
(*Such fine credentials, we highlighted a student that by the way deserved the essay despite his sick leave.)

142 PCRCE
Såna här fina vitsord framhävde vi en student som klart kom och förtjänade för uppsatsen trots sin sjukfrånvaro.
Such fine credentials highlighted we a student that clearly came and deserved for essay-the despite his sick-leave
(*Such fine credentials, we highlighted a student that clearly came and deserved the essay despite his sick leave.)

143 RCE
Såna här förbjudna preparat fruktade han en patient som ibland nyttjade under permissionen för att få lugn.
Such here forbidden preparations feared he a patient that sometimes exploited during leave-the in-order-to to get calm
(*Such forbidden preparations, he feared a patient that sometimes exploited during the leave to get calm.)

143 TCE
Såna här förbjudna preparat fruktade han att en patient ibland nyttjade under permissionen för att få lugn.
Such here forbidden preparations feared that he a patient sometimes exploited during leave-the in-order-to to get calm
(Such forbidden preparations, he feared that a patient sometimes exploited during the leave to get calm.)

143 NRCE
Såna här förbjudna preparat fruktade han en patient som förresten nyttjade under permissionen för att få lugn.
Such here forbidden preparations feared he a patient that by-the-way exploited during leave-the in-order-to to get calm
(*Such forbidden preparations, he feared a patient that by the way exploited during the leave to get calm.)

143	PCRCE	Såna här förbjudna preparat fruktade han en patient som ibland gick och nyttjade under permissionen för att få lugn.<br>Such here forbidden preparations feared he a patient that sometimes went and exploited during leave-the in-order-to to get calm<br>(*Such forbidden preparations, he feared a patient that sometimes went and exploited during the leave to get calm.)
144	RCE	Såna där obskyra barer fruktade hon en kriminell som ofta besökte efter frisläppning för att planera nya dåd.<br>Such there obscure bars feared she a criminal that often visited after release in-order-to to plan new attacks<br>(*Such obscure bars, she feared a criminal that often visited after release to plan new attacks.)
144	TCE	Såna där obskyra barer fruktade hon att en kriminell ofta besökte efter frisläppning för att planera nya dåd.<br>Such there obscure bars feared she that a criminal often visited after release in-order-to to plan new attacks<br>(Such obscure bars, she feared that a criminal often visited after release to plan new attacks.)
144	NRCE	Såna där obskyra barer fruktade hon en kriminell som förresten besökte efter frisläppning för att planera nya dåd.<br>Such there obscure bars feared she a criminal that by-the-way visited after release in-order-to to plan new attacks<br>(*Such obscure bars, she feared a criminal that by the way visited after release to plan new attacks.)
144	PCRCE	Såna där obskyra barer fruktade hon en kriminell som ofta kom och besökte efter frisläppning för att planera nya dåd.<br>Such there obscure bars feared she a criminal that often came and visited after release in-order-to to plan new attacks<br>(*Such obscure bars, she feared a criminal that often came and visited after release to plan new attacks.)
145	RCE	Såna här djupa sprickor misstänkte jag en mäklare som alltid mörkade under visningar utan att skämmas.<br>Such here deep cracks suspected I a broker that always covered-up during shows without to skämmas<br>(*Such deep cracks, I suspected a broker that always covered up during shows without shame.)
145	TCE	Såna här djupa sprickor misstänkte jag att en mäklare alltid mörkade under visningar utan att skämmas.<br>Such here deep cracks suspected that I a broker always covered-up during shows without to skämmas<br>(Such deep cracks, I suspected that a broker always covered up during shows without shame.)
145	NRCE	Såna här djupa sprickor misstänkte jag en mäklare som förresten mörkade under visningar utan att skämmas.<br>Such here deep cracks suspected I a broker that by-the-way covered-up during shows without to skämmas<br>(*Such deep cracks, I suspected a broker that by the way covered up during shows without shame.)

145	PCRCE	Såna här djupa sprickor misstänkte jag en mäklare som alltid kom och mörkade under visningar utan att skämmas.<br>Such here deep cracks suspected I a broker that always came and covered-up during shows without to skämmas<br>(Such deep cracks, I suspected a broker that always came and covered up during shows without shame.)
146	RCE	Såna här svårlagade läckage misstänkte vi en reparatör som ofta missade i källarutrymmen på grund av lättja.<br>Such here hard-to-repair leakage suspected we a repairer that often missed in basements on ground of laziness<br>(*Such hard to repair leakages, we suspected a repairer that often missed in basements because of laziness.)
146	TCE	Såna här svårlagade läckage misstänkte vi att en reparatör ofta missade i källarutrymmen på grund av lättja.<br>Such here hard-to-repair leakage suspected we that a repairer often missed in basements on ground of laziness<br>(Such hard to repair leakages, we suspected that a repairer often missed in basements because of laziness.)
146	NRCE	Såna här svårlagade läckage misstänkte vi en reparatör som förresten missade i källarutrymmen på grund av lättja.<br>Such here hard-to-repair leakage suspected we a repairer that by-the-way missed in basements on ground of laziness<br>(*Such hard to repair leakages, we suspected a repairer that by the way missed in basements because of laziness.)
146	PCRCE	Såna här svårlagade läckage misstänkte vi en reparatör som ofta kom och missade i källarutrymmen på grund av lättja.<br>Such here hard-to-repair leakage suspected we a repairer that often came and missed in basements on ground of laziness<br>(Such hard to repair leakages, we suspected a repairer that often came and missed in basements because of laziness.)
147	RCE	Såna där patetiska veckotidningar föraktade hon en arbetskompis som alltid läste på lunchrasten varje onsdag.<br>Such there pathetic magazines disdained she a colleague that always read at lunch-break-the every wednesday<br>(*Such pathetic magazines, she disdained a colleague that always read during the lunch break every wednesday.)
147	TCE	Såna där patetiska veckotidningar föraktade hon att en arbetskompis alltid läste på lunchrasten varje onsdag.<br>Such there pathetic magazines disdained she that a colleague always read at lunch-break-the every wednesday<br>(Such pathetic magazines, she disdained that a colleague always read during the lunch break every wednesday.)
147	NRCE	Såna där patetiska veckotidningar föraktade hon en arbetskompis som förresten läste på lunchrasten varje onsdag.<br>Such there pathetic magazines disdained she a colleague that by-the-way read at lunch-break-the every wednesday<br>(*Such pathetic magazines, she disdained a colleague that by the way

read during the lunch break every wednesday.)

147	PCRCE	Såna där patetiska veckotidningar föraktade hon en arbetskompis som alltid satt och läste på lunchrasten varje onsdag.<br>Such there pathetic magazines disdained she a colleague that always sat and read at lunch-break-the every wednesday<br>(Such pathetic magazines, she disdained a colleague that always sat and read during the lunch break every wednesday.)
148	RCE	Såna där stinkande duschtvålar föraktade han en lagkamrat som alltid beställde på nätet för att spara pengar.<br>Such there stinking shower-soaps disdained he a teammate that always ordered on internet-the in-order-to to save money<br>(Such stinking shower soaps, he disdained a teammate that always ordered online to save money.)
148	TCE	Såna där stinkande duschtvålar föraktade han att en lagkamrat alltid beställde på nätet för att spara pengar.<br>Such there stinking shower-soaps disdained he that a teammate always ordered on internet-the in-order-to to save money<br>(Such stinking shower soaps, he disdained that a team-mate always ordered online to save money.)
148	NRCE	Såna där stinkande duschtvålar föraktade han en lagkamrat som förresten beställde på nätet för att spara pengar.<br>Such there stinking shower-soaps disdained he a teammate that by-the-way ordered on internet-the in-order-to to save money<br>(*Such stinking shower soaps, he disdained a teammate that by the way ordered online to save money.)
148	PCRCE	Såna där stinkande duschtvålar föraktade han en lagkamrat som alltid satt och beställde på nätet för att spara pengar.<br>Such there stinking shower-soaps disdained he a teammate that always sat and ordered on internet-the in-order-to to save money<br>(*Such stinking shower soaps, he disdained a teammate that always sat and ordered online to save money.)
149	RCE	Såna där lustiga ordvitsar värdesatte hon en klient som alltid drog under behandlingen för att roa.<br>Such there funny puns cherished she a client that always made during treatment-the in-order-to to amuse<br>(*Such funny puns, she cherished a client that always made during treatment to amuse.)
149	TCE	Såna där lustiga ordvitsar värdesatte hon att en klient alltid drog under behandlingen för att roa.<br>Such there funny puns cherished she that a client always made during treatment-the in-order-to to amuse<br>(Such funny puns, she cherished that a client always made during treatment to amuse.)
149	NRCE	Såna där lustiga ordvitsar värdesatte hon en klient som förresten drog under behandlingen för att roa.<br>Such there funny puns cherished she a client that by-the-way made during treatment-the in-order-to to amuse<br>(*Such funny puns, she cherished she a client that by the way made

		during treatment to amuse.)
149	PCRCE	Såna där lustiga ordvitsar värdesatte hon en klient som alltid satt och drog under behandlingen för att roa.<br>Such there funny puns cherished she a client that always sat and made during treatment-the in-order-to to amuse<br>(*Such funny puns, she cherished a client that always sat and made during treatment to amuse.)
150	RCE	Såna där eleganta kåpor värdesatte han en präst som alltid bar vid dop eftersom han älskade stil.<br>Such there stylish mantles cherished he a priest that always wore at baptism because he loved style<br>(*Such stylish mantles, he cherished a priest that always wore at baptisms because he loved style.)
150	TCE	Såna där eleganta kåpor värdesatte han att en präst alltid bar vid dop eftersom han älskade stil.<br>Such there stylish mantles cherished he that a priest always wore at baptism because he loved style<br>(Such stylish mantles, he cherished that a priest always wore at baptisms because he loved style.)
150	NRCE	Såna där eleganta kåpor värdesatte han att en präst förresten bar vid dop eftersom han älskade stil.<br>Such there stylish mantles cherished he a priest that by-the-way wore at baptism because he loved style<br>(*Such stylish mantles, he cherished that a priest by the way wore at baptisms because he loved style.)
150	PCRCE	Såna där eleganta kåpor värdesatte han en präst som alltid kom och bar vid dop eftersom han älskade stil.<br>Such there stylish mantles cherished he a priest that always came and wore at baptism because he loved style<br>(*Such stylish mantles, he cherished a priest that always came and wore at baptism because he loved the style.)
151	RCE	Såna där vemodiga psalmer hatade vi en kantor som alltid spelade i kyrkan för att underhålla pensionärerna.<br>Such there melancholic hymns hated we a cantor that always played in church-the in-order-to to entertain senior-citizens-the<br>(*Such melancholic hymns, we hated a cantor that always played in the church to entertain the senior citizens.)
151	TCE	Såna där vemodiga psalmer hatade vi att en kantor alltid spelade i kyrkan för att underhålla pensionärerna.<br>Such there melancholic hymns hated we that a cantor always played in church-the in-order-to to entertain senior-citizens-the<br>(Such melancholic hymns, we hated that a cantor always played in the church to entertain the senior citizens.)
151	NRCE	Såna där vemodiga psalmer hatade vi en kantor som förresten spelade i kyrkan för att underhålla pensionärerna.<br>Such there melancholic hymns hated we a cantor that by-the-way played in church-the in-order-to to entertain senior-citizens-the

(*Such melancholic hymns, we hated a cantor that by the way played in the church to entertain the senior citizens.)

151 PCRCE Såna där vemodiga psalmer hatade vi en kantor som alltid satt och spelade i kyrkan för att underhålla pensionärerna.
Such there melancholic hymns hated we a cantor that always sat and played in church-the in-order-to to entertain senior-citizens-the
(Such melancholic hymns, we hated a cantor that always sat and played in the church to entertain the senior citizens.)

152 RCE Såna där pinsamma segergester hatade jag en fotbollsspelare som alltid gjorde under VM när han gjort mål.
Such there embarrasing victory-gestures hated I a footballer that always did during world-cup when he made goal
(*Such embarrasing victory gestures, I hated a footballer that always did during the world cup when he scored.)

152 TCE Såna där pinsamma segergester hatade jag att en fotbollsspelare alltid gjorde under VM när han gjort mål.
Such there embarrasing victory-gestures hated I that a footballer always did during world-cup when he made goal
(Such embarrasing victory gestures, I hated that a footballer always did during the world cup when he scored.)

152 NRCE Såna där pinsamma segergester hatade jag en fotbollsspelare som förresten gjorde under VM när han gjort mål.
Such there embarrasing victory-gestures hated I a footballer that by-the-way did during world-cup when he made goal
(*Such embarrasing victory gestures, I hated a footballer that by the way did during the world cup when he scored.)

152 PCRCE Såna där pinsamma segergester hatade jag en fotbollsspelare som alltid sprang och gjorde under VM när han gjort mål.
Such there embarrasing victory-gestures hated I a footballer that always ran and did during world-cup when he made goal
(*Such embarrasing victory gestures, I hated a footballer that always ran and did during the world cup when he scored.)

153 RCE Såna där dammiga lager märkte han en lokalvårdare som plikttroget städade varje vecka fast de inte användes.
Such there dusty warehouses noticed he a cleaner that dutifully cleaned every week though they not used-were
(*Such dusty warehouses, he noticed a cleaner that dutifully cleaned every week even though they were not used.)

153 TCE Såna där dammiga lager märkte han att en lokalvårdare plikttroget städade varje vecka fast de inte användes.
Such there dusty warehouses noticed he that a cleaner dutifully cleaned every week though they not used-were
(Such dusty warehouses, he noticed that a cleaner dutifully cleaned every week even though they were not used.)

153 NRCE Såna där dammiga lager märkte han en lokalvårdare som förresten städade varje vecka fast de inte användes.
Such there dusty warehouses noticed he a cleaner that by-the-way cleaned every week though they not used-were

		(*Such dusty warehouses, he noticed a cleaner that by the way cleaned every week even though they were not used.)
153	PCRCE	Såna där dammiga lager märkte han en lokalvårdare som plikttroget kom och städade varje vecka fast de inte användes.<br>Such there dusty warehouses noticed he a cleaner that dutifully came and cleaned every week though they not used-were<br>(Such dusty warehouses, he noticed a cleaner that dutifully came and cleaned every week even though they were not used.)
154	RCE	Såna där underliga läten märkte hon en föreläsare som alltid utstötte på seminarierna när han blev upprörd.<br>Such there strange sounds noticed she a lecturer that always outbursted at seminars-the when he became upset<br>(*Such strange sounds, she noticed a lecturer that always outbursted at the seminars when he became upset.)
154	TCE	Såna där underliga läten märkte hon att en föreläsare alltid utstötte på seminarierna när han blev upprörd.<br>Such there strange sounds noticed she that a lecturer always outbursted at seminars-the when he became upset<br>(Such strange sounds, she noticed that a lecturer always outbursted at the seminars when he became upset.)
154	NRCE	Såna där underliga läten märkte hon en föreläsare som förresten utstötte på seminarierna när han blev upprörd.<br>Such there strange sounds noticed she a lecturer that by-the-way outbursted at seminars-the when he became upset<br>(*Such strange sounds, she noticed a lecturer that by the way outbursted at the seminars when he became upset.)
154	PCRCE	Såna där underliga läten märkte hon en föreläsare som alltid satt och utstötte på seminarierna när han blev upprörd.<br>Such there strange sounds noticed she a lecturer that always sat and outbursted at seminars-the when he became upset<br>(*Such strange sounds, she noticed a lecturer that always sat and outbursted at the seminars when he became upset.)
155	RCE	Såna där långa omvägar noterade vi en chaufför som alltid valde under rusningstid för att undvika köerna.<br>Such there long detours noted we a driver that always selected during rush-hour in-order-to avoid queues-the<br>(*Such long detours, we noted a driver that always selected during rush hour to avoid queues.)
155	TCE	Såna där långa omvägar noterade vi att en chaufför alltid valde under rusningstid för att undvika köerna.<br>Such there long detours noted we that a driver always selected during rush-hour in-order-to avoid queues-the<br>(Such long detours, we noted that a driver always selected during rush hour to avoid queues.)
155	NRCE	Såna där långa omvägar noterade vi en chaufför som förresten valde under rusningstid för att undvika köerna.<br>Such there long detours noted we a driver that by-the-way selected during

rush-hour in-order-to avoid queues-the
(*Such long detours, we noted a driver that by the way selected during rush hour to avoid queues.)

155 PCRCE Såna där långa omvägar noterade vi en chaufför som alltid satt och valde under rusningstid för att undvika köerna.
Such there long detours noted we a driver that always sat and selected during rush-hour in-order-to avoid queues-the
(Such long detours, we noted a driver that always sat and selected during rush hour to avoid queues.)

156 RCE Såna där skabbiga duvor noterade jag en flicka som ofta matade på rasten när de andra lekte.
Such there mangy doves noted I a girl that often fed during break-the while the others played
(*Such mangy doves, I noted a girl that often fed during the break while the others played.)

156 TCE Såna där skabbiga duvor noterade jag att en flicka ofta matade på rasten när de andra lekte.
Such there mangy doves noted I that a girl often fed during break-the while the others played
(Such mangy doves, I noted that a girl often fed during the break while the others played.)

156 NRCE Såna där skabbiga duvor noterade jag en flicka som förresten matade på rasten när de andra lekte.
Such there mangy doves noted I a girl that by-the-way fed during break-the while the others played
(*Such mangy doves, I noted a girl that by the way fed during the break while the others played.)

156 PCRCE Såna där skabbiga duvor noterade jag en flicka som ofta stod och matade på rasten när de andra lekte.
Such there mangy doves noted I a girl that often stood and fed during break-the while the others played
(*Such mangy doves, I noted a girl that often stood and fed during the break while the others played.)

157 RCE Såna här excellenta rapporter prioriterade vi en kandidat som snabbt färdigställde efter intervjun för jobbet.
Such there excellent reports prioritized we a candidate that quickly completed after interview-the for job-the
(*Such excellent reports, we prioritized a candidate that quickly completed after the interview for the job.)

157 TCE Såna här excellenta rapporten prioriterade vi att en kandidat snabbt färdigställde efter intervjun för jobbet.
Such there excellent reports prioritized we that a candidate quickly completed after interview-the for job-the
(*Such excellent reports, we prioritized that a candidate quickly completed after the interview for the job.)

157 NRCE Såna här excellenta rapporten prioriterade vi en kandidat som förresten färdigställde efter intervjun för jobbet.
Such there excellent reports prioritized we a candidate that by-the-way

		completed after interview-the for job-the<br>(*Such excellent reports, we prioritized a candidate that by the way completed after the interview for the job.)
157	PCRCE	Såna här excellenta rapporten prioriterade vi en kandidat som snabbt kom och färdigställde efter intervjun för jobbet.<br>Such there excellent reports prioritized we a candidate that quickly came and completed after interview-the for job-the<br>(*Such excellent reports, we prioritized a candidate that quickly came and completed after the interview for the job.)
158	RCE	Såna där svårlästa skrivelser prioriterade han ett team som kunnigt diskuterade på kontoret härom veckan.<br>Such there hard-to-read letters prioritized he a team that competently discussed at office-the the-other week-the<br>(*Such hard to read letters, he prioritized a team that competently discussed at the office the other week.)
158	TCE	Såna där svårlästa skrivelser prioriterade han att ett team kunnigt diskuterade på kontoret härom veckan.<br>Such there hard-to-read letters prioritized he that a team competently discussed at office-the the-other week-the<br>(Such hard to read letters, he prioritized that a team competently discussed at the office the other week.)
158	NRCE	Såna där svårlästa skrivelser prioriterade han ett team som förresten diskuterade på kontoret härom veckan.<br>Such there hard-to-read letters prioritized he a team that by-the-way discussed at office-the the-other week-the<br>(*Such hard to read letters, he prioritized a team that by the way discussed at the office the other week.)
158	PCRCE	Såna där svårlästa skrivelser prioriterade han ett team som kunnigt kom och diskuterade på kontoret härom veckan.<br>Such there hard-to-read letters prioritized he a team that competently came and discussed at office-the the-other week-the<br>(*Such hard to read letters, he prioritized a team that competently came and discussed in the office the other week.)
159	RCE	Såna här komplicerade danssteg visade hon en 4-åring som snabbt klarade utan felsteg på två lektioner.<br>Such here complex dance-step showed she a 4-year-old that quickly managed without mistakes in two lessons<br>(*Such complex dance steps, she showed a 4-year-old that quickly managed without mistakes in two lessons.)
159	TCE	Såna här komplicerade danssteg visade hon att en 4-åring snabbt klarade utan felsteg på två lektioner.<br>Such here complex dance-step showed she that a 4-year-old quickly managed without mistakes in two lessons<br>(Such complex dance steps, she showed that a 4-year-old quickly managed without mistakes in two lessons.)
159	NRCE	Såna här komplicerade danssteg visade hon en 4-åring som förresten klarade utan felsteg på två lektioner.

		Such here complex dance-step showed she a 4-year-old that by-the-way managed without mistakes in two lessons<br>(*Such complex dance steps, she showed a 4-year-old that by the way managed without mistakes in two lessons.)
159	PCRCE	Såna här komplicerade danssteg visade hon en 4-åring som snabbt kom och klarade utan felsteg på två lektioner.<br>Such here complex dance-step showed she a 4-year-old that quickly came and managed without mistakes in two lessons<br>(Such complex dance steps, she showed a 4-year-old that quickly came and managed without mistakes in two lessons.)
160	RCE	Såna här klassiska monologer visade jag en amatörskådespelare som skickligt framförde på youtube med bravur.<br>Such here classic monologues showed I an amateur-actor that ably performed on youtube with brilliance<br>(*Such classic monologues, I showed an amateur actor that ably performed on youtube brilliantly.)
160	TCE	Såna här klassiska monologer visade jag att en amatörskådespelare skickligt framförde på youtube med bravur.<br>Such here classic monologues showed I that an amateur-actor ably performed on youtube with brilliance<br>(Such classic monologues, I showed that an amateur actor ably performed on youtube brilliantly.)
160	NRCE	Såna här klassiska monologer visade jag en amatörskådespelare som förresten framförde på youtube med bravur.<br>Such here classic monologues showed I an amateur-actor that by-the-way performed on youtube with brilliance<br>(*Such classic monologues, I showed an amateur actor that by the way performed on youtube brilliantly.)
160	PCRCE	Såna här klassiska monologer visade jag en amatörskådespelare som skickligt stod och framförde på youtube med bravur.<br>Such here classic monologues showed I an amateur-actor that ably stood and performed on youtube with brilliance<br>(*Such classic monologues, I showed an amateur actor that ably stood and performed on youtube brilliantly.)
161	RCE	Såna här dramatiska epilepsianfall demonstrerade vi en patienttyp som ofta genomled under natten på sjukhuset.<br>Such here dramatic seizures demonstrated we a patient-type that often suffered during night-the at hostpial-the<br>(*Such dramatic seizures, we demonstrated a patient type that often suffered during the night at the hospital.)
161	TCE	Såna här dramatiska epilepsianfall demonstrerade vi att en patienttyp ofta genomled under natten på sjukhuset.<br>Such here dramatic seizures demonstrated we that a patient-type often suffered during night-the at hostpial-the<br>(Such dramatic seizures, we demonstrated that a patient type often suffered during the night at the hospital.)
161	NRCE	Såna här dramatiska epilepsianfall demonstrerade vi en patienttyp som förresten genomled under natten på sjukhuset.

Such here dramatic seizures demonstrated we a patient-type that by-the-way suffered during night-the at hostpial-the
(*Such dramatic seizures, we demonstrated a patient type that by the way suffered during the night at the hospital.)

161 PCRCE Såna här dramatiska epilepsianfall demonstrerade vi en patienttyp som ofta satt och genomled under natten på sjukhuset.
Such here dramatic seizures demonstrated we a patient-type that often sat and suffered during night-the at hostpial-the
(Such dramatic seizures, we demonstrated a patient type that often sat and suffered during the night at the hospital.)

162 RCE Såna här vidsträckta revir demonstrerade han en katt som ofta märkte under natten innan han kastrerades.
Such here wide territory demonstrated he a cat that often noticed during night-the before be neutered-was
(*Such wide territories, he demonstrated a cat that often noticed during the night before he was neutered.)

162 TCE Såna här vidsträckta revir demonstrerade han att en katt ofta märkte under natten innan han kastrerades.
Such here wide territory demonstrated he that a cat often noticed during night-the before be neutered-was
(Such wide territories, he demonstrated that a cat often noticed during the night before he was neutered.)

162 NRCE Såna här vidsträckta revir demonstrerade han en katt som förresten märkte under natten innan han kastrerades.
Such here wide territory demonstrated he a cat that by-the-way noticed during night-the before be neutered-was
(*Such wide territories, he demonstrated a cat that by the way noticed during the night before he was neutered.)

162 PCRCE Såna här vidsträckta revir demonstrerade han en katt som ofta gick och märkte under natten innan han kastrerades.
Such here wide territory demonstrated he a cat that often went and noticed during night-the before be neutered-was
(Such wide territories demonstrated he a cat that often went and noticed during the night before he was neutered.)

163 RCE Såna där hemska skräckfilmer accepterade vi en tonårstjej som förtvivlat genomlevde på tåget fast det störde.
Such there horrible horror-movies accepted we a teenage-girl that despairingly lived-through on train-the though it disturbed
(*Such horrible horror movies, we accepted a teenage girl that despairingly lived through on the train though it disturbed.)

163 TCE Såna där hemska skräckfilmer accepterade vi att en tonårstjej förtvivlat genomlevde på tåget fast det störde.
Such there horrible horror-movies accepted we that a teenage-girl despairingly lived-through on train-the though it disturbed
(Such horrible horror movies, we accepted that a teenage girl despairingly lived through on the train though it disturbed.)

163 NRCE Såna där hemska skräckfilmer accepterade vi en tonårstjej som förresten

genomlevde på tåget fast det störde.
Such there horrible horror-movies accepted we a teenage-girl that by-the-way lived-through on train-the though it disturbed
(Such horrible horror movies, we accepted a teenage girl that by the way lived through on the train though it disturbed.)

163 PCRCE Såna där hemska skräckfilmer accepterade vi en tonårstjej som förtvivlat satt och genomlevde på tåget fast det störde.
Such there horrible horror-movies accepted we a teenage-girl that despairingly sat and lived-through on train-the though it disturbed
(Such horrible horror movies, we accepted a teenage girl that despairingly sat and lived through on the train though it disturbed.)

164 RCE Såna där otroliga historier accepterade jag en läkare som högljutt reciterade på bussen när jag var på hemväg.
Such there unbelievable stories accepted I a doctor that loudly recited on the bus-the when I was on home-way
(*Such unbelievable stories, I accepted a doctor that loudly recited on the bus when i was on my way home.)

164 TCE Såna där otroliga historier accepterade jag att en läkare högljutt reciterade på bussen när jag var på hemväg.
Such there unbelievable stories accepted I that a doctor loudly recited on the bus-the when I was on home-way
(Such unbelievable stories, I accepted that a doctor loudly recited on the bus when i was on my way home.)

164 NRCE Såna där otroliga historier accepterade jag en läkare som förresten reciterade på bussen när jag var på hemväg.
Such there unbelievable stories accepted I a doctor that by-the-way recited on the bus-the when I was on home-way
(*Such unbelievable stories, I accepted a doctor that by the way recited on the bus when i was on my way home.)

164 PCRCE Såna där otroliga historier accepterade jag en läkare som högljutt satt och reciterade på bussen när jag var på hemväg.
Such there unbelievable stories accepted I a doctor that loudly sat and recited on the bus-the when I was on home-way
(*Such unbelievable stories, I accepted a doctor that loudly sat and recited on the bus when i was on my way home.)

165 RCE Såna där svindyra läppstift avslöjade hon en flicka som motvilligt stal på Åhlens påhejad av sina kompisar.
Such there expensive lipsticks revealed she a girl that reluctantly stole at åhlens cheered-on by her friends
(*Such the expensive lipsticks, she revealed a girl that reluctantly stole at åhlens cheered on by her buddies.)

165 TCE Såna där svindyra läppstift avslöjade hon att en flicka motvilligt stal på Åhlens påhejad av sina kompisar.
Such there expensive lipsticks revealed she that a girl that stole at åhlens cheered-on by her friends
(Such the expensive lipsticks, she revealed that a girl reluctantly stole at åhlens cheered on by his buddies.)

165 NRCE Såna där svindyra läppstift avslöjade hon en flicka som förresten stal på

Åhlens påhejad av sina kompisar.
Such there expensive lipsticks revealed she a girl that by-the-way stole at åhlens cheered-on by her friends
(*Such the expensive lipsticks, she revealed a girl that by the way stole at åhlens cheered on by his buddies.)

165 PCRCE Såna där svindyra läppstift avslöjade hon en flicka som motvilligt kom och stal på Åhlens påhejad av sina kompisar.
Such there expensive lipsticks revealed she a girl that reluctantly came and stole at åhlens cheered-on by her friends
(*Such the expensive lipsticks revealed she a girl that reluctantly came and stole at åhlens cheered on by his buddies.)

166 RCE Såna där hemliga dokument avslöjade han en spion som skickligt smugglade på planet under kalla kriget.
Such there secret document revealed he a spy that skillfully smuggled on plane-the during cold war-the
(*Such secret documents, he revealed a spy that skillfully smuggled on the plane during the cold war.)

166 TCE Såna där hemliga dokument avslöjade han att en spion skickligt smugglade på planet under kalla kriget.
Such there secret document revealed he that a spy skillfully smuggled on plane-the during cold war-the
(Such secret documents, he revealed that a spy skillfully smuggled on the plane during the cold war.)

166 NRCE Såna där hemliga dokument avslöjade han en spion som förresten smugglade på planet under kalla kriget.
Such there secret document revealed he a spy that by-the-way smuggled on plane-the during cold war-the
(*Such secret documents, he revealed a spy that by the way smuggled on the plane during the cold war.)

166 PCRCE Såna där hemliga dokument avslöjade han en spion som skickligt for och smugglade på planet under kalla kriget.
Such there secret document revealed he a spy that skillfully went and smuggled on plane-the during cold war-the
(*Such secret documents, he revealed a spy that skillfully went and smuggled on the plane during the cold war.)

167 RCE Såna här knäppa föreskrifter kritiserade vi ett lag som alltid följde under matcherna för att blidka domaren.
Such here wacky regulations criticized we a team that always followed during matches-the in-order-to to appease judge-the
(*Such wacky regulations, we criticized a team that always followed during matches to appease the judge.)

167 TCE Såna här knäppa föreskrifter kritiserade vi att ett lag alltid följde under matcherna för att blidka domaren.
Such here wacky regulations criticized we that a team always followed during matches-the in-order-to to appease judge-the
(Such wacky regulations, we criticized that a team always followed during matches to appease the judge.)

167	NRCE	Såna här knäppa föreskrifter kritiserade vi ett lag som förresten följde under matcherna för att blidka domaren.<br>Such here wacky regulations criticized we a team that by-the-way followed during matches-the in-order-to to appease judge-the<br>(*Such wacky regulations, we criticized a team that by the way followed during matches to appease the judge.)
167	PCRCE	Såna här knäppa föreskrifter kritiserade vi ett lag som alltid kom och följde under matcherna för att blidka domaren.<br>Such here wacky regulations criticized we a team that always came and followed during matches-the in-order-to to appease judge-the<br>(Such wacky regulations, we criticized a team that always came and followed during matches to appease the judge.)
168	RCE	Såna där tysta vindkraftverk kritiserade jag en militär som illvilligt stoppade på mötet till allas förtret.<br>Such there silent windmills criticized I a military that maliciously stopped at meeting-the to everyone's disappointment<br>(*Such silent windmills, I criticized a military that maliciously stopped at the meeting to everyone's disappointment.)
168	TCE	Såna där tysta vindkraftverk kritiserade jag att en militär illvilligt stoppade på mötet till allas förtret.<br>Such there silent windmills criticized I that a military maliciously stopped at meeting-the to everyone's disappointment<br>(Such silent windmills, I criticized that a military maliciously stopped at the meeting to everyone's disappointment.)
168	NRCE	Såna där tysta vindkraftverk kritiserade jag en militär som förresten stoppade på mötet till allas förtret.<br>Such there silent windmills criticized I a military that by-the-way stopped at meeting-the to everyone's disappointment<br>(*Such silent windmills, I criticized a military that by the way stopped at the meeting to everyone's disappointment.)
168	PCRCE	Såna där tysta vindkraftverk kritiserade jag en militär som illvilligt kom och stoppade på mötet till allas förtret.<br>Such there silent windmills criticized I a military that maliciously came and stopped at meeting-the to everyone's disappointment<br>(*Such silent windmills, I criticized a military that maliciously came and stopped at the meeting to everyone's disappointment.)
169	RCE	Såna där otäcka småkryp uppmärksammade han ett barn som ofta åt i parken efter skolan.<br>Such there nasty bugs drew-attention he a child that often ate in park-the after school-the<br>(*Such nasty bugs, he drew attention a child that often ate in the park after school.)
169	TCE	Såna där otäcka småkryp uppmärksammade han att ett barn ofta åt i parken efter skolan.<br>Such there nasty bugs drew-attention he that a child often ate in park-the after school-the<br>(Such nasty bugs, he drew attention that a child often ate in the park after school.)

169	NRCE	Såna där otäcka småkryp uppmärksammade han ett barn som förresten åt i parken efter skolan.<br>Such there nasty bugs drew-attention he a child that by-the-way ate in park-the after school-the<br>(*Such nasty bugs, he drew attention a child that by the way ate in the park after school.)
169	PCRCE	Såna där otäcka småkryp uppmärksammade han ett barn som ofta kom och åt i parken efter skolan.<br>Such there nasty bugs drew-attention he a child that often came and ate in park-the after school-the<br>(*Such nasty bugs, he drew attention a child that often came and ate in the park after school.)
170	RCE	Såna här krångliga journalsystem uppmärksammade hon en syrra som alltid kraschade med avsikt på jouren.<br>Such here complex medical-records draw-attention she a sister that always crashed with intent on call-the<br>(*Such complex medical records, she drew attention a sister that always crashed with intent on the call.)
170	TCE	Såna här krångliga journalsystem uppmärksammade hon att en syrra alltid kraschade med avsikt på jouren.<br>Such here complex medical-records draw-attention she that a sister always crashed with intent on call-the<br>(Such complex medical records, she drew attention that a sister always crashed with intent on the call.)
170	NRCE	Såna här krångliga journalsystem uppmärksammade hon en syrra som förresten kraschade med avsikt på jouren.<br>Such here complex medical-records draw-attention she a sister that by-the-way crashed with intent on call-the<br>(*Such complex medical records, she drew attention a sister that by the way crashed with intent on the call.)
170	PCRCE	Såna här krångliga journalsystem uppmärksammade hon en syrra som alltid satt och kraschade med avsikt på jouren.<br>Such here complex medical-records draw-attention she a sister that always sat and crashed with intent on call-the<br>(*Such complex medical records, she drew attention a sister that always sat and crashed with intent on the call.)
171	RCE	Såna här underbara skrönor mindes han en piga som alltid berättade på kvällen vid läggdags.<br>Such here wonderful tales remembered he a maid that always told at night-the at bedtime<br>(*Such wonderful tales, he remembered a maid that always told at night at bedtime.)
171	TCE	Såna här underbara skrönor mindes han att en piga allid berättade på kvällen vid läggdags.<br>Such here wonderful tales remembered he that a maid always told at night-the at bedtime<br>(Such wonderful tales, he remembered that a maid extendable frame

always told at night at bedtime.)

171 NRCE Såna här underbara skrönor mindes han en piga som förresten berättade på kvällen vid läggdags.
Such here wonderful tales remembered he a maid that by-the-way told at night-the at bedtime
(*Such wonderful tales, he remembered a maid that by the way told at night at bedtime.)

171 PCRCE Såna här underbara skrönor mindes han en piga som alltid kom och berättade på kvällen vid läggdags.
Such here wonderful tales remembered he a maid that always came and told at night-the at bedtime
(*Such wonderful tales, he remembered a maid that always came and told at night at bedtime.)

172 RCE Såna där melankoliska sånger mindes hon en kille som alltid komponerade i grannrummet när hon bodde på Ålidhem.
Such there melancholic songs remembered she a boy that always composed in neighboring-room-the when she lived at Ålidhem
(*Such melancholic songs, she remembered a boy that always composed in the neighboring room when she lived in ålidhem.)

172 TCE Såna där melankoliska sånger mindes hon att en kille alltid komponerade i grannrummet när hon bodde på Ålidhem.
Such there melancholic songs remembered she that a boy always composed in neighboring-room-the when she lived at Ålidhem
(Such melancholic songs, she remembered that a boy always composed in the neighboring room when she lived in ålidhem.)

172 NRCE Såna där melankoliska sånger mindes hon en kille som förresten komponerade i grannrummet när hon bodde på Ålidhem.
Such there melancholic songs remembered she a boy that by-the-way composed in neighboring-room-the when she lived at Ålidhem
(*Such melancholic songs, she remembered a boy that by the way composed in the neighboring room when she lived in ålidhem.)

172 PCRCE Såna där melankoliska sånger mindes hon en kille som alltid satt och komponerade i grannrummet när hon bodde på Ålidhem.
Such there melancholic songs remembered she a boy that always sat and composed in neighboring-room-the when she lived at Ålidhem
(*Such melancholic songs, she remembered a boy that always sat and composed in the neighboring room when she lived in ålidhem.)

173 RCE Såna där hemgjorda fågelholkar avgudade hon en flickscout som plikttroget krängde i garaget på sommarlovet.
Such there homemade bird-feeders doted she a girl-scout that dutifully sold in garage-the on summer-holiday-the
(*Such homemade bird feeders, she doted a girl scout that dutifully sold in the garage during the summer holiday.)

173 TCE Såna där hemgjorda fågelholkar avgudade hon att en flickscout plikttroget krängde i garaget på sommarlovet.
Such there homemade bird-feeders doted she that a girl-scout dutifully sold in garage-the on summer-holiday-the
(Such homemade bird feeders, she doted that a girl scout dutifully sold in

		the garage during the summer holidays.)
173	NRCE	Såna där hemgjorda fågelholkar avgudade hon en flickscout som förresten krängde i garaget på sommarlovet.<br>Such there homemade bird-feeders doted she a girl-scout that by-the-way sold in garage-the on summer-holiday-the<br>(*Such homemade bird feeders, she doted a girl scout that by the way sold in the garage during the summer holidays.)
173	PCRCE	Såna där hemgjorda fågelholkar avgudade hon en flickscout som plikttroget stod och krängde i garaget på sommarlovet.<br>Such there homemade bird-feeders doted she a girl-scout that dutifully stood and sold in garage-the on summer-holiday-the<br>(*Such homemade bird feeders, she doted a girl scout that dutifully stood and sold in the garage during the summer holidays.)
174	RCE	Såna här färgglada tulpaner avgudade hon en väninna som alltid odlade på kolonilotten under våren.<br>Such here colorful tulips doted she a friend that always cultivated on allotment-the during spring-the<br>(*Such colorful tulips, she doted a friend that always cultivated on the allotment during the spring.)
174	TCE	Såna här färgglada tulpaner avgudade hon att en väninna alltid odlade på kolonilotten under våren.<br>Such here colorful tulips doted she that a friend always cultivated on allotment-the during spring-the<br>(Such colorful tulips, she doted that a friend always cultivated on the allotment during the spring.)
174	NRCE	Såna här färgglada tulpaner avgudade hon en väninna som förresten odlade på kolonilotten under våren.<br>Such here colorful tulips doted she a friend that by-the-way cultivated on allotment-the during spring-the<br>(*Such colorful tulips, she doted a friend that by the way cultivated on the allotment during the spring.)
174	PCRCE	Såna här färgglada tulpaner avgudade hon en väninna som alltid stod och odlade på kolonilotten under våren.<br>Such here colorful tulips doted she a friend that always stood and cultivated on allotment-the during spring-the<br>(*Such colorful tulips, she doted a friend that always stood and cultivated on the allotment in the spring.)
175	RCE	Såna där kloka omdömen högaktade jag en klassförälder som ofta fällde på föräldramötena i trean.<br>Such there wise judgements revered I a class-parent that often uttered at parent-meetings-the in third-grade<br>(*Such wise judgements, I revered a class parent that often uttered at the parent meetings in third grade.)
175	TCE	Såna där kloka omdömen högaktade jag att en klassförälder ofta fällde på föräldramötena i trean.<br>Such there wise judgements revered I that a class-parent often uttered at parent-meetings-the in third-grade

(Such wise judgements, I revered that a class parent often uttered at the parent meetings in third grade.)

175 NRCE Såna där kloka omdömen högaktade jag en klassförälder som förresten fällde på föräldramötena i trean.
Such there wise judgements revered I a class-parent that by-the-way uttered at parent-meetings-the in third-grade
(*Such wise judgements, I revered a class parent that by the way uttered at the parent meetings in third grade.)

175 PCRCE Såna där kloka omdömen högaktade jag en klassförälder som ofta kom och fällde på föräldramötena i trean.
Such there wise judgements revered I a class-parent that often came and uttered at parent-meetings-the in third-grade
(*Such wise judgements, I revered a class parent that often came and uttered at the parent meetings in third grade.)

176 RCE Såna där trevliga sammankomster högaktade vi en familj som alltid organiserade i föreningen varje sommar.
Such there pleasant gatherings revered we a family that always organized in association-the every summer
(*Such pleasant gatherings, we revered a family that always organized in the association every summer.)

176 TCE Såna där trevliga sammankomster högaktade vi att en familj alltid organiserade i föreningen varje sommar.
Such there pleasant gatherings revered we that a family always organized in association-the every summer
(Such pleasant gatherings, we revered that a family always organized in the association every summer.)

176 NRCE Såna där trevliga sammankomster högaktade vi en familj som förresten organiserade i föreningen varje sommar.
Such there pleasant gatherings revered we a family that by-the-way organized in association-the every summer
(*Such pleasant gatherings, we revered a family that by the way organized in the association every summer.)

176 PCRCE Såna där trevliga sammankomster högaktade vi en familj som alltid kom och organiserade i föreningen varje sommar.
Such there pleasant gatherings revered we a family that always came and organized in association-the every summer
(*Such pleasant gatherings, we revered a family that always came and organized in the association every summer.)

177 RCE Såna där grava tabbar negligerade han en praktikant som ofta begick i köket eftersom hon var trevlig.
Such there severe blunders neglected he a trainee that often committed in kitchen-the because she was nice
(*Such severe blunders, he neglected a trainee that often committed in the kitchen because she was nice.)

177 TCE Såna där grava tabbar negligerade han att en praktikant ofta begick i köket eftersom hon var trevlig.
Such there severe blunders neglected he that a trainee often committed in kitchen-the because she was nice

(Such severe blunders, he neglected that a trainee often committed in the kitchen because she was nice.)

177	NRCE	Såna där grava tabbar negligerade han en praktikant som förresten begick i köket eftersom hon var trevlig.<br>Such there severe blunders neglected he a trainee that by-the-way committed in kitchen-the because she was nice<br>(*Such severe blunders, he neglected a trainee that by the way committed in the kitchen because she was nice.)
177	PCRCE	Såna där grava tabbar negligerade han en praktikant som ofta kom och begick i köket eftersom hon var trevlig.<br>Such there severe blunders neglected he a trainee that often came and committed in kitchen-the because she was nice<br>(*Such severe blunders, he neglected a trainee that often came and committed in the kitchen because she was nice.)
178	RCE	Såna där grova förolämpningar negligerade han en granne som alltid vrålade till ungarna när de busade.<br>Such there coarse insults neglected he a neighbor that always yelled at kids-the when they larked-around<br>(*Such coarse insults, he neglected a neighbor that always yelled at the kids when they were larking around.)
178	TCE	Såna där grova förolämpningar negligerade han att en granne alltid vrålade till ungarna när de busade.<br>Such there coarse insults neglected he that a neighbor always yelled at kids-the when they larked-around<br>(Such coarse insults, he neglected that a neighbor always yelled at the kids when they were larking around.)
178	NRCE	Såna där grova förolämpningar negligerade han en granne som förresten vrålade till ungarna när de busade.<br>Such there coarse insults neglected he a neighbor that by-the-way yelled at kids-the when they larked-around<br>(*Such coarse insults, he neglected a neighbor that by the way yelled at the kids when they were larking around)
178	PCRCE	Såna där grova förolämpningar negligerade han en granne som alltid stod och vrålade till ungarna när de busade.<br>Such there coarse insults neglected he a neighbor that always stood and yelled at kids-the when they larked-around<br>(*Such coarse insults, he neglected a neighbor that always stood and yelled at the kids when they were larking around.)
179	RCE	Såna här feta abborrar diggade jag en snubbe som alltid rökte på landet under de ljumma augustikvällarna.<br>Such here fat perches liked I a dude that always smoked on countryside-the during the lukewarm august-evenings-the<br>(*Such fat perches, I liked a dude that always smoked on the countryside during the lukewarm august evenings.)
179	TCE	Såna här feta abborrar diggade jag att en snubbe alltid rökte på landet under de ljumma augustikvällarna.<br>Such here fat perches liked I that a dude always smoked on countryside-

the during the lukewarm august-evenings-the
(Such fat perches, I liked that a dude always smoked on the countryside during the lukewarm august evenings.)

179 NRCE Såna här feta abborrar diggade jag en snubbe som förresten rökte på landet under de ljumma augustikvällarna.
Such here fat perches liked I a dude that by-the-way smoked on countryside-the during the lukewarm august-evenings-the
(*Such fat perches, I liked a dude that by the way smoked on the countryside during the lukewarm august evenings.)

179 PCRCE Såna här feta abborrar diggade jag en snubbe som alltid satt och rökte på landet under de ljumma augustikvällarna.
Such here fat perches liked I a dude that always sat and smoked on countryside-the during the lukewarm august-evenings-the
(*Such fat perches, I liked a dude that always sat and smoked on the countryside during the lukewarm august evenings.)

180 RCE Såna här fräcka gitarrsolon diggade vi en rockstjärna som alltid spelade på festivalerna när vi var unga.
Such here brash guitar-solos liked we a rock-star that always played at festivals-the when we were young
(*Such brash guitar solos, we liked a rock star that always played at the festivals when we were young.)

180 TCE Såna här fräcka gitarrsolon diggade vi att en rockstjärna alltid spelade på festivalerna när vi var unga.
Such here brash guitar-solos liked we that a rock-star always played at festivals-the when we were young
(Such brash guitar solos, we liked that a rock star always played at the festivals when we were young.)

180 NRCE Såna här fräcka gitarrsolon diggade vi en rockstjärna som förresten spelade på festivalerna när vi var unga.
Such here brash guitar-solos liked we a rock-star that by-the-way played at festivals-the when we were young
(*Such brash guitar solos, we liked a rock star that by the way played at the festivals when we were young.)

180 PCRCE Såna här fräcka gitarrsolon diggade vi en rockstjärna som alltid stod och spelade på festivalerna när vi var unga.
Such here brash guitar-solos liked we a rock-star that always stood and played at festivals-the when we were young
(*Such brash guitar solos, we liked a rock star that always stood and played at festivals when we were young.)

Experiment 2: critical items

101 NRCE NotSem Blåa kiosker accepterade de en herre som förresten fastställde inför mötet innan kallelsen skickades.
Blue kiosks accepted they a gentleman that by-the-way established for meeting-the before notice-the sent-was
(*Blue kiosks, they accepted a gentleman that by the way established for

			the meeting before the notice was sent.)
101	NRCE	Sem	Nödvändiga principer accepterade de en herre som förresten fastställde inför mötet innan kallelsen skickades.<br>Necessary principles accepted they a gentleman that by-the-way established for meeting-the before notice-the sent-was<br>(*Necessary principles, they accepted a gentleman that by the way established for the meeting before the notice was sent.)
101	RCE	NotSem	Blåa kiosker accepterade de en herre som noggrant fastställde inför mötet innan kallelsen skickades.<br>Blue kiosks accepted they a gentleman that carefully established for meeting-the before notice-the sent-was<br>(*Blue kiosks, they accepted a gentleman that carefully established for the meeting before the notice was sent.)
101	RCE	Sem	Nödvändiga principer accepterade de en herre som noggrant fastställde inför mötet innan kallelsen skickades.<br>Necessary principles accepted they a gentleman that carefully established for meeting-the before notice-the sent-was<br>(*Necessary principles, they accepted a gentleman that carefully established for the meeting before the notice was sent.)
101	TCE	NotSem	Blåa kiosker accepterade de att en herre noggrant fastställde inför mötet innan kallelsen skickades.<br>Blue kiosks accepted they that a gentleman carefully established for meeting-the before notice-the sent-was<br>(Blue kiosks, they accepted they that a gentleman carefully established for the meeting before the notice was sent.)
101	TCE	Sem	Nödvändiga principer accepterade de att en herre noggrant fastställde inför mötet innan kallelsen skickades.<br>Necessary principles accepted they that a gentleman carefully established for meeting-the before notice-the sent-was<br>(Necessary principles, they accepted that a gentleman carefully established for the meeting before the notice was sent.)
102	NRCE	NotSem	Välgrundade misstankar anmälde hon en kille som förresten snattade i godisaffären när frestelsen blev för stor.<br>Well-founded suspicions reported she a boy that by-the-way purloined in candy-store-the when temptation-the became too big<br>(*Well-founded suspicions, she reported a boy that by the way purloined in the candy store when the temptation was too great.)
102	NRCE	Sem	Dyra chokladkakor anmälde hon en kille som förresten snattade i godisaffären när frestelsen blev för stor.<br>Expensive chocolate-bars reported she a boy that by-the-way purloined in candy-store-the when temptation-the became too big<br>(*Expensive chocolate bars, she reported a boy that by the way purloined in the candy store when the temptation was too great.)
102	RCE	NotSem	Välgrundade misstankar anmälde hon en kille som ofta snattade i godisaffären när frestelsen blev för stor.<br>Well-founded suspicions reported she a boy that often purloined in candy-store-the when temptation-the became too big

			(*Well-founded suspicions, she reported a boy that often purloined in the candy store when the temptation was too great.)
102	RCE	Sem	Dyra chokladkakor anmälde hon en kille som ofta snattade i godisaffären när frestelsen blev för stor.<br>Expensive chocolate-bars suspicions reported she a boy that often purloined in candy-store-the when temptation-the became too big<br>(*Expensive chocolate bars, she reported a boy that often purloined inthe candy store when the temptation was too great.)
102	TCE	NotSem	Välgrundade misstankar anmälde hon att en kille ofta snattade i godisaffären när frestelsen blev för stor.<br>Well-founded suspicions reported she that a boy often purloined in candy-store-the when temptation-the became too big<br>(Well-founded suspicions, she reported that a boy often purloined in the candy store when the temptation was too great.)
102	TCE	Sem	Dyra chokladkakor anmälde hon att en kille ofta snattade i godisaffären när frestelsen blev för stor.<br>Expensive chocolate-bars reported she that a boy often purloined in candy-store-the when temptation-the became too big<br>(Expensive chocolate bars, she reported that a boy often purloined in the candy store when the temptation was too great.)
103	NRCE	NotSem	Oslipade saxar antog hon en person som förresten överdrev utan orsak eftersom betygen var bra.<br>Rough scissors assumed she a person that by-the-way exaggerated without cause because grades-the were good<br>(*Rough scissors, she assumed a person that by the way exaggerated without cause because the grades were good.)
103	NRCE	Sem	Minimala risker antog hon en person som förresten överdrev utan orsak eftersom betygen var bra.<br>Minimal risks assumed she a person that by-the-way exaggerated without cause because grades-the were good<br>(*Minimal risks, she assumed a person that by the way exaggerated without cause because the grades were good.)
103	RCE	NotSem	Oslipade saxar antog hon en person som medvetet överdrev utan orsak eftersom betygen var bra.<br>Rough scissors assumed she a person that consciously exaggerated without cause because grades-the were good<br>(*Rough scissors, she assumed a person that consciously exaggerated without cause because the grades were good.)
103	RCE	Sem	Minimala risker antog hon en person som medvetet överdrev utan orsak eftersom betygen var bra.<br>Minimal risks assumed she a person that consciously exaggerated without cause because grades-the were good<br>(*Minimal risks, she assumed a person that consciously exaggerated without cause because the grades were good.)
103	TCE	NotSem	Oslipade saxar antog hon att en person medvetet överdrev utan orsak eftersom betygen var bra.<br>Rough scissors assumed she that a person consciously exaggerated without cause because grades-the were good

			(Rough scissors, she assumed that a person consciously exaggerated without cause because the grades were good.)
103	TCE	Sem	Minimala risker antog hon att en person medvetet överdrev utan orsak eftersom betygen var bra.<br>Minimal risks assumed she that a person consciously exaggerated without cause because grades-the were good<br>(Minimal risks, she assumed that a person consciously exaggerated without cause because the grades were good.)
104	NRCE	NotSem	Vilda äventyr avskydde han en tant som förresten hackade till salladen då alla var hungriga.<br>Wild adventures loathed he a lady that by-the-way chopped for salad-the when everyone was hungry<br>(*Wild adventures, he loathed a lady that by the way chopped for the salad when everyone was hungry.)
104	NRCE	Sem	Nyskördade morötter avskydde han en tant som förresten hackade till salladen då alla var hungriga.<br>Freshly-harvested carrots loathed he a lady that by-the-way chopped for salad-the when everyone was hungry<br>(*Freshly harvested carrots, he loathed a lady that by the way chopped for the salad when everyone was hungry.)
104	RCE	NotSem	Vilda äventyr avskydde han en tant som jämt hackade till salladen då alla var hungriga.<br>Wild adventures loathed he a lady that always chopped for salad-the when everyone was hungry<br>(*Wild adventures, he loathed a lady that always chopped for the salad when everyone was hungry.)
104	RCE	Sem	Nyskördade morötter avskydde han en tant som jämt hackade till salladen då alla var hungriga.<br>Freshly-harvested carrots loathed he a lady that always chopped for salad-the when everyone was hungry<br>(*Freshly harvested carrots, he loathed a lady that always chopped for the salad when everyone was hungry.)
104	TCE	NotSem	Vilda äventyr avskydde han att en tant jämt hackade till salladen då alla var hungriga.<br>Wild adventures loathed he that a lady always chopped for salad-the when everyone was hungry<br>(Wild adventures, he loathed that a lady always chopped for the salad when everyone was hungry.)
104	TCE	Sem	Nyskördade morötter avskydde han att en tant jämt hackade till salladen då alla var hungriga.<br>Freshly-harvested carrots loathed he that a lady always chopped for salad-the when everyone was hungry<br>(Freshly harvested carrots, he loathed that a lady always chopped for the salad when everyone was hungry.)
105	NRCE	NotSem	Gröna vindruvor avundades hon en vän som förresten avslöjade i förväg fastän det var mot reglerna.<br>Green grapes envied she a friend that by-the-way revealed in advance

			although it was against rules-the<br>(*Green grapes, she envied a friend that by the way revealed in advance even though it was against the rules.)
105	NRCE	Sem	Oväntade svar avundades hon en vän som förresten avslöjade i förväg fastän det var mot reglerna.<br>Unexpected responses, she envied a friend that by-the-way revealed in advance although it was against rules-the<br>(*Unexpected responses, she envied a friend that by the way revealed in advance even though it was against the rules.)
105	RCE	NotSem	Gröna vindruvor avundades hon en vän som medvetet avslöjade i förväg fastän det var mot reglerna.<br>Green grapes envied she a friend that consciously revealed in advance although it was against rules-the<br>(*Green grapes, she envied a friend that consciously revealed in advance even though it was against the rules.)
105	RCE	Sem	Oväntade svar avundades hon en vän som medvetet avslöjade i förväg fastän det var mot reglerna.<br>Unexpected responses envied she a friend that consciously revealed in advance although it was against rules-the<br>(*Unexpected responses, she envied a friend that consciously revealed in advance even though it was against the rules.)
105	TCE	NotSem	Gröna vindruvor avundades hon att en vän medvetet avslöjade i förväg fastän det var mot reglerna.<br>Green grapes envied she that a friend consciously revealed in advance although it was against rules-the<br>(Green grapes, she envied that a friend consciously revealed in advance even though it was against the rules.)
105	TCE	Sem	Oväntade svar avundades hon att en vän medvetet avslöjade i förväg fastän det var mot reglerna.<br>Unexpected responses envied she that a friend consciously revealed in advance although it was against rules-the<br>(Unexpected responses, she envied that a friend consciously revealed in advance even though it was against the rules.)
106	NRCE	NotSem	Varma årstider dolde vi en bekant som förresten rev utan verktyg innan solen gått upp.<br>Hot seasons concealed we an acquaintance that by-the-way tore without tool before sun-the got up<br>(*Hot seasons, we concealed an acquaintance that by the way tore without tools before the sun dawned.)
106	NRCE	Sem	Förfallna murar dolde vi en bekant som förresten rev utan verktyg innan solen gått upp.<br>Dilapidated walls concealed we an acquaintance that by-the-way tore without tool before sun-the got up<br>(*Dilapidated walls, we concealed an acquaintance that by the way tore without tools before the sun dawned.)
106	RCE	NotSem	Varma årstider dolde vi en bekant som maniskt rev utan verktyg innan solen gått upp.<br>Hot seasons concealed we an acquaintance that manically tore without

			tool before sun-the got up<br>(*Hot seasons, we concealed an acquaintance that manically tore without tools before the sun dawned.)
106	RCE	Sem	Förfallna murar dolde vi en bekant som maniskt rev utan verktyg innan solen gått upp.<br>Dilapidated walls concealed we an acquaintance that manically tore without tool before sun-the got up<br>(*Dilapidated walls, we concealed an acquaintance that manically tore without tools before the sun dawned.)
106	TCE	NotSem	Varma årstider dolde vi att en bekant maniskt rev utan verktyg innan solen gått upp.<br>Hot seasons concealed we that an acquaintance manically tore without tool before sun-the got up<br>(Hot seasons, we concealed that an acquaintance manically tore without tools before the sun dawned.)
106	TCE	Sem	Förfallna murar dolde vi att en bekant maniskt rev utan verktyg innan solen gått upp.<br>Dilapidated walls concealed we that an acquaintance manically tore without tool before sun-the got up<br>(Dilapidated walls, we concealed that an acquaintance manically tore without tools before the sun dawned.)
107	NRCE	NotSem	Ovanliga fröer försäkrade jag en kvinna som förresten upprepade utan skamkänslor fast det var onödigt.<br>Unusual seeds insured I a woman that by-the-way repeated without shame though it was unnecessary<br>(*Unusual seeds, I insured a woman that by the way repeated without shame though it was unnecessary.)
107	NRCE	Sem	Vita lögner försäkrade jag en kvinna som förresten upprepade utan skamkänslor fast det var onödigt.<br>White lies insured I a woman that by-the-way repeated without shame though it was unnecessary<br>(*White lies, I insured a woman that by the way repeated without shame though it was unnecessary.)
107	RCE	NotSem	Ovanliga fröer försäkrade jag en kvinna som ideligen upprepade utan skamkänslor fast det var onödigt.<br>Unusual seeds insured I a woman that constantly repeated without shame though it was unnecessary<br>(*Unusual seeds, I insured a woman that constantly repeated without shame though it was unnecessary.)
107	RCE	Sem	Vita lögner försäkrade jag en kvinna som ideligen upprepade utan skamkänslor fast det var onödigt.<br>White lies insured I a woman that constantly repeated without shame though it was unnecessary<br>(*White lies, I insured a woman that constantly repeated without shame though it was unnecessary.)
107	TCE	NotSem	Ovanliga fröer försäkrade jag att en kvinna ideligen upprepade utan skamkänslor fast det var onödigt.

			Unusual seeds insured I that a woman constantly repeated without shame though it was unnecessary<br>(Unusual seeds, I insured that a woman constantly repeated without shame though it was unnecessary.)
107	TCE	Sem	Vita lögner försäkrade jag att en kvinna ideligen upprepade utan skamkänslor fast det var onödigt.<br>White lies insured I that a woman constantly repeated without shame though it was unnecessary<br>(White lies, I insured that a woman constantly repeated without shame though it was unnecessary.)
108	NRCE	NotSem	Mogna plommon begrep vi en kvinna som förresten stängde under julhelgen när handeln gick som bäst.<br>Ripe plums understood we a woman that by-the-way closed during christmas-holidays-the when trade-the went as best<br>(*Ripe plums, we understood a woman that by the way closed during the christmas holidays when trade was at its best.)
108	NRCE	Sem	Kvällsöppna butiker begrep vi en kvinna som förresten stängde under julhelgen när handeln gick som bäst.<br>Evening-open shops understood we a woman that by-the-way closed during christmas-holidays-the when trade-the went as best<br>(*Evening open shops, we understood a woman that by the way closed during the christmas holidays when trade was at its best.)
108	RCE	NotSem	Mogna plommon begrep vi en kvinna som ogärna stängde under julhelgen när handeln gick som bäst.<br>Ripe plums understood we a woman that unwillingly closed during christmas-holidays-the when trade-the went as best<br>(*Ripe plums, we understood a woman that unwillingly closed during the christmas holidays when trade was at its best.)
108	RCE	Sem	Kvällsöppna butiker begrep vi en kvinna som ogärna stängde under julhelgen när handeln gick som bäst.<br>Evening-open shops understood we a woman that unwillingly closed during christmas-holidays-the when trade-the went as best<br>(*Evening open shops, we understood a woman that unwillingly closed during the christmas holidays when trade was at its best.)
108	TCE	NotSem	Mogna plommon begrep vi att en kvinna ogärna stängde under julhelgen när handeln gick som bäst.<br>Ripe plums understood we that a woman unwillingly closed during christmas-holidays-the when trade-the went as best<br>(Ripe plums, we understood that a woman unwillingly closed during the christmas holidays when trade was at its best.)
108	TCE	Sem	Kvällsöppna butiker begrep vi att en kvinna ogärna stängde under julhelgen när handeln gick som bäst.<br>Evening-open shops understood we that a woman unwillingly closed during christmas-holidays-the when trade-the went as best<br>(Evening open shops, we understood that a woman unwillingly closed during the christmas holidays when trade was at its best.)
109	NRCE	NotSem	Märkliga intuitioner bekräftade han en gubbe som förresten drog i affären efter att de hade öppnat.

			Strange intuitions confirmed he an old-man that by-the-way pulled in store-the after that they had opened<br>(*Strange intuitions, he confirmed an old man that by the way pulled in the store after they had opened.)
109	NRCE	Sem	Trasiga vagnar bekräftade han en gubbe som förresten drog i affären efter att de hade öppnat.<br>Broken wagons confirmed he an old-man that by-the-way pulled in store-the after that they had opened<br>(*Broken wagons, he confirmed an old man that by the way pulled in the store after they had opened.)
109	RCE	NotSem	Märkliga intuitioner bekräftade han en gubbe som lojt drog i affären efter att de hade öppnat.<br>Strange intuitions confirmed he an old-man that indolently pulled in store-the after that they had opened<br>(*Strange intuitions, he confirmed an old man that indolently pulled in the store after they had opened.)
109	RCE	Sem	Trasiga vagnar bekräftade han en gubbe som lojt drog i affären efter att de hade öppnat.<br>Broken wagons confirmed he an old-man that indolently pulled in store-the after that they had opened<br>(*Broken wagons, he confirmed an old man that indolently pulled in the store after they had opened.)
109	TCE	NotSem	Märkliga intuitioner bekräftade han att en gubbe lojt drog i affären efter att de hade öppnat.<br>Strange intuitions confirmed he that an old-man indolently pulled in store-the after that they had opened<br>(Strange intuitions, he confirmed that an old man indolently pulled in the store after they had opened.)
109	TCE	Sem	Trasiga vagnar bekräftade han att en gubbe lojt drog i affären efter att de hade öppnat.<br>Broken wagons, he confirmed that an old-man indolently pulled in store-the after that they had opened<br>(Broken wagons, he confirmed that an old man indolently pulled in the store after they had opened.)
110	NRCE	NotSem	Vassa tänder beundrade han en kvinna som förresten komponerade på semestern medan andra badade.<br>Sharp teeth admired he a woman that by-the-way composed on vacation-the while others swam<br>(*Sharp teeth, he admired a woman that by the way composed on vacation while others swam.)
110	NRCE	Sem	Långa stycken beundrade han en kvinna som förresten komponerade på semestern medan andra badade.<br>Long paragraphs admired he a woman that by-the-way composed on vacation-the while others swam<br>(*Long paragraphs, he admired a woman that by the way composed on vacation while others swam.)
110	RCE	NotSem	Vassa tänder beundrade han en kvinna som ofta komponerade på

			semestern medan andra badade.<br>Sharp teeth admired he a woman that often composed on vacation-the while others swam<br>(*Sharp teeth, he admired a woman that often composed on vacation while others swam.)
110	RCE	Sem	Långa stycken beundrade han en kvinna som ofta komponerade på semestern medan andra badade.<br>Long paragraphs admired he a woman that often composed on vacation-the while others swam<br>(*Long paragraphs, he admired a woman that often composed on vacation while others swam.)
110	TCE	NotSem	Vassa tänder beundrade han att en kvinna ofta komponerade på semestern medan andra badade.<br>Sharp teeth admired he that a woman often composed on vacation-the while others swam<br>(Sharp teeth, he admired that a woman often composed on vacation while others swam.)
110	TCE	Sem	Långa stycken beundrade han att en kvinna ofta komponerade på semestern medan andra badade.<br>Long paragraphs admired he that a woman often composed on vacation-the while others swam<br>(Long paragraphs, he admired that a woman often composed on vacation while others swam.)
111	NRCE	NotSem	Olika humör bevittnade jag en granne som förresten flyttade i lokalen innan pjäsen började.<br>Different moods witnessed I a neighbor that by-the-way moved in place-the before play-the began<br>(*Different moods, I witnessed a neighbor that by the way moved in the place before the play began.)
111	NRCE	Sem	Tunga möbler bevittnade jag en granne som förresten flyttade i lokalen innan pjäsen började.<br>Heavy furniture witnessed I a neighbor that by-the-way moved in place-the before play-the began<br>(*Heavy furniture, I witnessed a neighbor that by the way moved in the place before the play began.)
111	RCE	NotSem	Olika humör bevittnade jag en granne som hjälpsamt flyttade i lokalen innan pjäsen började.<br>Different moods witnessed I a neighbor that helpfully moved in place-the before play-the began<br>(*Different moods, I witnessed a neighbor that helpfully moved in the place before the play began.)
111	RCE	Sem	Tunga möbler bevittnade jag en granne som hjälpsamt flyttade i lokalen innan pjäsen började.<br>Heavy furniture witnessed I a neighbor that helpfully moved in place-the before play-the began<br>(*Heavy furniture, I witnessed a neighbor that helpfully moved in the place before the play began.)
111	TCE	NotSem	Olika humör bevittnade jag att en granne hjälpsamt flyttade i lokalen

			innan pjäsen började.<br>Different moods witnessed I that a neighbor helpfully moved in place-the before play-the began<br>(Different moods, I witnessed that a neighbor helpfully moved in the place before the play began.)
111	TCE	Sem	Tunga möbler bevittnade jag att en granne hjälpsamt flyttade i lokalen innan pjäsen började.<br>Heavy furniture witnessed I that a neighbor helpfully moved in place-the before play-the began<br>(Heavy furniture, I witnessed that a neighbor helpfully moved in the place before the play began.)
112	NRCE	NotSem	Onda aningar demonstrerade vi en dam som förresten tvättade med såpa trots att alla protesterade.<br>Bad feelings demonstrated we a lady that by-the-way washed with soap despite that all protested<br>(*Bad feelings, we demonstrated a lady that by the way washed with soap despite all protested.)
112	NRCE	Sem	Tjocka handdukar demonstrerade vi en dam som förresten tvättade med såpa trots att alla protesterade.<br>Thick towels demonstrated we a lady that by-the-way washed with soap despite that all protested<br>(*Thick towels, we demonstrated a lady that by the way washed with soap despite all protests.)
112	RCE	NotSem	Onda aningar demonstrerade vi en dam som entusiastiskt tvättade med såpa trots att alla protesterade.<br>Bad feelings demonstrated we a lady that enthusiastically washed with soap despite that all protested<br>(*Bad feelings, we demonstrated a lady that enthusiastically washed with soap despite all protests.)
112	RCE	Sem	Tjocka handdukar demonstrerade vi en dam som entusiastiskt tvättade med såpa trots att alla protesterade.<br>Thick towels demonstrated we a lady that enthusiastically washed with soap despite that all protested<br>(*Thick towels, we demonstrated a lady that enthusiastically washed with soap despite all protests.)
112	TCE	NotSem	Onda aningar demonstrerade vi att en dam entusiastiskt tvättade med såpa trots att alla protesterade.<br>Bad feelings demonstrated we that a lady enthusiastically washed with soap despite that all protested<br>(Bad feelings, we demonstrated that a lady enthusiastically washed with soap despite all protests.)
112	TCE	Sem	Tjocka handdukar demonstrerade vi att en dam entusiastiskt tvättade med såpa trots att alla protesterade.<br>Thick towels demonstrated we that a lady enthusiastically washed with soap despite that all protested<br>(Thick towels, we demonstrated that a lady enthusiastically washed with soap despite all protests.)

113	NRCE	NotSem	Vegetariska lasagner diskuterade han en granne som förresten orsakade i föreningen fastän stämningen annars var mysig.<br>Vegetarian lasagnas discussed he a neighbor that by-the-way caused in association-the although mood-the otherwise was cozy<br>(*Vegetarian lasagnas, he discussed a neighbor that by the way caused in the association although the mood was otherwise cozy.)
113	NRCE	Sem	Allvarliga konflikter diskuterade han en granne som förresten orsakade i föreningen fastän stämningen annars var mysig.<br>Serious conflicts discussed he a neighbor that by-the-way caused in association-the although mood-the otherwise was cozy<br>(*Serious conflicts, he discussed a neighbor that by the way caused in the association although the mood was otherwise cozy.)
113	RCE	NotSem	Vegetariska lasagner diskuterade han en granne som alltid orsakade i föreningen fastän stämningen annars var mysig.<br>Vegetarian lasagnas discussed he a neighbor that always caused in association-the although mood-the otherwise was cozy<br>(*Vegetarian lasagnas, he discussed a neighbor that always caused in the association although the mood was otherwise cozy.)
113	RCE	Sem	Allvarliga konflikter diskuterade han en granne som alltid orsakade i föreningen fastän stämningen annars var mysig.<br>Serious conflicts discussed he a neighbor that always caused in association-the although mood-the otherwise was cozy<br>(*Serious conflicts, he discussed a neighbor that always caused in the association although the mood was otherwise cozy.)
113	TCE	NotSem	Vegetariska lasagner diskuterade han att en granne alltid orsakade i föreningen fastän stämningen annars var mysig.<br>Vegetarian lasagnas discussed he that a neighbor always caused in association-the although mood-the otherwise was cozy<br>(Vegetarian lasagnas, he discussed that a neighbor always caused in the association although the mood was otherwise cozy.)
113	TCE	Sem	Allvarliga konflikter diskuterade han att en granne alltid orsakade i föreningen fastän stämningen annars var mysig.<br>Serious conflicts discussed he that a neighbor always caused in association-the although mood-the otherwise was cozy<br>(Serious conflicts, he discussed that a neighbor always caused in the association although the mood was otherwise cozy.)
114	NRCE	NotSem	Smala korridorer fann hon en gubbe som förresten fraktade utan tillstånd när läget var brådskande.<br>Narrow corridors found she an old-man that by-the-way transported without permission when situation-the was urgent<br>(*Narrow corridors, she found an old man that by the way transported without permission when the situation was urgent.)
114	NRCE	Sem	Miljöfarliga produkter fann hon en gubbe som förresten fraktade utan tillstånd när läget var brådskande.<br>Hazardous products found she an old-man that by-the-way transported without permission when situation-the was urgent<br>(*Hazardous products, she found an old man that by the way transported without permission when the situation was urgent.)

114	RCE	NotSem	Smala korridorer fann hon en gubbe som ibland fraktade utan tillstånd när läget var brådskande.<br>Narrow corridors found she an old-man that sometimes transported without permission when situation-the was urgent<br>(*Narrow corridors, she found an old man that sometimes transported without permission when the situation was urgent.)
114	RCE	Sem	Miljöfarliga produkter fann hon en gubbe som ibland fraktade utan tillstånd när läget var brådskande.<br>Hazardous products found she an old-man that sometimes transported without permission when situation-the was urgent<br>(*Hazardous products, she found an old man that sometimes transported without permission when the situation was urgent.)
114	TCE	NotSem	Smala korridorer fann hon att en gubbe ibland fraktade utan tillstånd när läget var brådskande.<br>Narrow corridors found she that an old-man sometimes transported without permission when situation-the was urgent<br>(Narrow corridors, she found that an old man sometimes transported without permission when the situation was urgent.)
114	TCE	Sem	Miljöfarliga produkter fann hon att en gubbe ibland fraktade utan tillstånd när läget var brådskande.<br>Hazardous products found she that an old-man sometimes transported without permission when situation-the was urgent<br>(Hazardous products, she found that an old man sometimes transported without permission when the situation was urgent.)
115	NRCE	NotSem	Plötsliga dödsfall fixade jag en farbror som förresten reparerade i garaget för att det fanns plats där.<br>Sudden deaths fixed I an uncle that by-the-way repaired in garage-the because that there was room there<br>(*Sudden deaths, I fixed an uncle that by the way repaired in the garage because there was room there.)
115	NRCE	Sem	Gamla cyklar fixade jag en farbror som förresten reparerade i garaget för att det fanns plats där.<br>Old bicycles fixed I an uncle that by-the-way repaired in garage-the because that there was room there<br>(*Old bicycles, I fixed an uncle that by the way repaired in the garage because there was room there.)
115	RCE	NotSem	Plötsliga dödsfall fixade jag en farbror som ibland reparerade i garaget för att det fanns plats där.<br>Sudden deaths fixed I an uncle that sometimes repaired in garage-the because that there was room there<br>(*Sudden deaths, I fixed an uncle that sometimes repaired in the garage because there was room there.)
115	RCE	Sem	Gamla cyklar fixade jag en farbror som ibland reparerade i garaget för att det fanns plats där.<br>Old bicycles fixed I an uncle that sometimes repaired in garage-the because that there was room there<br>(*Old bicycles, I fixed an uncle that sometimes repaired in the garage

			because there was room there.)
115	TCE	NotSem	Plötsliga dödsfall fixade jag att en farbror ibland reparerade i garaget för att det fanns plats där.<br>Sudden deaths fixed I that an uncle sometimes repaired in garage-the because that there was room there<br>(Sudden deaths, I fixed that an uncle sometimes repaired in the garage because there was room there.)
115	TCE	Sem	Gamla cyklar fixade jag att en farbror ibland reparerade i garaget för att det fanns plats där.<br>Old bicycles fixed I that an uncle sometimes repaired in garage-the because that there was room there<br>(Old bicycles, I fixed that an uncle sometimes repaired in the garage because there was room there.)
116	NRCE	NotSem	Rosa karameller framhävde de en kompis som förresten snickrade mellan tomterna om det inte var dåligt väder.<br>Pink candies highlighted they a buddy that by-the-way crafted between yards-the if it not was bad weather<br>(*Pink candies, they highlighted a buddy that by the way crafted between the yards if it was not bad weather.)
116	NRCE	Sem	Låga staket framhävde de en kompis som förresten snickrade mellan tomterna om det inte var dåligt väder.<br>Low fence highlighted they a buddy that by-the-way crafted between yards-the if it not was bad weather<br>(*Low fences, they highlighted a buddy that by the way crafted between the yards if it was not bad weather.)
116	RCE	NotSem	Rosa karameller framhävde de en kompis som gärna snickrade mellan tomterna om det inte var dåligt väder.<br>Pink candies highlighted they a buddy that gladly crafted between yards-the if it not was bad weather<br>(Pink candies, they highlighted a buddy that gladly crafted between the yards if it was not bad weather.)
116	RCE	Sem	Låga staket framhävde de en kompis som gärna snickrade mellan tomterna om det inte var dåligt väder.<br>Low fences highlighted they a buddy that gladly crafted between yards-the if it not was bad weather<br>(Low fences, they highlighted a buddy that gladly crafted between the yards if it was not bad weather.)
116	TCE	NotSem	Rosa karameller framhävde de att en kompis gärna snickrade mellan tomterna om det inte var dåligt väder.<br>Pink candies highlighted they that a buddy gladly crafted between yards-the if it not was bad weather<br>(Pink candies, they highlighted that a buddy gladly crafted between the yards if it was not bad weather.)
116	TCE	Sem	Låga staket framhävde de att en kompis gärna snickrade mellan tomterna om det inte var dåligt väder.<br>Low fences highlighted they that a buddy gladly crafted between yards-the if it not was bad weather<br>(Low fences, they highlighted that a buddy gladly crafted between the

			yards if it was not bad weather.)
117	NRCE	NotSem	Oändliga sommarlov fruktade han en pojke som förresten byggde i trädgården när skoldagen var slut.<br>Endless summer-holidays feared he a boy that by-the-way built in garden-the when school-day-the was over<br>(*Endless summer holidays, he feared a boy that by the way built in the garden when the school day was over.)
117	NRCE	Sem	Lustiga kojor fruktade han en pojke som förresten byggde i trädgården när skoldagen var slut.<br>Hilarious huts feared he a boy that by-the-way built in garden-the when school-day-the was over<br>(*Hilarious huts, he feared a boy that by the way built in the garden when the school day was over.)
117	RCE	NotSem	Oändliga sommarlov fruktade han en pojke som alltid byggde i trädgården när skoldagen var slut.<br>Endless summer-holidays feared he a boy that always built in garden-the when school-day-the was over<br>(*Endless summer holidays, he feared a boy that always built in the garden when the school day was over.)
117	RCE	Sem	Lustiga kojor fruktade han en pojke som alltid byggde i trädgården när skoldagen var slut.<br>Hilarious huts feared he a boy that always built in garden-the when school-day-the was over<br>(*Hilarious huts, he feared a boy that always built in the garden when the school day was over.)
117	TCE	NotSem	Oändliga sommarlov fruktade han att en pojke alltid byggde i trädgården när skoldagen var slut.<br>Endless summer-holidays feared he that a boy always built in garden-the when school-day-the was over<br>(Endless summer holidays, he feared that a boy always built in the garden when the school day was over.)
117	TCE	Sem	Lustiga kojor fruktade han att en pojke alltid byggde i trädgården när skoldagen var slut.<br>Hilarious huts feared he that a boy always built in garden-the when school-day-the was over<br>(Hilarious huts, he feared that a boy always built in the garden when the school day was over.)
118	NRCE	NotSem	Hemska mardrömmar föraktade vi en tjej som förresten dumpade på parkeringen fast många klagade.<br>Horrible nightmares disdained we a girl that by-the-way dumped on parking-lot-the though many complained<br>(*Horrible nightmares, we disdained a girl that by the way dumped in the parking lot though many complained.)
118	NRCE	Sem	Illaluktande sopor föraktade vi en tjej som förresten dumpade på parkeringen fast många klagade.<br>Smelly garbage disdained we a girl that by-the-way dumped on parking-lot-the though many complained

			(*Smelly garbage, we disdained a girl that by the way dumped in the parking lot though many complained.)
118	RCE	NotSem	Hemska mardrömmar föraktade vi en tjej som ibland dumpade på parkeringen fast många klagade.<br>Horrible nightmares disdained we a girl that sometimes dumped on parking-lot-the though many complained<br>(*Horrible nightmares, we disdained a girl that sometimes dumped in the parking lot though many complained.)
118	RCE	Sem	Illaluktande sopor föraktade vi en tjej som ibland dumpade på parkeringen fast många klagade.<br>Smelly garbage disdained we a girl that sometimes dumped on parking-lot-the though many complained<br>(*Smelly garbage, we disdained a girl that sometimes dumped in the parking lot though many complained.)
118	TCE	NotSem	Hemska mardrömmar föraktade vi att en tjej ibland dumpade på parkeringen fast många klagade.<br>Horrible nightmares disdained we that a girl sometimes dumped on parking-lot-the though many complained<br>(Horrible nightmares, we disdained that a girl sometimes dumped in the parking lot though many complained.)
118	TCE	Sem	Illaluktande sopor föraktade vi att en tjej ibland dumpade på parkeringen fast många klagade.<br>Smelly garbage disdained we that a girl sometimes dumped on parking-lot-the though many complained<br>(Smelly garbage, we disdained that a girl sometimes dumped in the parking lot though many complained.)
119	NRCE	NotSem	Djupa hav föredrog hon en granne som förresten lindrade med tigerbalsam trots att det sved.<br>Deep oceans preferred she a neighbor that by-the-way alleviated with tiger-bal although that it pinched<br>(*Deep oceans, she preferred a neighbor that by the way alleviated with tiger balm although it pinched.)
119	NRCE	Sem	Svåra plågor föredrog hon en granne som förresten lindrade med tigerbalsam trots att det sved.<br>Severe agonies preferred she a neighbor that by-the-way alleviated with tiger-bal although that it pinched<br>(*Severe agonies, she preferred a neighbor that by the way alleviated with tiger balm although it pinched.)
119	RCE	NotSem	Djupa hav föredrog hon en granne som ofta lindrade med tigerbalsam trots att det sved.<br>Deep oceans preferred she a neighbor that often alleviated with tiger-bal although that it pinched<br>(*Deep oceans, she preferred a neighbor that often alleviated with tiger balm although the wood.)
119	RCE	Sem	Svåra plågor föredrog hon en granne som ofta lindrade med tigerbalsam trots att det sved.<br>Severe agonies preferred she a neighbor that often alleviated with tiger-bal although that it pinched

			(*Severe agonies, she preferred a neighbor that often alleviated with tiger balm although it pinched.)
119	TCE	NotSem	Djupa hav föredrog hon att en granne ofta lindrade med tigerbalsam trots att det sved.<br>Deep oceans preferred she that a neighbor often alleviated with tiger-bal although that it pinched<br>(Deep oceans, she preferred that a neighbor often alleviated with tiger balm although the wood.)
119	TCE	Sem	Svåra plågor föredrog hon att en granne ofta lindrade med tigerbalsam trots att det sved.<br>Severe agonies preferred she that a neighbor often alleviated with tiger-bal although that it pinched<br>(Severe agonies, she preferred that a neighbor often alleviated with tiger balm although it pinched.)
120	NRCE	NotSem	Gedigna erfarenheter föreslog de en kille som förresten städade efter träningen när lokalvårdarna hade ledigt.<br>Solid experiences suggested they a boy that by-the-way cleaned after training-the when clearners-the had time-off<br>(*Solid experiences, they suggested a boy that by the way cleaned after the training when the cleaners had time off.)
120	NRCE	Sem	Slitna golv föreslog de en kille som förresten städade efter träningen när lokalvårdarna hade ledigt.<br>Worn floor suggested they a boy that by-the-way cleaned after training-the when clearners-the had time-off<br>(*Worn floors, they suggested a boy that by the way cleaned after the training when the cleaners had time off.)
120	RCE	NotSem	Gedigna erfarenheter föreslog de en kille som dagligen städade efter träningen när lokalvårdarna hade ledigt.<br>Solid experiences suggested they a boy that daily cleaned after training-the when clearners-the had time-off<br>(*Solid experiences, they suggested a boy that daily cleaned after the training when the cleaners had time off.)
120	RCE	Sem	Slitna golv föreslog de en kille som dagligen städade efter träningen när lokalvårdarna hade ledigt.<br>Worn floor suggested they a boy that daily cleaned after training-the when clearners-the had time-off<br>(*Worn floors, they suggested a boy that daily cleaned after the training when the cleaners had time off.)
120	TCE	NotSem	Gedigna erfarenheter föreslog de att en kille dagligen städade efter träningen när lokalvårdarna hade ledigt.<br>Solid experiences suggested they that a boy daily cleaned after training-the when clearners-the had time-off<br>(Solid experiences, they suggested that a boy daily cleaned after the training when the cleaners had time off.)
120	TCE	Sem	Slitna golv föreslog de att en kille dagligen städade efter träningen när lokalvårdarna hade ledigt.<br>Worn floor suggested they that a boy daily cleaned after training-the

			when clearners-the had time-off<br>(Worn floors, they suggested that a boy daily cleaned after the training when the cleaners had time off.)
121	NRCE	NotSem	Informella sammahang förstod hon en pojke som förresten bakade varje morgon för att det doftade ljuvligt.<br>Informal contexts understood she a boy that by-the-way baked every morning because that it smelled pleasant<br>(*Informal contexts, she understood a boy that by the way baked every morning for the pleasant scent.)
121	NRCE	Sem	Ljusa frallor förstod hon en pojke som förresten bakade varje morgon för att det doftade ljuvligt.<br>Light breads understood she a boy that by-the-way baked every morning because that it smelled pleasant<br>(*Light breads, she understood she a boy that by the way baked every morning for the pleasant scent.)
121	RCE	NotSem	Informella sammahang förstod hon en pojke som gladeligen bakade varje morgon för att det doftade ljuvligt.<br>Informal contexts understood she a boy that gladly baked every morning because that it smelled pleasant<br>(*Informal contexts, she understood a boy that gladly baked every morning for the pleasant scent.)
121	RCE	Sem	Ljusa frallor förstod hon en pojke som gladeligen bakade varje morgon för att det doftade ljuvligt.<br>Light breads understood she a boy that gladly baked every morning because that it smelled pleasant<br>(*Light breads, she understood a boy that gladly baked every morning for the pleasant scent.)
121	TCE	NotSem	Informella sammahang förstod hon att en pojke gladeligen bakade varje morgon för att det doftade ljuvligt.<br>Informal contexts understood she that a boy gladly baked every morning because that it smelled pleasant<br>(Informal contexts, she understood that a boy gladly baked every morning for the pleasant scent.)
121	TCE	Sem	Ljusa frallor förstod hon att en pojke gladeligen bakade varje morgon för att det doftade ljuvligt.<br>Light breads understood she that a boy that gladly baked every morning because that it smelled pleasant<br>(Light breads, she understood that a boy gladly baked every morning for the pleasant scent.)
122	NRCE	NotSem	Enorma översvämningar hanterade hon en släkting som förresten sålde på nätet när det behövdes.<br>Huge floods managed she a relative that by-the-way sold on internet-the when it needed-was<br>(*Huge floods, she managed a relative that by the way sold online when it was needed.)
122	NRCE	Sem	Attraktiva fastigheter hanterade hon en släkting som förresten sålde på nätet när det behövdes.<br>Attractive properties managed she a relative that by-the-way sold on

			internet-the when it needed-was<br>(*Attractive properties, she managed a relative that by the way sold online when it was needed.)
122	RCE	NotSem	Enorma översvämningar hanterade hon en släkting som framgångsrikt sålde på nätet när det behövdes.<br>Huge floods managed she a relative that successfully sold on internet-the when it needed-was<br>(*Huge floods, she managed she a relative that successfully sold online when it was needed.)
122	RCE	Sem	Attraktiva fastigheter hanterade hon en släkting som framgångsrikt sålde på nätet när det behövdes.<br>Attractive properties managed she a relative that successfully sold on internet-the when it needed-was<br>(*Attractive properties, she managed she a relative that successfully sold online when it was needed.)
122	TCE	NotSem	Enorma översvämningar hanterade hon att en släkting framgångsrikt sålde på nätet när det behövdes.<br>Huge floods managed she that a relative successfully sold on internet-the when it needed-was<br>(Huge floods, she managed that a relative successfully sold online when it was needed.)
122	TCE	Sem	Attraktiva fastigheter hanterade hon att en släkting framgångsrikt sålde på nätet när det behövdes.<br>Attractive properties managed she that a relative successfully sold on internet-the when it needed-was<br>(Attractive properties, she managed that a relative successfully sold online when it was needed.)
123	NRCE	NotSem	Häftiga orkaner gillade han en herre som förresten tryckte under kriget fast det rådde pappersbrist.<br>Strong hurricanes liked he a gentleman that by-the-way printed during war-the though there was paper-shortage<br>(*Strong hurricanes, he liked a gentleman that by the way printed during the war though there was a paper shortage.)
123	NRCE	Sem	Radikala tidningar gillade han en herre som förresten tryckte under kriget fast det rådde pappersbrist.<br>Radical newspapers liked he a gentleman that by-the-way printed during war-the though there was paper-shortage<br>(*Radical newspapers, he liked a gentleman that by the way printed during the war though there was a paper shortage.)
123	RCE	NotSem	Häftiga orkaner gillade han en herre som regelbundet tryckte under kriget fast det rådde pappersbrist.<br>Strong hurricanes liked he a gentleman that regularly printed during war-the though there was paper-shortage<br>(*Strong hurricanes, he liked a gentleman that regularly printed during the war though there was a paper shortage.)
123	RCE	Sem	Radikala tidningar gillade han en herre som regelbundet tryckte under kriget fast det rådde pappersbrist.

			Radical newspapers liked he a gentleman that regularly printed during war-the though there was paper-shortage<br>(*Radical newspapers, he liked a gentleman that regularly printed during the war though there was a paper shortage.)
123	TCE	NotSem	Häftiga orkaner gillade han att en herre regelbundet tryckte under kriget fast det rådde pappersbrist.<br>Strong hurricanes liked he that a gentleman regularly printed during war-the though there was paper-shortage<br>(Strong hurricanes, he liked that a gentleman regularly printed during the war though there was a paper shortage.)
123	TCE	Sem	Radikala tidningar gillade han att en herre regelbundet tryckte under kriget fast det rådde pappersbrist.<br>Radical newspapers liked he that a gentleman regularly printed during war-the though there was paper-shortage<br>(Radical newspapers, he liked that a gentleman regularly printed during the war though there was a paper shortage.)
124	NRCE	NotSem	Torra eksem glömde jag en man som förresten begärde per telefon då det var genomgång.<br>Dry eczema forgot I a man that by-the-way requested by phone when there was review<br>(*Dry eczemas, I forgot a man that by the way requested by phone when there was a review.)
124	NRCE	Sem	Hemliga upplysningar glömde jag en man som förresten begärde per telefon då det var genomgång.<br>Secret data forgot I a man that by-the-way requested by phone when there was review<br>(*Secret data, I forgot a man that by the way requested by phone when there was a review.)
124	RCE	NotSem	Torra eksem glömde jag en man som högljutt begärde per telefon då det var genomgång.<br>Dry eczema forgot I a man that loudly requested by phone when there was review<br>(*Dry eczemas, I forgot a man that loudly requested by phone when there was a review.)
124	RCE	Sem	Hemliga upplysningar glömde jag en man som högljutt begärde per telefon då det var genomgång.<br>Secret data forgot I a man that loudly requested by phone when there was review<br>(*Secret data, I forgot a man that loudly requested by phone when there was a review.)
124	TCE	NotSem	Torra eksem glömde jag att en man högljutt begärde per telefon då det var genomgång.<br>Dry eczema forgot I that a man loudly requested by phone when there was review<br>(Dry eczemas, I forgot that a man loudly requested by phone when there was a review.)
124	TCE	Sem	Hemliga upplysningar glömde jag att en man högljutt begärde per telefon då det var genomgång.

			Secret data forgot I that a man loudly requested by phone when there was review<br>(Secret data, I forgot that a man loudly requested by phone when there was a review.)
125	NRCE	NotSem	Roliga julmarknader godkände jag en tjej som förresten sparade i byrån även om det var onödigt.<br>Funny christmas-markets approved I a girl that by-the-way saved in chest-the although if it was unnecessary<br>(*Funny christmas markets, I approved a girl that by the way saved in the chest although it was unnecessary.)
125	NRCE	Sem	Handskrivna kvitton godkände jag en tjej som förresten sparade i byrån även om det var onödigt.<br>Handwritten receipts approved I a girl that by-the-way saved in chest-the although if it was unnecessary<br>(*Handwritten receipts, I approved a girl that by the way saved in the chest although it was unnecessary.)
125	RCE	NotSem	Roliga julmarknader godkände jag en tjej som nitiskt sparade i byrån även om det var onödigt.<br>Funny christmas-markets approved I a girl that zealously saved in chest-the although if it was unnecessary<br>(*Funny christmas markets, I approved a girl that zealously saved in the chest although it was unnecessary.)
125	RCE	Sem	Handskrivna kvitton godkände jag en tjej som nitiskt sparade i byrån även om det var onödigt.<br>Handwritten receipts approved I a girl that zealously saved in chest-the although if it was unnecessary<br>(*Handwritten receipts, I approved a girl that zealously saved in the chest although it was unnecessary.)
125	TCE	NotSem	Roliga julmarknader godkände jag att en tjej nitiskt sparade i byrån även om det var onödigt.<br>Funny christmas-markets approved I that a girl zealously saved in chest-the although if it was unnecessary<br>(Funny christmas markets, I approved that a girl zealously saved in the chest although it was unnecessary.)
125	TCE	Sem	Handskrivna kvitton godkände jag att en tjej nitiskt sparade i byrån även om det var onödigt.<br>Handwritten receipts approved I that a girl zealously saved in chest-the although if it was unnecessary<br>(Handwritten receipts, I approved that a girl zealously saved in the chest although it was unnecessary.)
126	NRCE	NotSem	Nybakade pajer hatade hon en person som förresten följde i vardagen eftersom det alltid blev trubbel.<br>Freshly-baked pies hated she a person that by-the-way followed in everyday-life because there always became trouble<br>(*Freshly baked pies, she hated a person that by the way followed in everyday life because there was always trouble.)
126	NRCE	Sem	Invanda mönster hatade hon en person som förresten följde i vardagen

			eftersom det alltid blev trubbel.<br>Habitual patterns hated she a person that by-the-way followed in everyday-life because there always became trouble<br>(*Habitual patterns, she hated a person that by the way followed in everyday life because there was always trouble.)
126	RCE	NotSem	Nybakade pajer hatade hon en person som ogärna följde i vardagen eftersom det alltid blev trubbel.<br>Freshly-baked pies hated she a person that unwillingly followed in everyday-life because there always became trouble<br>(*Freshly baked pies, she hated a person that unwillingly followed in everyday life because there was always trouble.)
126	RCE	Sem	Invanda mönster hatade hon en person som ogärna följde i vardagen eftersom det alltid blev trubbel.<br>Habitual patterns hated she a person that unwillingly followed in everyday-life because there always became trouble<br>(*Habitual patterns, she hated a person that unwillingly followed in everyday life because there was always trouble.)
126	TCE	NotSem	Nybakade pajer hatade hon att en person ogärna följde i vardagen eftersom det alltid blev trubbel.<br>Freshly-baked pies hated she that a person unwillingly followed in everyday-life because there always became trouble<br>(*Freshly baked pies, she hated that a person unwillingly followed in everyday life because there was always trouble.)
126	TCE	Sem	Invanda mönster hatade hon att en person ogärna följde i vardagen eftersom det alltid blev trubbel.<br>Habitual patterns hated she that a person unwillingly followed in everyday-life because there always became trouble<br>(Habitual patterns, she hated that a person unwillingly followed in everyday life because there was always trouble.)
127	NRCE	NotSem	Slingrande stigar hindrade jag en person som förresten lossade i morse fast det var förbjudet.<br>Winding paths prevented I a person that by-the-way loosened in this-morning though it was forbidden<br>(*Winding paths, I prevented a person that by the way loosened this morning though it was forbidden.)
127	NRCE	Sem	Grova rep hindrade jag en person som förresten lossade i morse fast det var förbjudet.<br>Coarse rope prevented I a person that by-the-way loosened in this-morning though it was forbidden<br>(*Coarse ropes, I prevented a person that by the way loosened this morning though it was forbidden.)
127	RCE	NotSem	Slingrande stigar hindrade jag en person som medvetet lossade i morse fast det var förbjudet.<br>Winding paths prevented I a person that consciously loosened in this-morning though it was forbidden<br>(*Winding paths, I prevented a person that consciously loosened this morning though it was forbidden.)
127	RCE	Sem	Grova rep hindrade jag en person som medvetet lossade i morse fast det

			var förbjudet.<br>Coarse rope prevented I a person that consciously loosened in this-morning though it was forbidden<br>(*Coarse ropes, I prevented a person that consciously loosened this morning though it was forbidden.)
127	TCE	NotSem	Slingrande stigar hindrade jag att en person medvetet lossade i morse fast det var förbjudet.<br>Winding paths prevented I that a person consciously loosened in this-morning though it was forbidden<br>(Winding paths, I prevented that a person consciously loosened this morning though it was forbidden.)
127	TCE	Sem	Grova rep hindrade jag att en person medvetet lossade i morse fast det var förbjudet.<br>Coarse ropes prevented I that a person consciously loosened in this-morning though it was forbidden<br>(Coarse ropes, I prevented that a person consciously loosened this morning though it was forbidden.)
128	NRCE	NotSem	Stelnade metaforer hörde jag en kvinna som förresten stekte i stugan när det började skymma.<br>Solidified metaphors heard I a woman that by-the-way fried in cottage-the when it began darken<br>(*Solidified metaphors, I heard a woman that by the way fried in the cottage when it began to get dark.)
128	NRCE	Sem	Nyplockade kantareller hörde jag en kvinna som förresten stekte i stugan när det började skymma.<br>Freshly-picked chanterelles heard I a woman that by-the-way fried in cottage-the when it began darken<br>(*Freshly picked chanterelles, I heard a woman that by the way fried in the cottage when it began to get dark.)
128	RCE	NotSem	Stelnade metaforer hörde jag en kvinna som långsamt stekte i stugan när det började skymma.<br>Solidified metaphors heard I a woman that slowly fried in cottage-the when it began darken<br>(*Solidified metaphors, I heard a woman that slowly fried in the cottage when it began to get dark.)
128	RCE	Sem	Nyplockade kantareller hörde jag en kvinna som långsamt stekte i stugan när det började skymma.<br>Freshly-picked chanterelles heard I a woman that slowly fried in cottage-the when it began darken<br>(*Freshly picked chanterelles, I heard a woman that slowly fried in the cottage when it began to get dark.)
128	TCE	NotSem	Stelnade metaforer hörde jag att en kvinna långsamt stekte i stugan när det började skymma.<br>Solidified metaphors heard I that a woman slowly fried in cottage-the when it began darken<br>(Solidified metaphors, I heard that a woman slowly fried in the cottage when it began to get dark.)

128	TCE	Sem	Nyplockade kantareller hörde jag att en kvinna långsamt stekte i stugan när det började skymma.<br>Freshly-picked chanterelles heard I that a woman slowly fried in cottage-the when it began darken<br>(Freshly picked chanterelles, I heard that a woman slowly fried in the cottage when it began to get dark.)
129	NRCE	NotSem	Formella kriterier iakttog vi en flicka som förresten vattnade varje kväll fastän det regnade.<br>Formal criteria watched we a girl that by-the-way watered every night although it rained<br>(*Formal criteria, we watched a girl that by the way watered every night even though it rained.)
129	NRCE	Sem	Prunkande rabatter iakttog vi en flicka som förresten vattnade varje kväll fastän det regnade.<br>Dazzling flowerbeds watched we a girl that by-the-way watered every night although it rained<br>(*Dazzling flowerbeds, we watched a girl that by the way watered every night even though it rained.)
129	RCE	NotSem	Formella kriterier iakttog vi en flicka som lugnt vattnade varje kväll fastän det regnade.<br>Formal criteria watched we a girl that calmly watered every night although it rained<br>(*Formal criteria, we watched a girl that calmly watered every night even though it rained.)
129	RCE	Sem	Prunkande rabatter iakttog vi en flicka som lugnt vattnade varje kväll fastän det regnade.<br>Dazzling flowerbeds watched we a girl that calmly watered every night although it rained<br>(*Dazzling flowerbeds, we watched a girl that calmly watered every night even though it rained.)
129	TCE	NotSem	Formella kriterier iakttog vi att en flicka lugnt vattnade varje kväll fastän det regnade.<br>Formal criteria watched we that a girl that calmly watered every night although it rained<br>(Formal criteria, we watched that a girl calmly watered every night even though it rained.)
129	TCE	Sem	Prunkande rabatter iakttog vi att en flicka lugnt vattnade varje kväll fastän det regnade.<br>Dazzling flowerbeds watched we that a girl that calmly watered every night although it rained<br>(Dazzling flowerbeds, we watched that a girl calmly watered every night even though it rained.)
130	NRCE	NotSem	Lätta moln kritiserade jag en person som förresten debiterade efter kontorstid trots att det inte var tillåtet.<br>Light clouds criticized I a person that by-the-way charged after office-hours though that it not was allowed<br>(*Light clouds, I criticized a person that by the way charged after-hours even though it was not allowed.)

130	NRCE	Sem	Onödiga besök kritiserade jag en person som förresten debiterade efter kontorstid trots att det inte var tillåtet.<br>Unnecessary visits criticized I a person that by-the-way charged after office-hours though that it not was allowed<br>(*Unnecessary visits, I criticized a person that by the way charged after-hours even though it was not allowed.)
130	RCE	NotSem	Lätta moln kritiserade jag en person som alltid debiterade efter kontorstid trots att det inte var tillåtet.<br>Light clouds criticized I a person that always charged after office-hours though that it not was allowed<br>(*Light clouds, I criticized a person that always charged after-hours even though it was not allowed.)
130	RCE	Sem	Onödiga besök kritiserade jag en person som alltid debiterade efter kontorstid trots att det inte var tillåtet.<br>Unnecessary visits criticized I a person that always charged after office-hours though that it not was allowed<br>(*Unnecessary visits, I criticized a person that always charged after-hours even though it was not allowed.)
130	TCE	NotSem	Lätta moln kritiserade jag att en person alltid debiterade efter kontorstid trots att det inte var tillåtet.<br>Light clouds criticized I that a person always charged after office-hours though that it not was allowed<br>(Light clouds, I criticized that a person always charged after-hours even though it was not allowed.)
130	TCE	Sem	Onödiga besök kritiserade jag att en person alltid debiterade efter kontorstid trots att det inte var tillåtet.<br>Unnecessary visits criticized I that a person always charged after office-hours though that it not was allowed<br>(Unnecessary visits, I criticized that a person always charged after-hours even though it was not allowed.)
131	NRCE	NotSem	Upprörda känslor meddelade vi en man som förresten färdigställde i tid fastän det var sent på eftermiddagen.<br>Agitated emotions announced we a man that by-the-way completed in time although it was late in afternoon-the<br>(*Agitated emotions, we announced a man that by the way completed in time even though it was late in the afternoon
131	NRCE	Sem	Tunna rapporter meddelade vi en man som förresten färdigställde i tid fastän det var sent på eftermiddagen.<br>Thin reports announced we a man that by-the-way completed in time although it was late in afternoon-the<br>(*Thin reports, we announced a man that by the way completed in time even though it was late in the afternoon.)
131	RCE	NotSem	Upprörda känslor meddelade vi en man som aldrig färdigställde i tid fastän det var sent på eftermiddagen.<br>Agitated emotions announced we a man that never completed in time although it was late in afternoon-the<br>(*Agitated emotions, we announced a man that never completed in time

			even though it was late in the afternoon.)
131	RCE	Sem	Tunna rapporter meddelade vi en man som aldrig färdigställde i tid fastän det var sent på eftermiddagen.<br>Thin reports announced we a man that never completed in time although it was late in afternoon-the<br>(*Thin reports, we announced a man that never completed in time even though it was late in the afternoon.)
131	TCE	NotSem	Upprörda känslor meddelade vi att en man aldrig färdigställde i tid fastän det var sent på eftermiddagen.<br>Agitated emotions announced we that a man never completed in time although it was late in afternoon-the<br>(Agitated emotions, we announced that a man never completed in time even though it was late in the afternoon.)
131	TCE	Sem	Tunna rapporter meddelade vi att en man aldrig färdigställde i tid fastän det var sent på eftermiddagen.<br>Thin reports announced we that a man never completed in time although it was late in afternoon-the<br>(Thin reports, we announced that a man never completed in time even though it was late in the afternoon.)
132	NRCE	NotSem	Sällsynta lökar mindes de en släkting som förresten styrde på 1900-talet när många konkurrerade om makten.<br>Rare onions remembered they a relative that by-the-way ruled in 1900-century-the when many competed for power-the<br>(*Rare onions, they remembered a relative that by the way ruled in the 1900s when many were competing for power.)
132	NRCE	Sem	Konservativa partier mindes de en släkting som förresten styrde på 1900-talet när många konkurrerade om makten.<br>Conservative parties remembered they a relative that by-the-way ruled in 1900-century-the when many competed for power-the<br>(*Conservative parties, they remembered a relative that by the way ruled in the 1900s when many were competing for power.)
132	RCE	NotSem	Sällsynta lökar mindes de en släkting som kunnigt styrde på 1900-talet när många konkurrerade om makten.<br>Rare onions remembered they a relative that knowledgeably ruled in 1900-century-the when many competed for power-the<br>(*Rare onions, they remembered a relative that knowledgeably ruled in the 1900s when many were competing for power.)
132	RCE	Sem	Konservativa partier mindes de en släkting som kunnigt styrde på 1900-talet när många konkurrerade om makten.<br>Conservative parties remembered they a relative that knowledgeably ruled in 1900-century-the when many competed for power-the<br>(*Conservative parties, they remembered a relative that knowledgeably ruled in the 1900s when many were competing for power.)
132	TCE	NotSem	Sällsynta lökar mindes de att en släkting kunnigt styrde på 1900-talet när många konkurrerade om makten.<br>Rare onions remembered they that a relative knowledgeably ruled in 1900-century-the when many competed for power-the<br>(Rare onions, they remembered that a relative knowledgeably ruled in the

			1900s when many were competing for power.)
132	TCE	Sem	Konservativa partier mindes de att en släkting kunnigt styrde på 1900-talet när många konkurrerade om makten.<br>Conservative parties remembered they that a relative knowledgeably ruled in 1900-century-the when many competed for power-the<br>(Conservative parties, they remembered that a relative knowledgeably ruled in the 1900s when many were competing for power.)
133	NRCE	NotSem	Fruktansvärda utbrott missade han en dam som förresten reklamerade utan anledning eftersom telefonlinjen låg nere.<br>Terrible outbreaks missed he a lady that by-the-way complained without reason because phone-line lay down<br>(*Terrible outbreaks, he missed a lady that by the way complained for no reason because the phone line was down.)
133	NRCE	Sem	Nyköpta datorer missade han en dam som förresten reklamerade utan anledning eftersom telefonlinjen låg nere.<br>Newly-purchased computers missed he a lady that by-the-way complained without reason because phone-line lay down<br>(*Newly purchased computers, he missed a lady that by the way complained for no reason because the phone line was down.)
133	RCE	NotSem	Fruktansvärda utbrott missade han en dam som nyligen reklamerade utan anledning eftersom telefonlinjen låg nere.<br>Terrible outbreaks missed he a lady that recently complained without reason because phone-line lay down<br>(*Terrible outbreaks, he missed a lady that recently complained for no reason because the phone line was down.)
133	RCE	Sem	Nyköpta datorer missade han en dam som nyligen reklamerade utan anledning eftersom telefonlinjen låg nere.<br>Newly-purchased computers missed he a lady that recently complained without reason because phone-line lay down<br>(*Newly purchased computers, he missed a lady that recently complained for no reason because the phone line was down.)
133	TCE	NotSem	Fruktansvärda utbrott missade han att en dam nyligen reklamerade utan anledning eftersom telefonlinjen låg nere.<br>Terrible outbreaks missed he that a lady recently complained without reason because phone-line lay down<br>(Terrible outbreaks, he missed that a lady recently complained for no reason because the phone line was down.)
133	TCE	Sem	Nyköpta datorer missade han att en dam nyligen reklamerade utan anledning eftersom telefonlinjen låg nere.<br>Newly-purchased computers missed he that a lady recently complained without reason because phone-line lay down<br>(Newly purchased computers, he missed that a lady recently complained for no reason because the phone line was down.)
134	NRCE	NotSem	Öppna famnar misstänkte vi en kille som förresten kopierade på nätterna medan andra låg och sov.<br>Open arms suspected we a boy that by-the-way copied at nights-the while others lay and slept

			(*Open arms, we suspected a boy that by the way copied during nights while others were sleeping.)
134	NRCE	Sem	Amerikanska filmer misstänkte vi en kille som förresten kopierade på nätterna medan andra låg och sov.<br>American films suspected we a boy that by-the-way copied at nights-the while others lay and slept<br>(*American films, we suspected a boy that by the way copied during nights while others were sleeping.)
134	RCE	NotSem	Öppna famnar misstänkte vi en kille som olagligen kopierade på nätterna medan andra låg och sov.<br>Open arms suspected we a boy that illegaly copied at nights-the while others lay and slept<br>(*Open arms, we suspected a boy that illegally copied during nights while others were sleeping.)
134	RCE	Sem	Amerikanska filmer misstänkte vi en kille som olagligen kopierade på nätterna medan andra låg och sov.<br>American films suspected we a boy that illegaly copied at nights-the while others lay and slept<br>(*American films, we suspected a boy that illegally copied during nights while others were sleeping.)
134	TCE	NotSem	Öppna famnar misstänkte vi att en kille olagligen kopierade på nätterna medan andra låg och sov.<br>Open arms suspected we that a boy illegaly copied at nights-the while others lay and slept<br>(Open arms, we suspected that a boy illegally copied during nights while others were sleeping.)
134	TCE	Sem	Amerikanska filmer misstänkte vi att en kille olagligen kopierade på nätterna medan andra låg och sov.<br>American films suspected we that a boy illegaly copied at nights-the while others lay and slept<br>(American films, we suspected that a boy illegally copied during nights while others were sleeping.)
135	NRCE	NotSem	Höga trappor märkte hon en kille som förresten blandade på festerna fast de flesta drack öl.<br>High stairs noticed she a boy that by-the-way mixed at parties-the though the majority drank beer<br>(*High stairs, she noticed a boy that by the way mixed at the parties though the majority drank beer)
135	NRCE	Sem	Starka drinkar märkte hon en kille som förresten blandade på festerna fast det flesta drack öl.<br>Strong drinks noticed she a boy that by-the-way mixed at parties-the though the majority drank beer<br>(*Strong drinks, she noticed a boy that by the way mixed at the parties though the majority drank beer.)
135	RCE	NotSem	Höga trappor märkte hon en kille som alltid blandade på festerna fast de flesta drack öl.<br>High stairs noticed she a boy that always mixed at parties-the though the majority drank beer

			(*High stairs, she noticed a boy that always mixed at the parties though the majority drank beer.)
135	RCE	Sem	Starka drinkar märkte hon en kille som alltid blandade på festerna fast de flesta drack öl.<br>Strong drinks noticed she a boy that always mixed at parties-the though the majority drank beer<br>(*Strong drinks, she noticed a boy that always mixed at the parties though the majority drank beer.)
135	TCE	NotSem	Höga trappor märkte hon att en kille alltid blandade på festerna fast de flesta drack öl.<br>High stairs noticed she that a boy always mixed at parties-the though the majority drank beer<br>(High stairs noticed she that a boy always mixed at the parties though the majority drank beer.)
135	TCE	Sem	Starka drinkar märkte hon att en kille alltid blandade på festerna fast det flesta drack öl.<br>Strong drinks noticed she that a boy always mixed at parties-the though the majority drank beer<br>(Strong drinks, she noticed that a boy always mixed at the parties though the majority drank beer.)
136	NRCE	NotSem	Slappa resårer noterade vi en bekant som förresten odlade på kolonilotten när det var säsong.<br>Lax elastics noted we an acquaintance that by-the-way cultured on allotment-the when it was season<br>(*Lax elastics, we noted an acquaintance that by the way cultured on the allotment when it was the season.)
136	NRCE	Sem	Vackra blommor noterade vi en bekant som förresten odlade på kolonilotten när det var säsong.<br>Beautiful flowers noted we an acquaintance that by-the-way cultured on allotment-the when it was season<br>(*Beautiful flowers, we noted an acquaintance that by the way cultured on the allotment when it was the season.)
136	RCE	NotSem	Slappa resårer noterade vi en bekant som entusiastiskt odlade på kolonilotten när det var säsong.<br>Lax elastics noted we an acquaintance that enthusiastically cultured on allotment-the when it was season<br>(*Lax elastics, we noted an acquaintance that enthusiastically cultured on the allotment when it was the season.)
136	RCE	Sem	Vackra blommor noterade vi en bekant som entusiastiskt odlade på kolonilotten när det var säsong.<br>Beautiful flowers noted we an acquaintance that enthusiastically cultured on allotment-the when it was season<br>(*Beautiful flowers, we noted an acquaintance that enthusiastically cultured on the allotment when it was the season.)
136	TCE	NotSem	Slappa resårer noterade vi att en bekant entusiastiskt odlade på kolonilotten när det var säsong.<br>Lax elastics noted we that an acquaintance enthusiastically cultured on

allotment-the when it was season
(Lax elastics, we noted that an acquaintance enthusiastically cultured onthe allotment when it was the season.)

136 TCE Sem Vackra blommor noterade vi att en bekant entusiastiskt odlade på kolonilotten när det var säsong.
Beautiful flowers noted we that an acquaintance enthusiastically cultured on allotment-the when it was season
(Beautiful flowers, we noted that an acquaintance enthusiastically cultured on the allotment when it was the season.)

137 NRCE NotSem Osjälviska avsikter nämnde vi en kvinna som förresten torkade i tumlaren fastän det drog mycket el.
Selfless intentions mentioned we a woman that by-the-way dried in tumbler-the although it pulled much electricity
(*Selfless intentions, we mentioned a woman that by the way dried in the tumbler although it took a lot of electricity.)

137 NRCE Sem Små kläder nämnde vi en kvinna som förresten torkade i tumlaren fastän det drog mycket el.
Small clothing mentioned we a woman that by-the-way dried in tumbler-the although it pulled much electricity
(*Small clothing, we mentioned a woman that by the way dried in the tumbler although it took a lot of electricity.)

137 RCE NotSem Osjälviska avsikter nämnde vi en kvinna som ofta torkade i tumlaren fastän det drog mycket el.
Selfless intentions mentioned we a woman that often dried in tumbler-the although it pulled much electricity
(*Selfless intentions, we mentioned a woman that often dried in the tumbler although it took a lot of electricity.)

137 RCE Sem Små kläder nämnde vi en kvinna som ofta torkade i tumlaren fastän det drog mycket el.
Small clothing mentioned we a woman that often dried in tumbler-the although it pulled much electricity
(*Small clothing, we mentioned a woman that often dried in the tumbler although it took a lot of electricity.)

137 TCE NotSem Osjälviska avsikter nämnde vi att en kvinna ofta torkade i tumlaren fastän det drog mycket el.
Selfless intentions mentioned we that a woman often dried in tumbler-the although it pulled much electricity
(Selfless intentions, we mentioned that a woman often dried in the tumbler although it took a lot of electricity.)

137 TCE Sem Små kläder nämnde vi att en kvinna ofta torkade i tumlaren fastän det drog mycket el.
Small clothing mentioned we that a woman often dried in tumbler-the although it pulled much electricity
(Small clothing, we mentioned that a woman often dried in the tumbler although it took a lot of electricity.)

138 NRCE NotSem Norrländska skogar observerade de en pojke som förresten smorde med margarin när smöret var slut.
Northern forests observed they a boy that by-the-way anointed with

			margarine when butter-the was finished<br>(*Northern forests, they observed a boy that by the way anointed with margarine when the butter was finished.)
138	NRCE	Sem	Runda ugnsformar observerade de en pojke som förresten smorde med margarin när smöret var slut.<br>Round ovenware observed they a boy that by-the-way anointed with margarine when butter-the was finished<br>(*Round ovenware, they observed a boy that by the way anointed with margarine when the butter was finished.)
138	RCE	NotSem	Norrländska skogar observerade de en pojke som noggrant smorde med margarin när smöret var slut.<br>Northern forests observed they a boy that carefully anointed with margarine when butter-the was finished<br>(*Northern forests, they observed a boy that carefully anointed with margarine when the butter was finished.)
138	RCE	Sem	Runda ugnsformar observerade de en pojke som noggrant smorde med margarin när smöret var slut.<br>Round ovenware observed they a boy that carefully anointed with margarine when butter-the was finished<br>(*Round ovenware, they observed a boy that carefully anointed with margarine when the butter was finished.)
138	TCE	NotSem	Norrländska skogar observerade de att en pojke noggrant smorde med margarin när smöret var slut.<br>Northern forests observed they that a boy carefully anointed with margarine when butter-the was finished<br>(Northern forests, they observed that a boy carefully anointed with margarine when the butter was finished.)
138	TCE	Sem	Runda ugnsformar observerade de att en pojke noggrant smorde med margarin när smöret var slut.<br>Round ovenware observed they that a boy carefully anointed with margarine when butter-the was finished<br>(Round ovenware, they observed they that a boy carefully anointed with margarine when the butter was finished.)
139	NRCE	NotSem	Förfärliga olyckshändelser ogillade de en tant som förresten reserverade på tåget trots att det var folktomt.<br>Horrible accidents disliked they an aunt that by-the-way reserved on train-the though that it was people-empty<br>(*Horrible accidents, they disliked an aunt that by the way reserved on the train even though it was empty of people.)
139	NRCE	Sem	Bra sittplatser ogillade de en tant som förresten reserverade på tåget trots att det var folktomt.<br>Good seating disliked they an aunt that by-the-way reserved on train-the though that it was people-empty<br>(*Good seating, they disliked an aunt that by the way reserved on the train even though it was empty of people.)
139	RCE	NotSem	Förfärliga olyckshändelser ogillade de en tant som ständigt reserverade på tåget trots att det var folktomt.

			Horrible accidents disliked they an aunt that constantly reserved on train-the though that it was people-empty<br>(*Horrible accidents, they disliked an aunt that constantly reserved on the train even though it was empty of people.)
139	RCE	Sem	Bra sittplatser ogillade de en tant som ständigt reserverade på tåget trots att det var folktomt.<br>Good seating disliked they an aunt that constantly reserved on train-the though that it was people-empty<br>(*Good seating, they disliked an aunt that constantly reserved on the train even though it was empty of people.)
139	TCE	NotSem	Förfärliga olyckshändelser ogillade de att en tant ständigt reserverade på tåget trots att det var folktomt.<br>Horrible accidents disliked they that an aunt constantly reserved on train-the though that it was people-empty<br>(Horrible accidents, they disliked that an aunt constantly reserved on the train even though it was empty of people.)
139	TCE	Sem	Bra sittplatser ogillade de att en tant ständigt reserverade på tåget trots att det var folktomt.<br>Good seating disliked they that an aunt constantly reserved on train-the though that it was people-empty<br>(Good seating, they disliked that an aunt constantly reserved on the train even though it was empty of people.)
140	NRCE	NotSem	Våldsamma attacker ordnade han en man som förresten rengjorde för hand fast det tog tid.<br>Violent attacks arranged he a man that by-the-way cleaned by hand though it took time<br>(*Violent attacks, he arranged a man that by the way cleaned by hand though it took time.)
140	NRCE	Sem	Äkta mattor ordnade han en man som förresten rengjorde för hand fast det tog tid.<br>Genuine carpets arranged he a man that by-the-way cleaned by hand though it took time<br>(*Genuine carpets, he arranged a man that by the way cleaned by hand though it took time.)
140	RCE	NotSem	Våldsamma attacker ordnade han en man som noggrant rengjorde för hand fast det tog tid.<br>Violent attacks arranged he a man that carefully cleaned by hand though it took time<br>(*Violent attacks, he arranged a man that carefully cleaned by hand though it took time.)
140	RCE	Sem	Äkta mattor ordnade han en man som noggrant rengjorde för hand fast det tog tid.<br>Genuine carpets arranged he a man that carefully cleaned by hand though it took time<br>(*Genuine carpets, he arranged a man that carefully cleaned by hand though it took time.)
140	TCE	NotSem	Våldsamma attacker ordnade han att en man noggrant rengjorde för hand fast det tog tid.

			Violent attacks arranged he that a man carefully cleaned by hand though it took time<br>(Violent attacks, he arranged that a man carefully cleaned by hand though it took time.)
140	TCE	Sem	Äkta mattor ordnade han att en man noggrant rengjorde för hand fast det tog tid.<br>Genuine carpets arranged he that a man carefully cleaned by hand though it took time<br>(Genuine carpets, he arranged that a man carefully cleaned by hand though it took time.)
141	NRCE	NotSem	Gula löv prioriterade hon en kvinna som förresten avslutade före deadline eftersom det var angeläget.<br>Yellow leaves prioritized she a woman that by-the-way completed before deadline because it was urgent<br>(*Yellow leaves, she prioritized a woman that by the way completed before the deadline because it was urgent.)
141	NRCE	Sem	Brådskande ärenden prioriterade hon en kvinna som förresten avslutade före deadline eftersom det var angeläget.<br>Urgent matters prioritized she a woman that by-the-way completed before deadline because it was urgent<br>(*Urgent matters, she prioritized a woman that by the way completed before the deadline because it was urgent.)
141	RCE	NotSem	Gula löv prioriterade hon en kvinna som alltid avslutade före deadline eftersom det var angeläget.<br>Yellow leaves prioritized she a woman that always completed before deadline because it was urgent<br>(*Yellow leaves, she prioritized a woman that always completed before the deadline because it was urgent.)
141	RCE	Sem	Brådskande ärenden prioriterade hon en kvinna som alltid avslutade före deadline eftersom det var angeläget.<br>Urgent matters prioritized she a woman that always completed before deadline because it was urgent<br>(*Urgent matters, she prioritized a woman that always completed before the deadline because it was urgent.)
141	TCE	NotSem	Gula löv prioriterade hon att en kvinna alltid avslutade före deadline eftersom det var angeläget.<br>Yellow leaves prioritized she that a woman always completed before deadline because it was urgent<br>(Yellow leaves, she prioritized that a woman always completed before the deadline because it was urgent.)
141	TCE	Sem	Brådskande ärenden prioriterade hon att en kvinna alltid avslutade före deadline eftersom det var angeläget.<br>Urgent matters prioritized she that a woman always completed before deadline because it was urgent<br>(Urgent matters, she prioritized that a woman always completed before the deadline because it was urgent.)
142	NRCE	NotSem	Fräcka kupper rapporterade han en man som förresten lånade på banken

			när räntan steg.<br>Shameless coups reported he a man that by-the-way borrowed at bank-the when interest-rate-the rose<br>(*Shameless coups, he reported a man that by the way borrowed at the bank when interest rates rose.)
142	NRCE	Sem	Stora kapital rapporterade han en man som förresten lånade på banken när räntan steg.<br>Large funds reported he a man that by-the-way borrowed at bank-the when interest-rate-the rose<br>(*Large funds, he reported a man that by the way borrowed at the bank when interest rates rose.)
142	RCE	NotSem	Fräcka kupper rapporterade han en man som ogärna lånade på banken när räntan steg.<br>Shameless coups reprted he a man that unwillingly borrowed at bank-the when interest-rate-the rose<br>(*Shameless coups, he reported a man that unwillingly borrowed at the bank when interest rates rose.)
142	RCE	Sem	Stora kapital rapporterade han en man som ogärna lånade på banken när räntan steg.<br>Large funds reported he a man that unwillingly borrowed at bank-the when interest-rate-the rose<br>(*Large funds, he reported a man that unwillingly borrowed at the bank when interest rates rose.)
142	TCE	NotSem	Fräcka kupper rapporterade han att en man ogärna lånade på banken när räntan steg.<br>Shameless coups reported he that a man unwillingly borrowed at bank-the when interest-rate-the rose<br>(Shameless coups, he reported that a man unwillingly borrowed at the bank when interest rates rose.)
142	TCE	Sem	Stora kapital rapporterade han att en man ogärna lånade på banken när räntan steg.<br>Large funds reported he that a man unwillingly borrowed at bank-the when interest-rate-the rose<br>(Large funds he reported that a man unwillingly borrowed at the bank when interest rates rose.)
143	NRCE	NotSem	Farliga strålar rekommenderade vi en kvinna som förresten plockade på marken om det inte haglade.<br>Dangerous rays recommended we a woman that by-the-way picked on ground-the if it not hailed<br>(*Dangerous rays, we recommended a woman that by the way picked on the ground if it did not hail.)
143	NRCE	Sem	Syrliga äpplen rekommenderade vi en kvinna som förresten plockade på marken om det inte haglade.<br>Tart apples recommended we a woman that by-the-way picked on ground-the if it not hailed<br>(*Tart apples, we recommended a woman that by the way picked on the ground if it did not hail.)
143	RCE	NotSem	Farliga strålar recommenderade vi en kvinna som försiktigt plockade på

			marken om det inte haglade.<br>Dangerous rays recommended we a woman that gently picked on ground-the if it not hailed<br>(*Dangerous rays, we recommended a woman that gently picked on the ground if it did not hail.)
143	RCE	Sem	Syrliga äpplen rekommenderade vi en kvinna som försiktigt plockade på marken om det inte haglade.<br>Tart apples recommended we a woman that gently picked on ground-the if it not hailed<br>(Tart apples, we recommended a woman that gently picked on the ground if it did not hail.)
143	TCE	NotSem	Farliga strålar rekommenderade vi att en kvinna försiktigt plockade på marken om det inte haglade.<br>Dangerous rays recommended we that a woman gently picked on ground-the if it not hailed<br>(Dangerous rays, we recommended that a woman gently picked on the ground if it did not hail.)
143	TCE	Sem	Syrliga äpplen rekommenderade vi att en kvinna försiktigt plockade på marken om det inte haglade.<br>Tart apples recommended we that a woman gently picked on ground-the if it not hailed<br>(Tart apples, we recommended that a woman gently picked on the ground if it did not hail.)
144	NRCE	NotSem	Vidsträckta stränder rättfärdigade de en person som förresten sände till fronten om det var tvunget.<br>Expansive beaches justified they a person that by-the-way sent to front-the if it were required<br>(*Expansive beaches, they justified a person that by the way sent to the front if it were required.)
144	NRCE	Sem	Professionella styrkor rättfärdigade de en person som förresten sände till fronten om det var tvunget.<br>Professional forces justified they a person that by-the-way sent to front-the if it were required<br>(*Professional forces, they justified a person that by the way sent to the front if it were required.)
144	RCE	NotSem	Vidsträckta stränder rättfärdigade de en person som motvilligt sände till fronten om det var tvunget.<br>Expansive beaches justified they a person that reluctantly sent to front-the if it were required<br>(*Expansive beaches, they justified a person that reluctantly sent to the front if it were required.)
144	RCE	Sem	Professionella styrkor rättfärdigade de en person som motvilligt sände till fronten om det var tvunget.<br>Professional forces justified they a person that reluctantly sent to front-the if it were required<br>(*Professional forces, they justified a person that reluctantly sent to the front if it were required.)

144	TCE	NotSem	Vidsträckta stränder rättfärdigade de att en person motvilligt sände till fronten om det var tvunget.<br>Expansive beaches justified they that a person reluctantly sent to front-the if it were required<br>(Expansive beaches, they justified that a person reluctantly sent to the front if it were required.)
144	TCE	Sem	Professionella styrkor rättfärdigade de att en person motvilligt sände till fronten om det var tvunget.<br>Professional forces justified they that a person reluctantly sent to front-the if it were required<br>(Professional forces, they justified that a person reluctantly sent to the front if it were required.)
145	NRCE	NotSem	Gälla skrik röjde jag en man som förresten förstörde i området när månen sken.<br>High-pitched cries revealed I a man that by-the-way destroyed in area-the when moon-the shone<br>(*High-pitched cries, I revealed a man that by the way destroyed in the area when the moon shone.)
145	NRCE	Sem	Felparkerade bilar röjde jag en man som förresten förstörde i området när månen sken.<br>Wrongly-parked cars revealed I a man that by-the-way destroyed in area-the when moon-the shone<br>(*Wrongly parked cars, I revealed a man that by the way destroyed in the area when the moon shone.)
145	RCE	NotSem	Gälla skrik röjde jag en man som hämndlystet förstörde i området när månen sken.<br>High-pitched cries revealed I a man that vindictively destroyed in area-the when moon-the shone<br>(*High-pitched cries, I revealed a man that vindictively destroyed in the area when the moon shone.)
145	RCE	Sem	Felparkerade bilar röjde jag en man som hämndlystet förstörde i området när månen sken.<br>Wrongly-parked cars revealed I a man that vindictively destroyed in area-the when moon-the shone<br>(*Wrongly parked cars, I revealed I a man that vindictively destroyed in the area when the moon shone.)
145	TCE	NotSem	Gälla skrik röjde jag att en man hämndlystet förstörde i området när månen sken.<br>High-pitched cries revealed I that a man vindictively destroyed in area-the when moon-the shone<br>(High-pitched cries, I revealed that a man vindictively destroyed in the area when the moon shone.)
145	TCE	Sem	Felparkerade bilar röjde jag att en man hämndlystet förstörde i området när månen sken.<br>Wrongly-parked cars revealed I that a man vindictively destroyed in area-the when moon-the shone<br>(Wrongly parked cars, I revealed that a man vindictively destroyed in the area when the moon shone.)

146	NRCE	NotSem	Sena påminnelse saknade jag en kvinna som förresten räddade mot betalning innan det var försent.<br>Late reminder lacked I a woman that by-the-way saved for payment before it was too-late<br>(*Late reminders, I lacked a woman that by the way saved for payment before it was too late.)
146	NRCE	Sem	Lokala företag saknade jag en kvinna som förresten räddade mot betalning innan det var försent.<br>Local companies lacked I a woman that by-the-way saved for payment before it was too-late<br>(*Local companies, I lacked a woman that by the way saved for payment before it was too late.)
146	RCE	NotSem	Sena påminnelse saknade jag en kvinna som gärna räddade mot betalning innan det var försent.<br>Late reminder lacked I a woman that gladly saved for payment before it was too-late<br>(*Late reminders, I lacked a woman that gladly saved for payment before it was too late.)
146	RCE	Sem	Lokala företag saknade jag en kvinna som gärna räddade mot betalning innan det var försent.<br>Local companies lacked I a woman that gladly saved for payment before it was too-late<br>(*Local companies, I lacked a woman that gladly saved for payment before it was too late.)
146	TCE	NotSem	Sena påminnelse saknade jag att en kvinna gärna räddade mot betalning innan det var försent.<br>Late reminder lacked I that a woman gladly saved for payment before it was too-late<br>(Late reminders, I lacked that a woman gladly saved for payment before it was too late.)
146	TCE	Sem	Lokala företag saknade jag att en kvinna gärna räddade mot betalning innan det var försent.<br>Local companies lacked I that a woman gladly saved for payment before it was too-late<br>(Local companies, I lacked that a woman gladly saved for payment before it was too late.)
147	NRCE	NotSem	Mörka århundraden stoppade de en kille som förresten testade i smyg innan kontroller infördes.<br>Dark centuries stopped they a boy that by-the-way tested in surreptitiously before controls introduced-were<br>(*Dark centuries, they stopped a boy that by the way tested surreptitiously before controls were introduced.)
147	NRCE	Sem	Skumma metoder stoppade de en kille som förresten testade i smyg innan kontroller infördes.<br>Murky practices stopped they a boy that by-the-way tested in surreptitiously before controls introduced-were<br>(*Murky practices, they stopped a boy that by the way tested

			surreptitiously before controls were introduced.)
147	RCE	NotSem	Mörka århundraden stoppade de en kille som ibland testade i smyg innan kontroller infördes.<br>Dark centuries stopped they a boy that sometimes tested in surreptitiously before controls introduced-were<br>(*Dark centuries, they stopped a boy that sometimes tested surreptitiously before controls were introduced.)
147	RCE	Sem	Skumma metoder stoppade de en kille som ibland testade i smyg innan kontroller infördes.<br>Murky practices stopped they a boy that sometimes tested in surreptitiously before controls introduced-were<br>(*Murky practices, they stopped a boy that sometimes tested surreptitiously before controls were introduced.)
147	TCE	NotSem	Mörka århundraden stoppade de att en kille ibland testade i smyg innan kontroller infördes.<br>Dark centuries stopped they that a boy sometimes tested in surreptitiously before controls introduced-were<br>(Dark centuries, they stopped that a boy sometimes tested surreptitiously before controls were introduced.)
147	TCE	Sem	Skumma metoder stoppade de att en kille ibland testade i smyg innan kontroller infördes.<br>Murky practices stopped they that a boy sometimes tested in surreptitiously before controls introduced-were<br>(Murky practices, they stopped that a boy sometimes tested surreptitiously before controls were introduced.)
148	NRCE	NotSem	Svarta pärmar svarade vi en kompis som förresten berättade på fikarasten trots att ingen var intresserad.<br>Black binders responded we a buddy that by-the-way told at coffee-break-the although that no-one was interested<br>(*Black binders, we responded a buddy that by the way told during the coffee break even though no one was interested.)
148	NRCE	Sem	Alarmerande nyheter svarade vi en kompis som förresten berättade på fikarasten trots att ingen var intresserad.<br>Alarming news responded we a buddy that by-the-way told at coffee-break-the although that no-one was interested<br>(*Alarming news, we responded a buddy that by the way told during the coffee break even though no one was interested.)
148	RCE	NotSem	Svarta pärmar svarade vi en kompis som gärna berättade på fikarasten trots att ingen var intresserad.<br>Black binders responded we a buddy that gladly told at coffee-break-the although that no-one was interested<br>(*Black binders, we responded a buddy that gladly told during the coffee break even though no one was interested.)
148	RCE	Sem	Alarmerande nyheter svarade vi en kompis som gärna berättade på fikarasten trots att ingen var intresserad.<br>Alarming news responded we a buddy that gladly told at coffee-break-the although that no-one was interested<br>(*Alarming news, we responded a buddy that gladly told during the

			coffee break even though no one was interested.)
148	TCE	NotSem	Svarta pärmar svarade vi att en kompis gärna berättade på fikarasten trots att ingen var intresserad.<br>Black binders responded we that a buddy that gladly told at coffee-break-the although that no-one was interested<br>(Black binders, we responded that a buddy gladly told during the coffee break even though no one was interested.)
148	TCE	Sem	Alarmerande nyheter svarade vi att en kompis gärna berättade på fikarasten trots att ingen var intresserad.<br>Alarming news responded we that a buddy that gladly told at coffee-break-the although that no-one was interested<br>(Alarming news, we responded that a buddy gladly told during the coffee break even though no one was interested.)
149	NRCE	NotSem	Barnförbjudna dataspel såg han en vän som förresten pressade till frukost medan kaffet puttrade.<br>X-rated computer-games saw he a friend that by-the-way pressed for breakfast while coffee-the simmered<br>(*X-rated computer games, he saw a friend that by the way pressed for breakfast while the coffee simmered.)
149	NRCE	Sem	Saftiga apelsiner såg han en vän som förresten pressade till frukost medan kaffet puttrade.<br>Juicy oranges saw he a friend that by-the-way pressed for breakfast while coffee-the simmered<br>(*Juicy oranges, he saw a friend that by the way pressed for breakfast while the coffee simmered.)
149	RCE	NotSem	Barnförbjudna dataspel såg han en vän som alltid pressade till frukost medan kaffet puttrade.<br>X-rated computer-games saw he a friend that always pressed for breakfast while coffee-the simmered<br>(*X-rated computer games, he saw a friend that always pressed for breakfast while the coffee simmered.)
149	RCE	Sem	Saftiga apelsiner såg han en vän som alltid pressade till frukost medan kaffet puttrade.<br>Juicy oranges saw he a friend that always pressed for breakfast while coffee-the simmered<br>(*Juicy oranges, he saw a friend that always pressed for breakfast while the coffee simmered.)
149	TCE	NotSem	Barnförbjudna dataspel såg han att en vän alltid pressade till frukost medan kaffet puttrade.<br>X-rated computer-games saw he that a friend always pressed for breakfast while coffee-the simmered<br>(X-rated computer games, he saw that a friend always pressed for breakfast while the coffee simmered.)
149	TCE	Sem	Saftiga apelsiner såg han att en vän alltid pressade till frukost medan kaffet puttrade.<br>Juicy oranges saw he that a friend always pressed for breakfast while coffee-the simmered

			(Juicy oranges, he saw that a friend always pressed for breakfast while the coffee simmered.)
150	NRCE	NotSem	Spontana utflykter tolererade de en tjej som förresten klippte på kunderna trots att chefen gnällde.<br>Spontaneous excursions tolerated they a girl that by-the-way cut on customers-the although manager-the whined<br>(*Spontaneous excursions, they tolerated a girl that by the way cut on the customers although the manager whined.)
150	NRCE	Sem	Fina frisyrer tolererade de en tjej som förresten klippte på kunderna trots att chefen gnällde.<br>Fine hairstyles tolerated they a girl that by-the-way cut on customers-the although manager-the whined<br>(*Fine hairstyles, they tolerated a girl that by the way cut on the customers although the manager whined.)
150	RCE	NotSem	Spontana utflykter tolererade de en tjej som gärna klippte på kunderna trots att chefen gnällde.<br>Spontaneous excursions tolerated they a girl that gladly cut on customers-the although manager-the whined<br>(*Spontaneous excursions, they tolerated a girl that gladly cut on the customers although the manager whined.)
150	RCE	Sem	Fina frisyrer tolererade de en tjej som gärna klippte på kunderna trots att chefen gnällde.<br>Fine hairstyles tolerated they a girl that gladly cut on customers-the although manager-the whined<br>(*Fine hairstyles, they tolerated a girl that gladly cut on the customers although the manager whined.)
150	TCE	NotSem	Spontana utflykter tolererade de att en tjej gärna klippte på kunderna trots att chefen gnällde.<br>Spontaneous excursions tolerated they that a girl gladly cut on customers-the although manager-the whined<br>(Spontaneous excursions, they tolerated that a girl gladly cut on the customers although the manager whined.)
150	TCE	Sem	Fina frisyrer tolererade de att en tjej gärna klippte på kunderna trots att chefen gnällde.<br>Fine hairstyles tolerated they that a girl gladly cut on customers-the although manager-the whined<br>(Fine hairstyles, they tolerated that a girl gladly cut on the customers although the manager whined.)
151	NRCE	NotSem	Smutsiga tår trodde hon en herre som förresten besökte i sällskap då det fortfarande var gratis.<br>Dirty toes thought she a gentleman that by-the-way visited in company when it still was free<br>(*Dirty toes, she thought a gentleman that by the way visited in company when it was still free.)
151	NRCE	Sem	Statliga museer trodde hon en herre som förresten besökte i sällskap då det fortfarande var gratis.<br>National museums thought she a gentleman that by-the-way visited in company when it still was free

			(*National museums, she thought a gentleman that by the way visited in company when it was still free.)
151	RCE	NotSem	Smutsiga tår trodde hon en herre som ofta besökte i sällskap då det fortfarande var gratis.<br>Dirty toes thought she a gentleman that often visited in company when it still was free<br>(*Dirty toes, she thought a gentleman that often visited in company when it was still free.)
151	RCE	Sem	Statliga museer trodde hon en herre som ofta besökte i sällskap då det fortfarande var gratis.<br>National museums thought she a gentleman that often visited in company when it still was free<br>(*National museums, she thought a gentleman that often visited in company when it was still free.)
151	TCE	NotSem	Smutsiga tår trodde hon att en herre ofta besökte i sällskap då det fortfarande var gratis.<br>Dirty toes thought she that a gentleman often visited in company when it still was free<br>(Dirty toes, she thought that a gentleman often visited in company when it was still free.)
151	TCE	Sem	Statliga museer trodde hon att en herre ofta besökte i sällskap då det fortfarande var gratis.<br>National museums thought she that a gentleman often visited in company when it still was free<br>(National museums, she thought that a gentleman often visited in company when it was still free.)
152	NRCE	NotSem	Glupska klunkar uppmärksammade jag en bekant som förresten utförde utan skäl fast en del blev förargade.<br>Greedy gupls observed I an acquaintance that by-the-way performed without reaons though a few became angry<br>(*Greedy gulps, I observed an acquaintance that by the way performed without reason though some were angry.)
152	NRCE	Sem	Goda gärningar uppmärksammade jag en bekant som förresten utförde utan skäl fast en del blev förargade.<br>Good dees observed I an acquaintance that by-the-way performed without reaons though a few became angry<br>(*Good deeds, I observed an acquaintance that by the way performed without reason though some were angry.)
152	RCE	NotSem	Glupska klunkar uppmärksammade jag en bekant som ogärna utförde utan skäl fast en del blev förargade.<br>Greedy gupls observed I an acquaintance that unwillingly performed without reaons though a few became angry<br>(*Greedy gulps, I observed an acquaintance that unwillingly performed without reason though some were angry.)
152	RCE	Sem	Goda gärningar uppmärksammade jag en bekant som ogärna utförde utan skäl fast en del blev förargade.<br>Good deeds observed I an acquaintance that unwillingly performed

			without reaons though a few became angry<br>(*Good deeds, I observed an acquaintance that unwillingly performed without reason though some were angry.)
152	TCE	NotSem	Glupska klunkar uppmärksammade jag att en bekant ogärna utförde utan skäl fast en del blev förargade.<br>Greedy gupls observed I that an acquaintance unwillingly performed without reaons though a few became angry<br>(Greedy gulps, I observed that an acquaintance unwillingly performed without reason though some were angry.)
152	TCE	Sem	Goda gärningar uppmärksammade jag att en bekant ogärna utförde utan skäl fast en del blev förargade.<br>Good deeds observed I that an acquaintance unwillingly performed without reaons though a few became angry<br>(Good deeds, I observed that an acquaintance unwillingly performed without reason though some were angry.)
153	NRCE	NotSem	Stukna dukar uppskattade han en person som förresten mosade på tallriken när andra satt och åt.<br>Ironed wipes appreciated he a person that by-the-way mashed on plate-the when other sate and ate<br>(*Ironed wipes, he appreciated a person that by the way mashed on the plate when others sat and ate.)
153	NRCE	Sem	Kokta grönsaker uppskattade han en person som förresten mosade på tallriken när andra satt och åt.<br>Cooked vegetables appreciated he a person that by-the-way mashed on plate-the when other sate and ate<br>(*Cooked vegetables, he appreciated a person that by the way mashed on the plate when others sat and ate.)
153	RCE	NotSem	Stukna dukar uppskattade han en person som aldrig mosade på tallriken när andra satt och åt.<br>Ironed wipes appreciated he a person that never mashed on plate-the when other sate and ate<br>(*Ironed wipes, he appreciated a person that never mashed on the plate when others sat and ate.)
153	RCE	Sem	Kokta grönsaker uppskattade han en person som aldrig mosade på tallriken när andra satt och åt.<br>Cooked vegetables appreciated he a person that never mashed on plate-the when other sate and ate<br>(*Cooked vegetables, he appreciated a person that never mashed on the plate when others sat and ate.)
153	TCE	NotSem	Stukna dukar uppskattade han att en person aldrig mosade på tallriken när andra satt och åt.<br>Ironed wipes appreciated he that a person never mashed on plate-the when other sate and ate<br>(Ironed wipes, he appreciated that a person never mashed on the plate when others sat and ate.)
153	TCE	Sem	Kokta grönsaker uppskattade han att en person aldrig mosade på tallriken när andra satt och åt.<br>Cooked vegetables appreciated he that a person never mashed on plate-

			the when other sate and ate<br>(Cooked vegetables, he appreciated that a person never mashed on the plate when others sat and ate.)
154	NRCE	NotSem	Dolda ingångar upptäckte vi en person som förresten spred på kliniken trots att ledningen hade varnat.<br>Hidden entrances discovered we a person that by-the-way spread at clinic-the though that management-the had warned<br>(*Hidden entrances, we discovered a person that by the way spread at the clinic although the management had warned.)
154	NRCE	Sem	Lömska virus upptäckte vi en person som förresten spred på kliniken trots att ledningen hade varnat.<br>Sneaky viruses discovered we a person that by-the-way spread at clinic-the although that management-the had warned<br>(*Sneaky viruses, we discovered a person that by the way spread at the clinic although the management had warned.)
154	RCE	NotSem	Dolda ingångar upptäckte vi en person som hämndlystet spred på kliniken trots att ledningen hade varnat.<br>Hidden entrances discovered we a person that vindictively spread at clinic-the although that management-the had warned<br>(*Hidden entrances, we discovered a person that vindictively spread at the clinic although the management had warned.)
154	RCE	Sem	Lömska virus upptäckte vi en person som hämndlystet spred på kliniken trots att ledningen hade varnat.<br>Sneaky viruses discovered we a person that by-the-way spread at clinic-the although that management-the had warned<br>(*Sneaky viruses, we discovered a person that vindictively spread at the clinic although the management had warned.)
154	TCE	NotSem	Dolda ingångar upptäckte vi att en person hämndlystet spred på kliniken trots att ledningen hade varnat.<br>Hidden entrances discovered we that a person by-the-way spread at clinic-the although that management-the had warned<br>(Hidden inputs, we discovered that a person vindictively spread at the clinic although the management had warned.)
154	TCE	Sem	Lömska virus upptäckte vi att en person hämndlystet spred på kliniken trots att ledningen hade varnat.<br>Sneaky viruses discovered we that a person by-the-way spread at clinic-the although that management-the had warned<br>(Sneaky viruses, we discovered that a person vindictively spread at the clinic although the management had warned.)
155	NRCE	NotSem	Snöiga berg urskiljde de en person som förresten tappade på stan när det var fullt av människor.<br>Snowy mountains distinguished they a person that by-the-way lost in town when it was full of humans<br>(*Snowy mountains, they distinguished a person that by the way lost in town when it was full of people.)
155	NRCE	Sem	Viktiga nycklar urskiljde de en person som förresten tappade på stan när det var fullt av människor.

			Important keys distinguished they a person that by-the-way lost in town when it was full of humans<br>(*Important keys, they distinguished a person that by the way lost in town when it was full of people.)
155	RCE	NotSem	Snöiga berg urskiljde de en person som ofta tappade på stan när det var fullt av människor.<br>Snowy mountains distinguished they a person that often lost in town when it was full of humans<br>(*Snowy mountains, they distinguished a person that often lost in town when it was full of people.)
155	RCE	Sem	Viktiga nycklar urskiljde de en person som ofta tappade på stan när det var fullt av människor.<br>Important keys distinguished they a person that often lost in town when it was full of humans<br>(*Important keys, they distinguished a person that often lost in town when it was full of people.)
155	TCE	NotSem	Snöiga berg urskiljde de att en person ofta tappade på stan när det var fullt av människor.<br>Snowy mountains distinguished they that a person often lost in town when it was full of humans<br>(Snowy mountains, they distinguished that a person often lost in town when it was full of people.)
155	TCE	Sem	Viktiga nycklar urskiljde de att en person ofta tappade på stan när det var fullt av människor.<br>Important keys distinguished they that a person often lost in town when it was full of humans<br>(Important keys, they distinguished that a person often lost in town when it was full of people.)
156	NRCE	NotSem	Interna strider valde jag en kompis som förresten monterade på nolltid trots att beskrivningen var knapphändig.<br>Internal battles selected I a buddy that by-the-way mounted in no-time although description-the was scanty<br>(*Internal battles, I selected a buddy that by the way mounted in no time although the description was scanty.)
156	NRCE	Sem	Breda hyllor valde jag en kompis som förresten monterade på nolltid trots att beskrivningen var knapphändig.<br>Wide shelves selected I a buddy that by-the-way mounted in no-time although description-the was scanty<br>(*Wide shelves, I selected a buddy that by the way mounted in no time although the description was scanty.)
156	RCE	NotSem	Interna strider valde jag en kompis som högljutt monterade på nolltid trots att beskrivningen var knapphändig.<br>Internal battles selected I a buddy that loudly mounted in no-time although description-the was scanty<br>(*Internal battles, I selected a buddy that loudly mounted in no time although the description was scanty.)
156	RCE	Sem	Breda hyllor valde jag en kompis som högljutt monterade på nolltid trots att beskrivningen var knapphändig.

			Wide shelves selected I a buddy that loudly mounted in no-time although description-the was scanty<br>(*Wide shelves, I selected a buddy that loudly mounted in no time although the description was scanty.)
156	TCE	NotSem	Interna strider valde jag att en kompis högljutt monterade på nolltid trots att beskrivningen var knapphändig.<br>Internal battles selected I that a buddy loudly mounted in no-time although description-the was scanty<br>(Internal battles, I selected that a buddy loudly mounted in no time although the description was scanty.)
156	TCE	Sem	Breda hyllor valde jag att en kompis högljutt monterade på nolltid trots att beskrivningen var knapphändig.<br>Wide shelves selected I that a buddy loudly mounted in no-time although description-the was scanty<br>(Wide shelves, I selected that a buddy loudly mounted in no time although the description was scanty.)
157	NRCE	NotSem	Gruvliga revanscher bedömde han en bekant som förresten målade åt folk när sånt var populärt.<br>Dreaful revenges judged he an acquaintance that by-the-way painted for people when such-things were popular<br>(*Dreadful revenges, he judged an acquaintance that by the way painted for people when such things were popular.)
157	NRCE	Sem	Begagnade dörrar bedömde han en bekant som förresten målade åt folk när sånt var populärt.<br>Used doors judged he an acquaintance that by-the-way painted for people when such-things were popular<br>(*Used doors, he judged an acquaintance that by the way painted to people when things were popular.)
157	RCE	NotSem	Gruvliga revanscher bedömde han en bekant som kunnigt målade åt folk när sånt var populärt.<br>Dreaful revenges judged he an acquaintance that knowledgeably painted for people when such-things were popular<br>(*Dreadful revenges, he judged an acquaintance that knowledgeably painted to people when things were popular.)
157	RCE	Sem	Begagnade dörrar bedömde han en bekant som kunnigt målade åt folk när sånt var populärt.<br>Used doors judged he an acquaintance that knowledgeably painted for people when such-things were popular<br>(*Used doors, he judged an acquaintance that knowledgeably painted to people when things were popular.)
157	TCE	NotSem	Gruvliga revanscher bedömde han att en bekant kunnigt målade åt folk när sånt var populärt.<br>Dreaful revenges judged he that an acquaintance knowledgeably painted for people when such-things were popular<br>(Dreadful revenges, he judged that a known knowledgeably painted to people when things were popular.)
157	TCE	Sem	Begagnade dörrar bedömde han att en bekant kunnigt målade åt folk när

			sånt var populärt.<br>Used doors judged he that an acquaintance knowledgeably painted for people when such-things were popular<br>(Used doors, he judged that a known knowledgeably painted to people when things were popular.)
158	NRCE	NotSem	Blanka speglar visste de en tjej som förresten gräddade till middag fast det dröjde.<br>Blank mirrors knew they a girl that by-the-way baked for dinner though it took time<br>(*Blank mirrors, they knew a girl that by the way baked for dinner though it took time.)
158	NRCE	Sem	Delikata pizzor visste de en tjej som förresten gräddade till middag fast det dröjde.<br>Delicious pizzas knew they a girl that by-the-way baked for dinner though it took time<br>(*Delicious pizzas, they knew a girl that by the way baked for dinner though it took time.)
158	RCE	NotSem	Blanka speglar visste de en tjej som gärna gräddade till middag fast det dröjde.<br>Blank mirrors knew they a girl that gladly baked for dinner though it took time<br>(*Blank mirrors, they knew a girl that gladly baked for dinner though it took time.)
158	RCE	Sem	Delikata pizzor visste de en tjej som gärna gräddade till middag fast det dröjde.<br>Delicious pizzas knew they a girl that gladly baked for dinner though it took time<br>(*Delicious pizzas, they knew a girl that gladly baked for dinner though it took time.)
158	TCE	NotSem	Blanka speglar visste de att en tjej gärna gräddade till middag fast det dröjde.<br>Blank mirrors knew they that a girl gladly baked for dinner though it took time<br>(Blank mirrors, they knew that a girl gladly baked for dinner though it took time.)
158	TCE	Sem	Delikata pizzor visste de att en tjej gärna gräddade till middag fast det dröjde.<br>Delicious pizzas knew they that a girl gladly baked for dinner though it took time<br>(Delicious pizzas, they knew that a girl gladly baked for dinner though it took time.)
159	NRCE	NotSem	Ljumma månader värdesatte vi en granne som förresten sopade på våren innan det blev för soligt.<br>Luke-warm months cherished we a neighbor that by-the-way swept in spring-the before it became too sunny<br>(*Luke-warm months, we cherished a neighbor that by the way swept in the spring before it was too sunny.)
159	NRCE	Sem	Grusiga trottoarer värdesatte vi en granne som förresten sopade på våren

			innan det blev för soligt.<br>Grainy sidewalks cherished we a neighbor that by-the-way swept in spring-the before it became too sunny<br>(*Grainy sidewalks, we cherished a neighbor that by the way swept in the spring before it was too sunny.)
159	RCE	NotSem	Ljumma månader värdesatte vi en granne som alltid sopade på våren innan det blev för soligt.<br>Luke-warm months cherished we a neighbor that always swept in spring-the before it became too sunny<br>(*Luke-warm months, we cherished a neighbor that always swept in the spring before it was too sunny.)
159	RCE	Sem	Grusiga trottoarer värdesatte vi en granne som alltid sopade på våren innan det blev för soligt.<br>Grainy sidewalks cherished we a neighbor that always swept in spring-the before it became too sunny<br>(*Grainy sidewalks, we cherished a neighbor that always swept in the spring before it was too sunny.)
159	TCE	NotSem	Ljumma månader värdesatte vi att en granne alltid sopade på våren innan det blev för soligt.<br>Luke-warm months cherished we that a neighbor always swept in spring-the before it became too sunny<br>(Luke-warm months, we cherished that a neighbor always swept in the spring before it was too sunny.)
159	TCE	Sem	Grusiga trottoarer värdesatte vi att en granne alltid sopade på våren innan det blev för soligt.<br>Grainy sidewalks cherished we that a neighbor always swept in spring-the before it became too sunny<br>(Grainy sidewalks, we cherished that a neighbor always swept in the spring before it was too sunny.)
160	NRCE	NotSem	Giftiga diskmedel älskade han en släkting som förresten renoverade på fritiden när det var högkonjunktur.<br>Toxic detergent loved he a relative that by-the-way renovated in spare-time-the when it was booming<br>(*Toxic detergent, he loved a relative that by the way renovated in his spare time when it was booming.)
160	NRCE	Sem	Antika byggnader älskade han en släkting som förresten renoverade på fritiden när det var högkonjunktur.<br>Ancient buildings loved he a relative that by-the-way renovated in spare-time-the when it was booming<br>(*Ancient buildings, he loved a relative that by the way renovated in his spare time when it was booming.)
160	RCE	NotSem	Giftiga diskmedel älskade han en släkting som ständigt renoverade på fritiden när det var högkonjunktur.<br>Toxic detergent loved he a relative that constantly renovated in spare-time-the when it was booming<br>(*Toxic detergent, he loved a relative that constantly renovated in his spare time when it was booming.)

160	RCE	Sem	Antika byggnader älskade han en släkting som ständigt renoverade på fritiden när det var högkonjunktur.<br>Ancient buildings loved he a relative that constantly renovated in spare-time-the when it was booming<br>(*Ancient buildings, he loved a relative that constantly renovated in his spare time when it was booming.)
160	TCE	NotSem	Giftiga diskmedel älskade han att en släkting ständigt renoverade på fritiden när det var högkonjunktur.<br>Toxic detergent loved he that a relative constantly renovated in spare-time-the when it was booming<br>(Toxic detergent, he loved that a relative constantly renovated in his spare time when it was booming.)
160	TCE	Sem	Antika byggnader älskade han att en släkting ständigt renoverade på fritiden när det var högkonjunktur.<br>Ancient buildings loved he that a relative constantly renovated in spare-time-the when it was booming<br>(Ancient buildings, he loved that a relative constantly renovated in his spare time when it was booming.)

Experiment 3: critical items

101	RCE	Sem	Såna avancerade volter tillät hon en kille som tränade nästan dagligen att göra till avslutningen i torsdags.<br>Such advanced somersaults allowed she a boy that trained almost daily to do for finish-the in thursday<br>(*Such advanced somersaults, she allowed a boy that trained almost daily to do for the finish last thursday.)
101	RCE	NotSem	Såna avancerade pajer tillät hon en kille som tränade nästan dagligen att göra till avslutningen i torsdags.<br>Such advanced pies allowed she a boy that trained almost daily to do for finish-the in thursday<br>(*Such advanced pies, she allowed a boy that trained almost daily to do for the finish last thursday.)
101	SRCE	Sem	Såna avancerade volter tillät en kille som tränade nästan dagligen henne att göra till avslutningen i torsdags.<br>Such advanced somersaults allowed a boy that trained almost daily her to do for finish-the in thursday<br>(*Such advanced somersaults, a boy that trained almost daily allowed her to do for the finish last thursday.)
101	SRCE	NotSem	Såna avancerade pajer tillät en kille som tränade nästan dagligen henne att göra till avslutningen i torsdags.<br>Such advanced pies allowed a boy that trained almost daily her to do for finish-the in thursday<br>(*Such advanced pies, a boy that trained almost daily allowed her to do for the finish last thursday.)
102	RCE	Sem	Såna där möbler bad jag en kollega som renoverade på landet att bära efter matchen i söndags.

			Such there furniture asked I a colleague that renovated on countryside-the to carry after game-the in sunday<br>(*That kind of furniture, I asked a colleague that renovated on the countryside to carry after the game last sunday.)
102	RCE	NotSem	Såna där flyttlådor bad jag en kollega som renoverade på landet att bära efter matchen i söndags.<br>Such there moving-boxes asked I a colleague that renovated on countryside-the to carry after game-the in sunday<br>(*That kind of moving boxes, I asked a colleague that renovated on the countryside to carry after the game last sunday.)
102	SRCE	Sem	Såna där möbler bad en kollega som renoverade på landet mig att bära efter matchen i söndags.<br>Such there furniture asked a colleague that renovated on countryside-the me to carry after game-the in sunday<br>(*That kind of furniture, a colleague that renovated on the countryside asked me to carry after the game last sunday.)
102	SRCE	NotSem	Såna där flyttlådor bad en kollega som renoverade på landet mig att bära efter matchen i söndags.<br>Such there moving-boxes asked a colleague that renovated on countryside-the me to carry after game-the in sunday<br>(*That kind of moving boxes, a colleague that renovated on the countryside asked me to carry after the game last sunday.)
103	RCE	Sem	De där böckerna rekommenderade vi en man som skrev i höstas att citera i artikeln till årsboken.<br>Those there books recommended we a man that wrote in autumn to quote in article-the for yearbook-the<br>(*Those books, we recommended a man that wrote last autumn to quote in the article for the yearbook.)
103	RCE	NotSem	De där teoretikerna rekommenderade vi en man som skrev i höstas att citera i artikeln till årsboken.<br>They there theorists recommended we a man that wrote in autumn to quote in article-the for yearbook-the<br>(*Those theorists, we recommended a man that wrote last autumn to quote in the article for the yearbook.)
103	SRCE	Sem	De där böckerna rekommenderade en man som skrev i höstas oss att citera i artikeln till årsboken.<br>They there books recommended a man that wrote in autumn us to quote in article-the for yearbook-the<br>(*Those books, a man that wrote last autumn recommended us to quote in the article for the yearbook.)
103	SRCE	NotSem	De där teoretikerna rekommenderade en man som skrev i höstas oss att citera i artikeln till årsboken.<br>They there theorists recommended a man that wrote in autumn us to quote in article-the for yearbook-the<br>(*Those theorists, a man that wrote last autumn recommended us to quote in the article for the yearbook.)
104	RCE	Sem	Såna nyttiga limpor beordrade han en dam som bakade förra veckan att

			tina i mikrovågsugnen på låg effekt.<br>Such beneficial loaves ordered he a lady that baked last week-the to unfreeze in microwave-the on low effect<br>(*Such beneficial loaves, he ordered a lady that baked last week to unfreeze in the microwave on low power.)
104	RCE	NotSem	Såna nyttiga morotsbiffar beordrade han en dam som bakade förra veckan att tina i mikrovågsugnen på låg effekt.<br>Such beneficial carrot-steaks ordered he a lady that baked last week-the to unfreeze in microwave-the on low effect<br>(*Such healthy carrot steaks, he ordered a lady that baked last week to unfreeze in the microwave on low power.)
104	SRCE	Sem	Såna nyttiga limpor beordrade en dam som bakade förra veckan honom att tina i mikrovågsugnen på låg effekt.<br>Such beneficial loaves ordered a lady that baked last week-the him to unfreeze in microwave-the on low effect<br>(*Such healthy loaves, a lady that baked last week ordered him to unfreeze in the microwave on low power.)
104	SRCE	NotSem	Såna nyttiga morotsbiffar beordrade en dam som bakade förra veckan honom att tina i mikrovågsugnen på låg effekt.<br>Such beneficial carrot-steaks ordered a lady that baked last week-the him to unfreeze in microwave-the on low effect<br>(*Such healthy carrot steaks, a lady that baked last week ordered him to unfreeze in the microwave on low power.)
105	RCE	Sem	Såna glansiga veckotidningar kommenderade han en tjej som läste på kvällarna att sortera i högar inför storstädningen.<br>Such glossy magazines commanded he a girl that read in evenings-the to sort in piles before big-cleaning-the<br>(*Such glossy magazines, he commanded a girl that read in the evenings to sort in piles before the big cleaning.)
105	RCE	NotSem	Såna glansiga strumpbyxor kommenderade han en tjej som läste på kvällarna att sortera i högar inför storstädningen.<br>Such glossy leggings commanded he a girl that read in evenings-the to sort in piles before big-cleaning-the<br>(*Such glossy leggings, he commanded a girl that read in the evenings to sort in piles before the big cleaning.)
105	SRCE	Sem	Såna glansiga veckotidningar kommenderade en tjej som läste på kvällarna honom att sortera i högar inför storstädningen.<br>Such glossy magazines commanded a girl that read in evenings-the him to sort in piles before big-cleaning-the<br>(*Such glossy magazines, a girl that read in the evenings commanded him to sort in piles before the big cleaning.)
105	SRCE	NotSem	Såna glansiga strumpbyxor kommenderade en tjej som läste på kvällarna honom att sortera i högar inför storstädningen.<br>Such glossy leggings commanded a girl that read in evenings-the him to sort in piles before big-cleaning-the<br>(*Such glossy leggings, a girl that read in the evenings commanded him to sort in piles before the big cleaning.)
106	RCE	Sem	Såna där dokument rådde hon en arbetskamrat som mejlade på lunchen

			att spara tills vidare för säkerhets skull.<br>Such there document adviced she a colleague that emailed at lunch-the to save until further-notice for safety's sake<br>(*That kind of documents, she adviced a colleague that emailed at lunch to save temporarily as a precaution.)
106	RCE	NotSem	Såna där barnkläder rådde hon en arbetskamrat som mejlade på lunchen att spara tills vidare för säkerhets skull.<br>Such there children's-wear adviced she a colleague that emailed at lunch-the to save until further-notice for safety's sake<br>(*That kind of children's wear, she adviced a colleague that emailed at lunch to save temporarily as a precaution.)
106	SRCE	Sem	Såna där dokument rådde en arbetskamrat som mejlade på lunchen henne att spara tills vidare för säkerhets skull.<br>Such there document adviced a colleague that emailed at lunch-the her to save until further-notice for safety's sake<br>(*That kind of documents, a colleague that emailed at lunch adviced her to save temporarily as a precaution.)
106	SRCE	NotSem	Såna där barnkläder rådde en arbetskamrat som mejlade på lunchen henne att spara tills vidare för säkerhets skull.<br>Such there children's-wear adviced a colleague that emailed at lunch-the her to save until further-notice for safety's sake<br>(*That kind of children's wear a colleague that emailed at lunch adviced her to save temporarily as a precaution.)
107	RCE	Sem	Såna där lakan övertalade jag en kompis som manglade ganska ofta att vika i eftermiddags när vi fikade.<br>Such there sheets persuaded I a buddy that mangled quite often to fold in afternoon when we had-coffee<br>(*That kind of sheets, I persuaded a buddy that mangled quite often to fold in the afternoon when we had coffee.)
107	RCE	NotSem	Såna där flygblad övertalade jag en kompis som manglade ganska ofta att vika i eftermiddags när vi fikade.<br>Such there flyer persuaded I a buddy that mangled quite often to fold in afternoon when we had-coffee<br>(*That kind of flyer, I persuaded a buddy that mangled quite often to fold in the afternoon when we had coffee.)
107	SRCE	Sem	Såna där lakan övertalade en kompis som manglade ganska ofta mig att vika i eftermiddags när vi fikade.<br>Such there sheets persuaded a buddy that mangled quite often me to fold in afternoon when we had-coffee<br>(*That kind of sheets, a buddy that mangled quite often persuaded me to fold in the afternoon when we had coffee.)
107	SRCE	NotSem	Såna där flygblad övertalade en kompis som manglade ganska ofta mig att vika i eftermiddags när vi fikade.<br>Such there flyer persuaded a buddy that mangled quite often me to fold in afternoon when we had-coffee<br>(*That kind of flyer, a buddy that mangled quite often persuaded me to fold in the afternoon when we had coffee.)

108	RCE	Sem	De där garderoberna uppmanade jag en släkting som städade väldigt slarvigt att tömma på direkten på grund av lukten.<br>Those there closets urged I a relative that cleaned very sloppily to empty on directly on grounds of smell-the<br>(*Those closets, I urged a relative that cleaned very sloppily to empty instantly because of the smell.)
108	RCE	NotSem	De där matresterna uppmanade jag en släkting som städade väldigt slarvigt att tömma på direkten på grund av lukten.<br>Those there leftovers urged I a relative that cleaned very sloppily to empty on directly on grounds of smell-the<br>(Those leftovers, I urged a relative that cleaned very sloppily to empty instantly because of the smell.)
108	SRCE	Sem	De där garderoberna uppmanade en släkting som städade väldigt slarvigt mig att tömma på direkten på grund av lukten.<br>Those there closets urged a relative that cleaned very sloppily me to empty on directly on grounds of smell-the<br>(*Those closets, a relative that cleaned very sloppily urged me to empty instantly because of the smell.)
108	SRCE	NotSem	De där matresterna uppmanade en släkting som städade väldigt slarvigt mig att tömma på direkten på grund av lukten.<br>Those there leftovers urged a relative that cleaned very sloppily me to empty on directly on grounds of smell-the<br>(Those leftovers, a relative that cleaned very sloppily urged me to empty instantly because of the smell.)
109	RCE	Sem	Såna där mattor pressade vi en farbror som vävde i hemmet att sälja på auktion efter sommaren.<br>Such there carpets pressed we an uncle that weaved at home-the to sell at auction after summer-the<br>(*That kind of carpets, we pressed an uncle that weaved at home to sell at auction after the summer.)
109	RCE	NotSem	Såna där diamantringar pressade vi en farbror som vävde i hemmet att sälja på auktion efter sommaren.<br>Such there diamond-rings pressed we an uncle that weaved at home-the to sell at auction after summer-the<br>(*That kind of diamond rings, we pressed an uncle that weaved at home to sell at auction after the summer.)
109	SRCE	Sem	Såna där mattor pressade en farbror som vävde i hemmet oss att sälja på auktion efter sommaren.<br>Such there carpets pressed an uncle that weaved at home-the us to sell at auction after summer-the<br>(*That kind of carpets, an uncle that weaved at home pressed us to sell at auction after the summer.)
109	SRCE	NotSem	Såna där diamantringar pressade en farbror som vävde i hemmet oss att sälja på auktion efter sommaren.<br>Such there diamond-rings pressed we an uncle that weaved at home-the to sell at auction after summer-the<br>(*That kind of diamond rings, an uncle that weaved at home pressed us to sell at auction after the summer.)

110	RCE	Sem	Såna där cigarrer avrådde hon en kvinna som rökte för jämnan att köpa på nätet utan kvitto.<br>Such there cigars discouraged she a woman that smoked as habit to buy on internet-the without receipt<br>(*That kind of cigars, she discouraged a woman that smoked habitually to buy online without a receipt.)
110	RCE	NotSem	Såna där bestick avrådde hon en kvinna som rökte för jämnan att köpa på nätet utan kvitto.<br>Such there cutlery discouraged she a woman that smoked as habit to buy on internet-the without receipt<br>(*That kind of cutlery, she discouraged a woman that smoked habitually to buy online without a receipt.)
110	SRCE	Sem	Såna där cigarrer avrådde en kvinna som rökte för jämnan henne att köpa på nätet utan kvitto.<br>Such there cigars discouraged a woman that smoked as habit her to buy on internet-the without receipt<br>(*That kind of cigars a woman that smoked habitually discouraged her to buy online without a receipt.)
110	SRCE	NotSem	Såna där bestick avrådde en kvinna som rökte för jämnan henne att köpa på nätet utan kvitto.<br>Such there cutlery discouraged a woman that smoked as habit her to buy on internet-the without receipt<br>(*That kind of cutlery, a woman that smoked habitually discouraged her to buy online without a receipt.)
111	RCE	Sem	Såna stora skålar peppade jag en kompis som drejade riktigt bra att formge till designmässan i påskas.<br>Such large bowls encouraged I a buddy that turned really good to design for design-trade-show-the in easter<br>(*Such large bowls, I encouraged a buddy that turned really good to design for the design trade show last easter.)
111	RCE	NotSem	Såna stora skrivbord peppade jag en kompis som drejade riktigt bra att formge till designmässan i påskas.<br>Such large desk encouraged I a buddy that turned really good to design for design-trade-show-the in easter<br>(*Such large desks, I encouraged a buddy that turned really good to design for the design trade show last easter.)
111	SRCE	Sem	Såna stora skålar peppade en kompis som drejade riktigt bra mig att formge till designmässan i påskas.<br>Such large bowls encouraged a buddy that turned really good me to design for design-trade-show-the in easter<br>(*Such large bowls, a buddy that turned really good encouraged me to design for the design tradeshow last easter.)
111	SRCE	NotSem	Såna stora skrivbord peppade en kompis som drejade riktigt bra mig att formge till designmässan i påskas.<br>Such large desks encouraged a buddy that turned really good me to design for design-trade-show-the in easter<br>(Such large desks a buddy that turned really good encouraged me to

design for the design tradeshow last easter.)

112 RCE Sem De där öronmuffarna sporrade han en bekant som stickade framför TVn att använda på skoj ovanpå mössan.
Those there ear-warmers spurred he an acquaintance that knitted in-front-of tv-the to use for fun on-top-of hat-the
(*Those ear warmers, he spurred an acquaintance that knitted in front of the tv to use for fun on top of his hat.)

112 RCE NotSem De där hörlurarna sporrade han en bekant som stickade framför TVn att använda på skoj ovanpå mössan.
Those there headphones spurred he an acquaintance that knitted in-front-of tv-the to use for fun on-top-of hat-the
(*Those headphones, he spurred an acquaintance that knitted in front of the tv to use for fun on top of his hat.)

112 SRCE Sem De där öronmuffarna sporrade en bekant som stickade framför TVn honom att använda på skoj ovanpå mössan.
Those there ear-warmers spurred an acquaintance that knitted in-front-of tv-the him to use for fun on-top-of hat-the
(*Those ear warmers, an acquaintance that knitted in front of the tv spurred him to use for fun on top of his hat.)

112 SRCE NotSem De där hörlurarna sporrade en bekant som stickade framför TVn honom att använda på skoj ovanpå mössan.
Those there headphones spurred an acquaintance that knitted in-front-of tv-the him to use for fun on-top-of hat-the
(*Those headphones, an acquaintance that knitted in front of the tv spurred him to use for fun on top of his hat.)

113 RCE Sem Såna där trägolv inspirerade hon en vän som dammsög varje vecka att slipa med omsorg efter ett halvår.
Such there hardwood-floor inspired she a friend that vacuumed every week to hone with care after one half-year
(*That kind of hardwood floors, she inspired a friend that vacuumed cleaned every week to sand with care after six months.)

113 RCE NotSem Såna där knivar inspirerade hon en vän som dammsög varje vecka att slipa med omsorg efter ett halvår.
Such there knives inspired she a friend that vacuumed every week to hone with care after one half-year
(*That kind of knives, she inspired a friend that vacuumed cleaned every week to hone with care after six months.)

113 SRCE Sem Såna där trägolv inspirerade en vän som dammsög varje vecka henne att slipa med omsorg efter ett halvår.
Such there hardwood-floor inspired a friend that vacuumed every week her to hone with care after one half-year
(*That kind of hardwood floors, a friend that vacuumed cleaned every week inspired her to sand with care after six months.)

113 SRCE NotSem Såna där knivar inspirerade en vän som dammsög varje vecka henne att slipa med omsorg efter ett halvår.
Such there knives inspired a friend that vacuumed every week her to hone with care after one half-year
(*That kind of knives a friend that vacuumed cleaned every week

			inspired her to hone with care after six months.)
114	RCE	Sem	Såna där byxor nekade hon en kvinna som sydde på beställning att låna inför resan nästa vecka.<br>Such there pants denied she a woman that sewed on demand to borrow for trip-the next week<br>(*That kind of pants, she denied a woman that sewed on demand to borrow for the trip next week.)
114	RCE	NotSem	Såna där summor nekade hon en kvinna som sydde på beställning att låna inför resan nästa vecka.<br>Such there sums denied she a woman that sewed on demand to borrow for trip-the next week<br>(*That kind of sums, she denied a woman that sewed on demand to borrow for the trip next week.)
114	SRCE	Sem	Såna där byxor nekade en kvinna som sydde på beställning henne att låna inför resan nästa vecka.<br>Such there pants denied a woman that sewed on demand her to borrow for trip-the next week<br>(*That kind of pants, a woman that sewed on demand denied her to borrow for the trip next week.)
114	SRCE	NotSem	Såna där summor nekade en kvinna som sydde på beställning henne att låna inför resan nästa vecka.<br>Such there sums denied a woman that sewed on demand her to borrow for trip-the next week<br>(*That kind of sums, a woman that sewed on demand denied her to borrow for the trip next week.)
115	RCE	Sem	De där plankorna hindrade han en granne som sågade i källaren att slänga i onödan i förrgår.<br>Those there planks prevented he a neighbor that sawed in basement-the to throw-away in unnecessarily in day-before-yesterday<br>(*Those planks, he prevented a neighbor that sawed in the basement to throw away unnecessarily the day before yesterday.)
115	RCE	NotSem	De där tepåsarna hindrade han en granne som sågade i källaren att slänga i onödan i förrgår.<br>Those there teabags prevented he a neighbor that sawed in basement-the to throw-away in unnecessarily in day-before-yesterday<br>(*Those teabags, he prevented a neighbor that sawed in the basement to throw away unnecessarily the day before yesterday.)
115	SRCE	Sem	De där plankorna hindrade en granne som sågade i källaren honom att slänga i onödan i förrgår.<br>Those there planks prevented a neighbor that sawed in basement-the him to throw-away in unnecessarily in day-before-yesterday<br>(*Those planks, a neighbor that sawed in the basement prevented him to throw away unnecessarily the day before yesterday.)
115	SRCE	NotSem	De där tepåsarna hindrade en granne som sågade i källaren honom att slänga i onödan i förrgår.<br>Those there teabags prevented a neighbor that sawed in basement-the him to throw-away in unnecessarily in day-before-yesterday

			(*Those teabags, a neighbor that sawed in the basement prevented him to throw away unnecessarily the day before yesterday.)
116	RCE	Sem	De där lådorna tvingade jag en kusin som snickrade i skolan att stapla i köket före kalaset.<br>Those there boxes forced I a cousin that crafted in school-the to stack in kitchen-the before party-the<br>(*Those boxes, I forced a cousin that crafted in school to stack in the kitchen before the party.)
116	RCE	NotSem	De där flingpaketen tvingade jag en kusin som snickrade i skolan att stapla i köket före kalaset.<br>Those there cereal-boxes forced I a cousin that crafted in school-the to stack in kitchen-the before party-the<br>(*Those cereal boxes, I forced a cousin that crafted in school to stack in the kitchen before the party.)
116	SRCE	Sem	De där lådorna tvingade en kusin som snickrade i skolan mig att stapla i köket före kalaset.<br>Those there boxes forced a cousin that crafted in school-the me to stack in kitchen-the before party-the<br>(*Those boxes, a cousin that crafted in school forced me to stack in the kitchen before the party.)
116	SRCE	NotSem	De där flingpaketen tvingade en kusin som snickrade i skolan mig att stapla i köket före kalaset.<br>Those there cereal-boxes forced a cousin that crafted in school-the me to stack in kitchen-the before party-the<br>(*Those cereal boxes, a cousin that crafted in school forced me to stack in the kitchen before the party.)
117	RCE	Sem	Såna gamla textilier lejde hon en karl som tvättade för hand att inspektera i tisdags för trehundra kronor.<br>Such old textiles hired she a man that washed by hand to inspect in tuesday for three-hundred crowns<br>(*Such old textiles, she hired a man that washed manually to inspect last tuesday for three hundred crowns.)
117	RCE	NotSem	Såna gamla hyreskontrakt lejde hon en karl som tvättade för hand att inspektera i tisdags för trehundra kronor.<br>Such old rental-agreements hired she a man that washed by hand to inspect in tuesday for three-hundred crowns<br>(*Such old rental agreements, she hired a man that washed manually to inspect last tuesday for three hundred crowns.)
117	SRCE	Sem	Såna gamla textilier lejde en karl som tvättade för hand henne att inspektera i tisdags för trehundra kronor.<br>Such old textiles hired a man that washed by hand her to inspect in tuesday for three-hundred crowns<br>(*Such old textiles, a man that washed manually hired her to inspect last tuesday for three hundred crowns.)
117	SRCE	NotSem	Såna gamla hyreskontrakt lejde en karl som tvättade för hand henne att inspektera i tisdags för trehundra kronor.<br>Such old rental-argeements hired a man that washed by hand her to inspect in tuesday for three-hundred crowns

			(*Such old rental agreements, a man that washed manually hired her to inspect last tuesday for three hundred crowns.)
118	RCE	Sem	Såna där fönster kontrakterade jag en bekant som målade ganska regelbundet att laga i fredags efter arbetet.<br>Such there windows contracted I an acquaintance that painted quite regularly to fix in friday after work-the<br>(*That kind of windows, I contracted an acquaintance that painted quite regularly to fix friday after work.)
118	RCE	NotSem	Såna där punkteringar kontrakterade jag en bekant som målade ganska regelbundet att laga i fredags efter arbetet.<br>Such there punctures contracted I an acquaintance that painted quite regularly to fix in friday after work-the<br>(*That kind of punctures, I contracted an acquaintance that painted quite regularly to fix friday after work.)
118	SRCE	Sem	Såna där fönster kontrakterade en bekant som målade ganska regelbundet mig att laga i fredags efter arbetet.<br>Such there windows contracted an acquaintance that painted quite regularly me to fix in friday after work-the<br>(That kind of windows, an acquaintance that painted quite regularly contracted me to fix friday after work.)
118	SRCE	NotSem	Såna där punkteringar kontrakterade en bekant som målade ganska regelbundet mig att laga i fredags efter arbetet.<br>Such there punctures contracted an acquaintance that painted quite regularly me to fix in friday after work-the<br>(*That kind of punctures, an acquaintance that painted quite regularly contracted me to fix friday after work.)
119	RCE	Sem	Såna där modeller erbjöd vi en person som ritade på helgerna att marknadsföra i media under julhandeln.<br>Such there models offered we a person that drew on weekends-the to promote in media during christmas-shopping-season-the<br>(*That kind of models, we offered a person that drew during the weekends to promote in the media during the christmas shopping season.)
119	RCE	NotSem	Såna där dofter erbjöd vi en person som ritade på helgerna att marknadsföra i media under julhandeln.<br>Such there fragrances offered we a person that drew on weekends-the to promote in media during christmas-shopping-season-the<br>(*That kind of fragrances, we offered a person that drew during the weekends to promote in the media during the christmas shopping season.)
119	SRCE	Sem	Såna där modeller erbjöd en person som ritade på helgerna oss att marknadsföra i media under julhandeln.<br>Such there models offered a person that drew on weekends-the us to promote in media during christmas-shopping-season-the<br>(*That kind of models, a person that drew during the weekends offered us to promote in the media during the christmas shopping season.)
119	SRCE	NotSem	Såna där dofter erbjöd en person som ritade på helgerna oss att marknadsföra i media under julhandeln.<br>Such there fragrances offered we a person that drew on weekends-the to

			promote in media during christmas-shopping-season-the<br>(*That kind of fragrances, a person that drew during the weekends offered us to promote in the media during the christmas shopping season.)
120	RCE	Sem	Såna där ölglas lockade vi en väninna som diskade på krogen att beställa från utlandet en sen kväll.<br>Such there beer-glas tempted we a friend that washed at pub-the to order from abroad a late night<br>(*That kind of beer glasses, we tempted a friend that washed at the pub to order from abroad a late night.)
120	RCE	NotSem	Såna där talböcker lockade vi en väninna som diskade på krogen att beställa från utlandet en sen kväll.<br>Such there audio-books tempted we a friend that washed at pub-the to order from abroad a late night<br>(*That kind of audio books, we we a friend that washed at the pub to order from abroad a late night.)
120	SRCE	Sem	Såna där ölglas lockade en väninna som diskade på krogen oss att beställa från utlandet en sen kväll.<br>Such there beer-glas tempted a friend that washed at pub-the us to order from abroad a late night<br>(That kind of beer glasses, a friend that washed at the pub tempted us to order from abroad a late night.)
120	SRCE	NotSem	Såna där talböcker lockade en väninna som diskade på krogen oss att beställa från utlandet en sen kväll.<br>Such there audio-books tempted a friend that washed at pub-the us to order from abroad a late night<br>(*That kind of audio books, a friend that washed at the pub tempted us to order from abroad a late night.)
121	RCE	Sem	Den där bilen lurade han en person som körde till jobbet att hyra förra året på semestern.<br>That there car cheated he a person that drove to work-the to rent last year on vacation-the<br>(*That car, he cheated a person that drove to work to rent last year on vacation.)
121	RCE	NotSem	Den där lägenheten lurade han en person som körde till jobbet att hyra förra året på semestern.<br>That there apartment cheated he a person that drove to work-the to rent last year on vacation-the<br>(*That apartment, he cheated a person that drove to work to rent last year on vacation.)
121	SRCE	Sem	Den där bilen lurade en person som körde till jobbet honom att hyra förra året på semestern.<br>That there car cheated a person that drove to work-the him to rent last year on vacation-the<br>(*That car, a person that drove to work cheated him to rent last year on vacation.)
121	SRCE	NotSem	Den där lägenheten lurade en person som körde till jobbet honom att hyra förra året på semestern.

			That there car apartment a person that drove to work-the him to rent last year on vacation-the<br>(That apartment, a person that drove to work cheated him to rent last year on vacation.)
122	RCE	Sem	En sån mössa förledde hon en tant som virkade väldigt snabbt att införskaffa på rean efter jul.<br>A such hat deceived she a lady that crocheted very fast to get on sale-the after christmas<br>(*That kind of hat, she deceived a lady that crocheted very fast to get on sale after christmas.)
122	RCE	NotSem	En sån tv-apparat förledde hon en tant som virkade väldigt snabbt att införskaffa på rean efter jul.<br>A such television-apparatus deceived she a lady that crocheted very fast to get on sale-the after christmas<br>(*That kind of television apparatus, she deceived a lady that crocheted very fast to get on sale after christmas.)
122	SRCE	Sem	En sån mössa förledde en tant som virkade väldigt snabbt henne att införskaffa på rean efter jul.<br>A such hat deceived a lady that crocheted very fast her to get on sale-the after christmas<br>(*That kind of hat, a lady that crocheted very fast deceived her to get on sale after christmas.)
122	SRCE	NotSem	En sån tv-apparat förledde en tant som virkade väldigt snabbt henne att införskaffa på rean efter jul.<br>A such television-apparatus deceived a lady that crocheted very fast her to get on sale-the after christmas<br>(*That kind of television apparatus, a lady that crocheted very fast deceived her to get on sale after christmas.)
123	RCE	Sem	Såna där bullar frestade han en kompis som åt hela tiden att inhandla på konsum i måndags.<br>Such there buns tempted he a buddy that are all time-the to purchase on coop in monday<br>(*That kind of buns, he tempted a buddy that ate continuously to purchase at coop last monday.)
123	RCE	NotSem	Såna där vantar frestade han en kompis som åt hela tiden att inhandla på konsum i måndags.<br>Such there mittens tempted he a buddy that are all time-the to purchase on coop in monday<br>(*That kind of mittens, he tempted a buddy that ate continuously to purchase at coop last monday.)
123	SRCE	Sem	Såna där bullar frestade en kompis som åt hela tiden honom att inhandla på konsum i måndags.<br>Such there buns tempted a buddy that are all time-the him to purchase on coop in monday<br>(*That kind of buns, a buddy that ate continuously tempted him to purchase at coop monday.)
123	SRCE	NotSem	Såna där vantar frestade en kompis som åt hela tiden honom att inhandla

			på konsum i måndags.<br>Such there mittens tempted a buddy that are all time-the him to purchase on coop in monday<br>(*That kind of mittens, a buddy that ate continuously tempted him to purchase at coop monday.)
124	RCE	Sem	Såna där vikväggar anmodade han en person som tapetserade hela våren att tillverka till eventet på sportlovet.<br>Such there folding-walls requested he a person that wallpapered whole spring-the to manufacture for event-the on sports-holiday<br>(*That kind of folding walls, he requested a person that wallpapered throughout the spring to manufacture to the event during the sports holiday.)
124	RCE	NotSem	Såna där bakverk anmodade han en person som tapetserade hela våren att tillverka till eventet på sportlovet.<br>Such there pastry requested he a person that wallpapered whole spring-the to manufacture for event-the on sports-holiday<br>(*That kind of pastry, he requested a person that wallpapered throughout the spring to manufacture to the event during the sports holiday.)
124	SRCE	Sem	Såna där vikväggar anmodade en person som tapetserade hela våren honom att tillverka till eventet på sportlovet.<br>Such there folding-walls requested a person that wallpapered whole spring-the him to manufacture for event-the on sports-holiday<br>(*That kind of folding walls, a person that wallpapered throughout the spring requested him to manufacture to the event during the sports holiday.)
124	SRCE	NotSem	Såna där bakverk anmodade en person som tapetserade hela våren honom att tillverka till eventet på sportlovet.<br>Such there pastry requested a person that wallpapered whole spring-the him to manufacture for event-the on sports-holiday<br>(*That kind of pastry, a person that wallpapered throughout the spring requested him to manufacture to the event during the sports holiday.)
125	RCE	Sem	Såna där glas förhindrade jag en tjej som packade rätt slarvigt att spräcka av misstag häromdagen.<br>Such there glas prevented I a girl that packed pretty carelessly to burst by accident the-other-day<br>(*That kind of glasses, I prevented a girl that packed pretty carelessly to burst by accident the other day.)
125	RCE	NotSem	Såna där sömmar förhindrade jag en tjej som packade rätt slarvigt att spräcka av misstag häromdagen.<br>Such there seams prevented I a girl that packed pretty carelessly to burst by accident the-other-day<br>(*That kind of seams, I prevented a girl that packed pretty carelessly to burst by accident the other day.)
125	SRCE	Sem	Såna där glas förhindrade en tjej som packade rätt slarvigt mig att spräcka av misstag häromdagen.<br>Such there glas prevented a girl that packed pretty carelessly me to burst by accident the-other-day<br>(*That kind of glasses, a girl that packed pretty carelessly prevented me

			to burst by accident the other day.)
125	SRCE	NotSem	Såna där sömmar förhindrade en tjej som packade rätt slarvigt mig att spräcka av misstag häromdagen.<br>Such there seams prevented a girl that packed pretty carelessly me to burst by accident the-other-day<br>(*That kind of seams, a girl that packed pretty carelessly prevented me to burst by accident the other day.)
126	RCE	Sem	Sånt svindyrt vin provocerade vi en kollega som drack rätt ofta att handla inför helgen trots priset.<br>Such expensive wine provoked we a colleague that drank quite often to shop for weekend-the despite price-the<br>(*Such expensive wine, we provoked a colleague that drank quite often to shop for the weekend despite the price.)
126	RCE	NotSem	Sånt svindyrt salt provocerade vi en kollega som drack rätt ofta att handla inför helgen trots priset.<br>Such expensive salt provoked we a colleague that drank quite often to shop for weekend-the despite price-the<br>(*Such expensive salt, we provoked a colleague that drank quite often to shop for the weekend despite the price.)
126	SRCE	Sem	Sånt svindyrt vin provocerade en kollega som drack rätt ofta oss att handla inför helgen trots priset.<br>Such expensive wine provoked a colleague that drank quite often us to shop for weekend-the despite price-the<br>(*Such expensive wine, a colleague that drank quite often provoked us to shop for the weekend despite the price.)
126	SRCE	NotSem	Sånt svindyrt salt provocerade en kollega som drack rätt ofta oss att handla inför helgen trots priset.<br>Such expensive salt provoked a colleague that drank quite often us to shop for weekend-the despite price-the<br>(*Such expensive salt, a colleague that drank quite often provoked us to shop for the weekend despite the price.)
127	RCE	Sem	Den där gardinen bönföll hon en kompis som strök varje vecka att kasta i återvinningen i lördags.<br>That there curtain begged she a buddy that ironed every week to throw in recycling-station in saturday<br>(*That curtain, she begged a buddy that ironed every week to throw at the recycling staion last saturday.)
127	RCE	NotSem	Den där cd-spelaren bönföll hon en kompis som strök varje vecka att kasta i återvinningen i lördags.<br>That there cd-player begged she a buddy that ironed every week to throw in recycling-station in saturday<br>(*That cd player, she begged a buddy that ironed every week to throw at the recycling staion last saturday.)
127	SRCE	Sem	Den där gardinen bönföll en kompis som strök varje vecka henne att kasta i återvinningen i lördags.<br>That there curtain begged a buddy that ironed every week her to throw in recycling-station in saturday

			(* That curtain, a buddy that ironed every week begged her to throw at the recycling staion last saturday.)
127	SRCE	NotSem	Den där cd-spelaren bönföll en kompis som strök varje vecka henne att kasta i återvinningen i lördags.<br>That there cd-player begged a buddy that ironed every week her to throw in recycling-station in saturday<br>(That cd player, a buddy that ironed every week begged her to throw at the recycling staion last saturday.)
128	RCE	Sem	Såna där kräftor fick jag en granne som fiskade varje sommar att skölja en minut i kallvatten.<br>Such there crawfish got I a neighbor that fished every summar to rinse one minute in cold-water<br>(*That kind of crawfish, I got a neighbor that fished every summer to rinse one minute in cold water.)
128	RCE	NotSem	Såna där morötter fick jag en granne som fiskade varje sommar att skölja en minut i kallvatten.<br>Such there carrots got I a neighbor that fished every summar to rinse one minute in cold-water<br>(*That kind of carrots, I got a neighbor that fished every summer to rinse one minute in cold water.)
128	SRCE	Sem	Såna där kräftor fick en granne som fiskade varje sommar mig att skölja en minut i kallvatten.<br>Such there crawfish got a neighbor that fished every summar me to rinse one minute in cold-water<br>(*That kind of crawfish, a neighbor that fished every summer got me to rinse one minute in cold water.)
128	SRCE	NotSem	Såna där morötter fick en granne som fiskade varje sommar mig att skölja en minut i kallvatten.<br>Such there carrots got a neighbor that fished every summar me to rinse one minute in cold-water<br>(That kind of carrots, a neighbor that fished every summer got me to rinse one minute in cold water.)
